# Adverse Events Relating to Prolonged Hard Collar Immobilisation: A Systematic Review and Meta-Analysis

**DOI:** 10.1177/21925682221087194

**Published:** 2022-03-25

**Authors:** Jamie F.M. Brannigan, Esmee Dohle, Giles R. Critchley, Rikin Trivedi, Rodney J. Laing, Benjamin M. Davies

**Affiliations:** 1 Division of Neurosurgery, Department of Clinical Neurosciences, 2153 University of Cambridge, Cambridge, UK; 2 School of Clinical Medicine, 2152 University of Cambridge, Cambridge, UK; 3 Department of Neurosurgery, 8721 Brighton and Sussex University Hospitals National Health Service Trust, Brighton, UK; 4 Myelopathy.org, University of Cambridge, UK

**Keywords:** cervical, vertebrae, trauma, post-operative, hard collar, orthosis, complications, pressure ulcers, dysphagia, systematic review, meta-analysis

## Abstract

**Study Design:**

Systematic review and meta-analysis.

**Objective:**

To evaluate systematically the complications of prolonged cervical immobilisation in a hard collar.

**Methods:**

Following registration with PROSPERO, a systematic search of electronic databases (MEDLINE, EMBASE) was conducted. Two reviewers independently screened the search results according to pre-determined search criteria. Data was extracted and tabulated. Joanna Briggs Institute checklists were used for assessing the quality of included studies.

**Results:**

The search identified 773 articles. A total of 25 studies were selected for final inclusion. The results largely comprised a mixture of case reports/series, cohort studies and reviews. The most commonly reported complications were pressure ulcers, dysphagia and increased intracranial pressure. A pressure ulcer pooled prevalence of 7% was calculated. There was insufficient data for quantitative analysis of any other complication.

**Conclusions:**

There is significant morbidity from prolonged hard collar immobilisation, even amongst younger patients. Whilst based upon limited and low-quality evidence, these findings, combined with the low-quality evidence for the efficacy of hard collars, highlights a knowledge gap for future research.

## Introduction

The prolonged use of rigid cervical collars is common, either as part of non-operative management of spinal trauma, but also as an adjunct to cervical spine surgery.^1^ It is estimated 13/100,000 patients undergo non-operative management of a cervical spine fracture each year.^2^ The frequency of their use following cervical spine surgery is less well defined.^3^ When used for either indication, immobilisation is often for at least 4 weeks.^3-5^

Prolonged periods of hard collar use were defined in this study as ≥2 days of wear, to distinguish from emergency immobilisation. Prolonged use can lead to a range of adverse effects, including pressure ulceration, raised intracranial pressure (ICP) and dysphagia.^6^ These complications, which can occur in both inpatients and outpatients, can cause morbidity which may be avoidable.^6,7^

Recent studies have questioned the effectiveness of using hard collars post-operatively.^8,9^ Compounded by a lack of recognition of the potential complications, this has resulted in inconsistent guidance from surgeons.^3^

Although some reviews have been published detailing adverse events and quality improvement protocols,^6,10-12^ there is no systematic review providing a comprehensive analysis of the complications of prolonged use.

The objective of this systematic review is to characterise the frequency and factors associated with complications the results of which should support decision-making by healthcare professionals.

## Method

A systematic review of the literature was performed, compliant with the preferred reporting items of systematic reviews and meta-analysis (PRISMA) guidelines (Supplementary Data 1) and prospectively registered with PROSPERO (CRD42021247869).

### Search Strategy and Search Criteria

**Figure 1.**
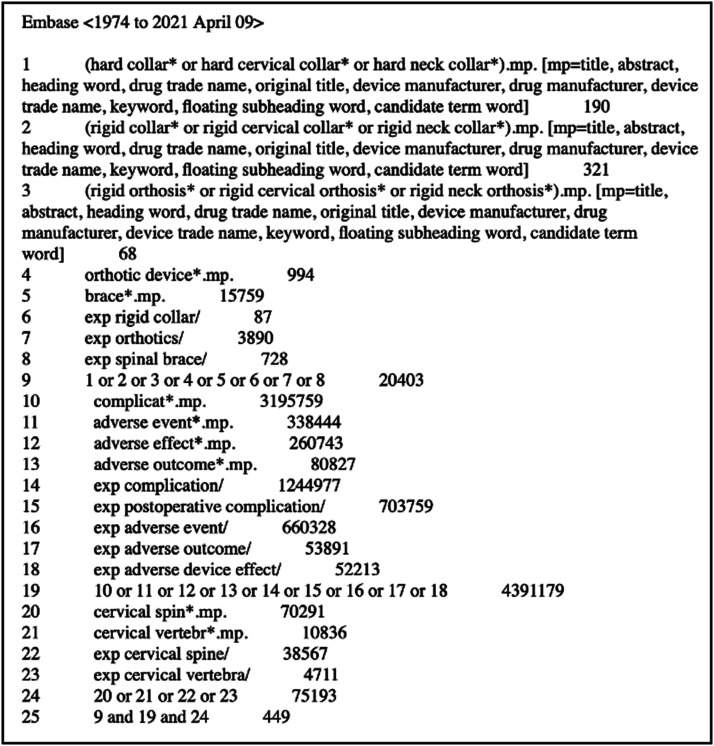
Embase search strategy.

A sensitive search strategy (Supplementary Data 2) was developed with a medical librarian for EMBASE and MEDLINE. Searches were performed using Ovid (Wolters Kluwer, Netherlands) from inception to 9th April 2021. An example of the terms used in the Embase search is shown in Figure 1.

Articles in English, of any nature, reporting on the complications related to prolonged hard collar use were included (Table 1). For the purpose of this study, ‘prolonged use’ was defined as the wearing of a hard collar on at least two consecutive days, thereby distinguishing from cases of emergency immobilisation. Two reviewers (J.B and E.D) independently performed title and abstract screening with blinding, using Rayyan.^13^ A pilot screen of 50 publications was first done to ensure concordance and any disagreements following unblinding were resolved by discussion.

**Table 1.** Inclusion and exclusion criteria.

Inclusion	Exclusion
English, full text	Other forms of orthosis (e.g., Halo, SOMI, soft collar) in isolation
Adults (18 years and above)	
Reporting on complications of orthosis related to prolonged use of a rigid collar, where prolonged is defined as wear on at least two consecutive days	Animal studies
	Cadaveric studies
	Children and youths (up to and including 17 years)
	Where the primary focus is acute care with no follow-up
	Editorials, letters, replies

Screening for eligibility occurred in accordance with the criteria in Table 1.

### Data Extraction and Critical Appraisal

Articles were retrieved for full-text screening and data extraction using a piloted table.

The Joanna Briggs Institute (JBI) critical assessment tools were chosen on the basis of wide variations in study design, the absence of large randomised control trials and the inclusion of case reports.^14^ JBI checklists were completed to assess the quality of the included articles (Supplementary Data 3).

Full-text screening and data extraction were performed by the same two reviewers. Any differences were reconciled through discussion and consensus.

### Quantitative Analysis

Meta-analysis was performed where two or more primary studies reported on the prevalence of the same complication. Analysis was performed using R (v4.0.5; R Core Team, 2020) and RStudio (v1.4.1106; RStudio Team, 2021). The R packages ‘meta’ (v4.18-0)^15^ and ‘ggplot2’ (v3.3.3)^16^ were used to generate pooled prevalence estimates and explore associations using bivariate regression, respectively.

## Results

### Search Strategy

**Figure 2.**
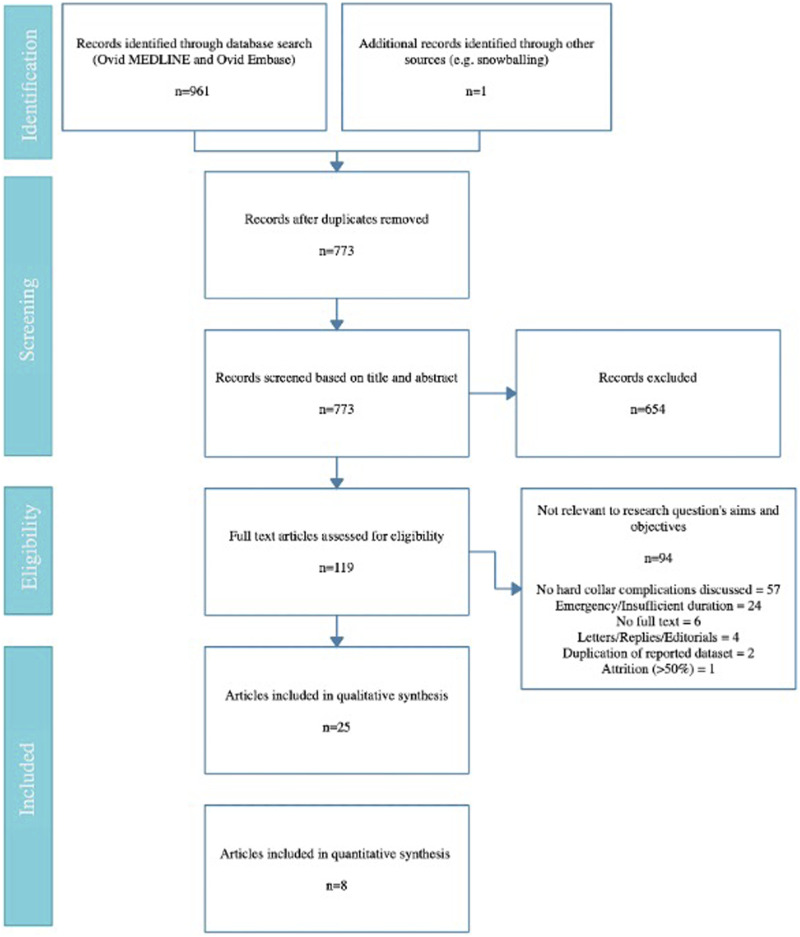
PRISMA flow diagram of the search strategy.

After removing duplicates, the initial search identified 773 articles. Subsequent abstract and title screening eliminated 654 articles, leaving 119 shortlisted for full text review. Of these, 25 were included in this study (Figure 2). A meta-analysis was performed on a subsection of the included articles (n = 8).

A kappa (κ) value of .69 was calculated for title and abstract screening and a value of .95 for full text screening. There was thus a substantial degree of inter-rater reliability. Furthermore, there was complete concordance for data extraction and quality assessment.

### Study Characteristics

Alongside 5 narrative reviews and 1 systematic review, 19 primary clinical studies were identified. This included 13 cohort studies, 4 case reports/series, 1 randomised trial and 1 economic evaluation. Of the primary clinical studies, the mean patient age ranged from 26.8 to 84 and publication years were between 1991 and 2020.

The majority (12/19) of the primary research included was conducted entirely in an inpatient setting. The exceptions were 2 studies involving volunteers^17,18^ and 5 studies with outpatient follow up.^5,7,19-21^ Three studies exclusively recruited patients from critical care.^22-24^ Most studies reported on skin breakdown/ulceration (17/25), and to lesser extent dysphagia (6/25) and increased ICP (4/25).

An evidence summary for the included cohort studies is shown in Table 2. The evidence summary table for the remaining studies can be found in Supplementary Data 4.

**Table 2.** Evidence summary table of the included cohort studies.

Authors	Year	Country	Mean Patient Age, yrs	Collar Indication	Collar Type	Reported Complications	Frequency of Complications	Mean Duration of Wear, Days
Moran et al.	2013	Australia	NR	Trauma	Aspen	Dysphagia Delirium LRTI Falls	4/8 (50%) 3/8 (38%) 5/8 (63%) 3/8 (38%)	NR^d^
Molinari et al.	2012	USA	84	Trauma	Miami J	Skin breakdown/ulceration	2/34 (6%)	84 (SD = 0)
Powers et al.	2006	USA	37.8	Trauma	Aspen	Skin breakdown/ulceration	33/484 (7%)	10.3 (SD = 11.4)
Molano et al.	2004	Spain	35.5	Trauma	NR	Skin breakdown/ulceration	22/92 (24%)	NR^e^
Nakanishi et al.	2019	Australia	81.9	Trauma	Philadelphia	Skin breakdown/ulceration HAP	11/90 (12%) 10/90 (11%)	NR
Hylton et al.	2016	UK	74	Trauma	Miami J	Psychosocial issues Skin breakdown/ulceration Skin erythema	6/51 (12%) 2/51 (4%) 10/51 (20%)	50.8
Ham et al.	2014	USA	43.7	Trauma	Various	Skin breakdown/ulceration	1/88 (1%)	3.5 (SD = 3.4)
Lewis et al.	2011	Australia	NR	Trauma	NR	Skin breakdown/ulceration	2/32 (6%)	NR^f^
Ackland et al.	2007	Australia	40.9^a^	Trauma	Philadelphia	Skin breakdown/ulceration	27/299 (9%)	NR^g^
Karlberg et al.	1991	Sweden	26.8	Volunteer	Philadelphia	Impaired voluntary saccades	NR	5
Borders et al.	2018	USA	NR^b^	Trauma	NR	Dysphagia	104/113 (92%)	NR
Kalb et al.	2012	USA	51	Post-operative	NR	Dysphagia	NR^c^	NR
Iizuka et al.	2005	Japan	61.4	Post-operative	Philadelphia	Reduced cervical ROM	NR	42^h^

^a^Mean male age = 38.2 yrs and mean female age = 43.5 yrs; assumption of equal proportion.

^b^A majority (58%) of patients were under 65 yrs.

^c^There were 27 cases of dysphagia reported, with no significant increase in the hard collar group.

^d^Duration of immobilisation = 2–12 weeks.

^e^Median day of ulcer detection = 7.

^f^Mean duration of all patients = 94.5 days.

^g^Median duration = 1 day (IQR = 2).

^h^One group 8 weeks in collar and the other 4 weeks in collar.

NR = not reported, LRTI = lower respiratory tract infection, HAP = hospital acquired pneumonia, ROM = range of motion

### Pressure Ulceration

Eight primary clinical studies reported on the occurrence of skin breakdown/ulceration. These studies cumulatively include 1,170 patient outcomes and the mean duration of hard collar immobilisation ranged from 3.5 days to 84 days. A more detailed description of these studies is shown in in Table 3.

**Table 3.** A subgroup evidence table of studies reporting on pressure ulcer frequency.

Authors	Year	Mean Patient Age, Years	Specific Trauma Indication	Collar Type	Skin Breakdown Frequency	Outpatient Follow up	Mean Duration of Wear, Days	Nursing Measures
Molinari et al.	2012	84 (Range = 71–99)	Type II odontoid fracture	Miami J	2/34 (6%)	Yes	84 (SD = 0)	NR
Powers et al.	2006	37.8 (Range = 2–94)	Confirmed cervical injury, ICU	Aspen	33/484 (7%)	No	10.3 (SD = 11.4)	Pad change every 24 hrs, skin clean every 12 hrs
Molano et al.	2004	35.5 (SD = 13.8)	Suspected cervical injury, ICU	NR	22/92 (24%)	No	NR^c^	NR
Nakanishi et al.	2019	81.9 (SD = 8.6)	Suspected cervical injury	Philadelphia	11/90 (12%)	No	NR	Removed, cleaned and dried every 4–8 hrs
Hylton et al.	2016	74 (Range = 21–95)	Confirmed cervical injury	Miami J	2/51 (4%)	Yes	50.8	Removed and adjusted weekly (outpatient)
Ham et al.	2014	43.7 (SD = 19.6; Range = 18–91)	Suspected cervical injury, ICU	Various	1/88 (1%)	No	3.5 (SD = 3.4)	Pressure relieving mattress, turning every 2 hrs, occipital foam ring
Lewis et al.	2011	NR	Type II odontoid fracture	NR	2/32 (6%)	Yes	NR^d^	NR
Ackland et al.	2007	40.9 (SD = 18.6)^a^	Suspected cervical injury	Philadelphia	27/299 (9%)	No	NR^e^	Patient position changed every 8 hrs. Removed, cleaned and dried every 4–8 hrs

^a^Mean male age = 38.2 ± 16.7 years and mean female age = 43.5 ± 20.4 years; assumption of equal proportion.

^b^A majority (58%) of patients were under 65 years.

^c^Median day of ulcer detection = 7.

^d^Mean duration of all patients = 94.5 days.

^e^Median duration = 1 day (IQR = 2).

SD = standard deviation, NR = not reported, ICU = intensive care unit, hrs = HoursGrey = excluded from age regression analysis.

**Figure 3.**
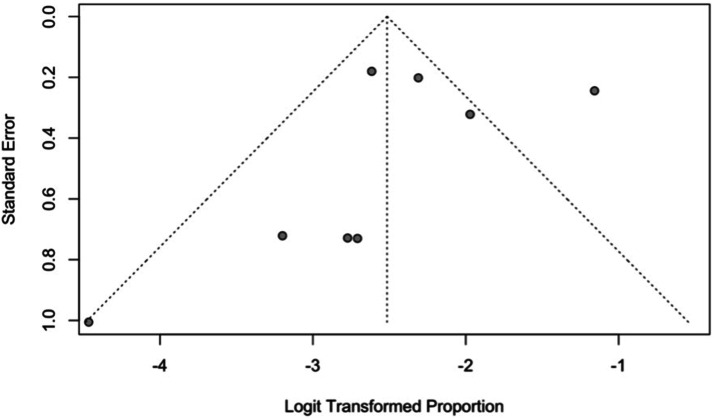
An asymmetric funnel plot displaying study heterogeneity.

Reported incidence across studies displayed high heterogeneity (*I*^*2*^ = 78%; Figure 3).

**Figure 4.**
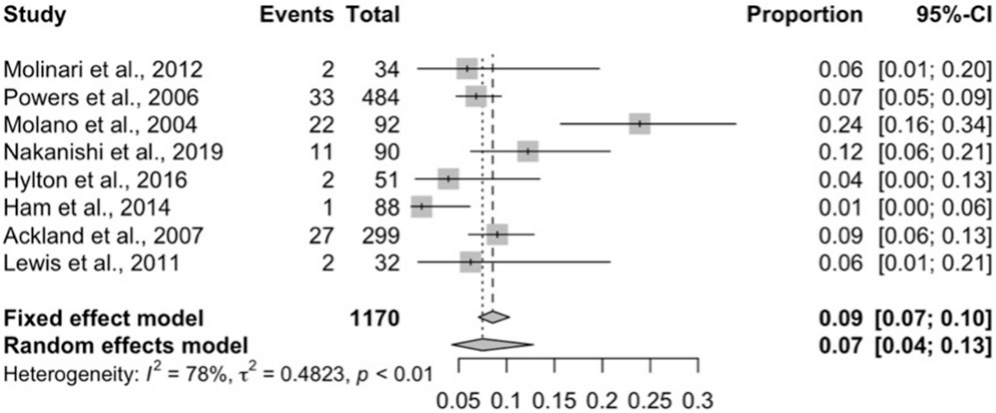
A pooled prevalence analysis of pressure ulcers in cases of prolonged hard collar immobilisation; random effects model.

Using a random effects model, the pooled prevalence was estimated as 7% [95% CI: 4–13%] (Figure 4).

**Figure 5.**
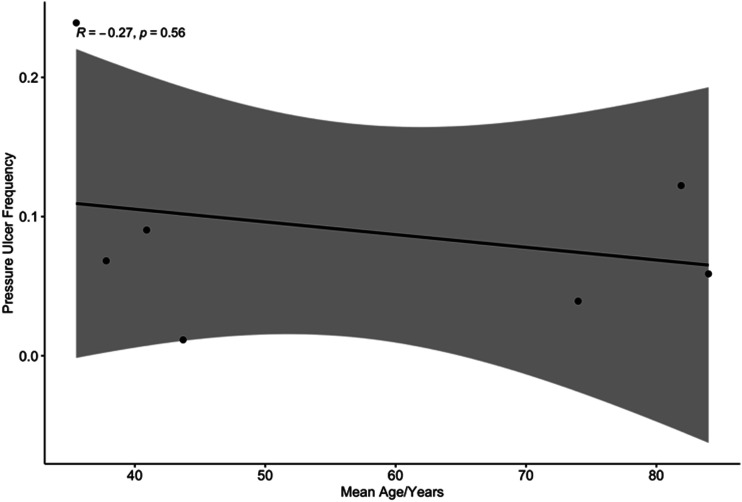
Bivariate regression analysis of pressure ulcer frequency as a function of mean patient age.

In order to explore potential sources of heterogeneity, factors associated with pressure ulceration were explored based on data reported. Seven studies reported on both mean patient age and the frequency of pressure ulceration in patients immobilised with hard collars. A bivariate regression analysis was undertaken to establish if there was a correlation between increased age and pressure ulceration. In the context of wide confidence intervals, there was no significant correlation observed between the variables (Figure 5; Spearman rank correlation coefficient, R, was −.27).

**Figure 6.**
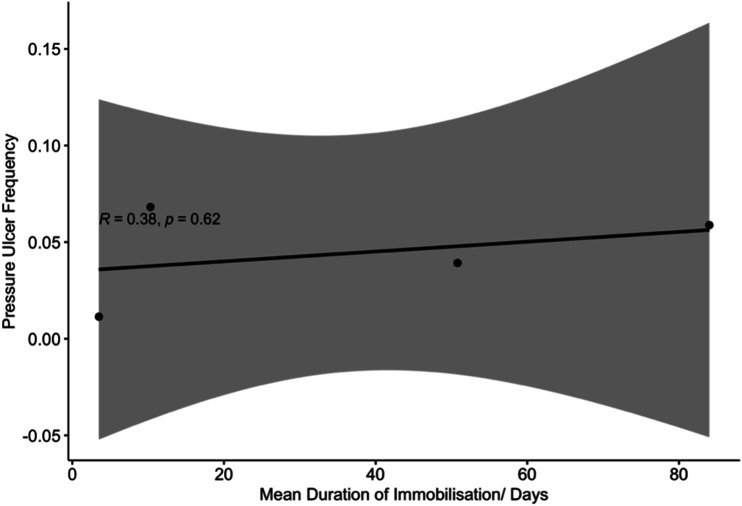
Bivariate regression analysis of pressure ulcer frequency as a function of mean duration of immobilisation.

A mean duration of immobilisation was disclosed in 4 studies reporting pressure ulcers.^7,20,22,24^ Bivariate regression analysis revealed no significant correlation between these variables (Figure 6; Spearman rank correlation coefficient, R, was .38). A positive association between length of stay and collar-related pressure ulceration was reported in 1 study.^25^

### Dysphagia

A total of 3 primary studies described dysphagia as a complication of prolonged use of a hard collar.^19,26,27^ Kalb et al. (2012)^26^ found no significant association compared to a group with no collar. However, Borders et al. (2018)^27^ reported that cervical bracing was both a significant predictor of dysphagia and associated with greater dysphagia severity. In a cohort study without a non-immobilised group, Moran et al. (2013)^19^ observed a prevalence of dysphagia of 50%.

### Increased Intracranial Pressure

A single randomised trial spanning 5 days^17^ demonstrated evidence of increased ICP each day, measured indirectly via optic nerve sheath diameter.

### Miscellaneous

The frequency of respiratory infections was assessed in 2 of the included studies.^19,25^ Lower respiratory tract infection (LRTI) was observed in 5/8 participants in a small cohort study,^19^ while hospital acquired pneumonia was diagnosed in 11/90 patients in a larger study.^25^ There was no control population without cervical immobilisation in either case. In both studies, all recruited patients were hospital inpatients aged over 65.

One study investigated the effects of hard collar immobilisation on postural control and voluntary eye movements in healthy volunteers.^18^ Measures of postural control were not significantly altered, however, there was a significant reduction in the velocity of voluntary saccades.

A single retrospective study with 24–48 month follow-up recorded a significant reduction in mean cervical range of motion following immobilisation for 8 weeks (46%), compared to 4 week immobilisation (25%).^5^

Psychosocial issues associated with prolonged hard collar use were examined in one study with outpatient follow up.^7^ Mood swings and/or low self-esteem were reported by 6/51 of the participants. The mean immobilisation duration was 7.25 weeks and there was again no control arm.

## Discussion

This is the first systematic review and meta-analysis studying adverse events associated with prolonged hard collar immobilisation. Current evidence is largely of low quality and based on inpatients with trauma. It is apparent that complications can occur. Pressure ulceration was the most frequently studied and suitable for meta-analysis, occurring in 7% (95% CI: 4%, 13%) of cases.

In trauma hard collars are used to immobilise the cervical spine in order to reduce pain and reduce the risk of displacement or deformity. Use of a hard collar is considered to be a safe therapy with low morbidity.

The findings of this review highlight the risk and incidence of complications. Hard collars double the occurrence of pressure sores above what is expected for inpatients in general^28^ and increase the risk of dysphagia.^27^ Moreover, there could be implications for co-existent injuries with one study using healthy volunteers showing that hard collars increase intracranial pressure.^17^ This corroborates an extensive literature of direct measurements.^29^ Management of raised intracranial pressure is a key component of the treatment of head injury, which co-exists with spinal trauma in up to 60% of cases.^30^

Due to the limited scope and length of observation, the incidence of complications is likely to be an under-estimate. Most of the reported evidence relates to inpatients following trauma with outpatient follow up only conducted in 4 studies.^7,19-21^ Much of the patient timeline is unaccounted for, omitting those patients with prolonged hard collar immobilisation. This leads to underreporting of complications. Constraints on the type of complication reported were imposed in each study. For instance, the two large cohort studies of dysphagia prevalence failed to report on pressure ulceration.^26,27^

Lastly, some complications cited in the literature were not discussed in the cohort studies, such as nerve compression palsies.^31^

How these findings apply to an elective and/or outpatient setting remains uncertain. The morbidity associated with immobilisation in a hard collar should be taken into account in decision making around the risks and benefits. The National Institute for Health Research, United Kingdom, has recently commissioned the DENS Trial. This study of the duration of external neck stabilisation following odontoid fracture in older or frail adults is a randomised controlled trial of collar vs no collar (DENS RCT).^32^ Whilst this population represents a subgroup of hard collar use, it will collect the first high-quality prospective data on hard collar morbidity.

One of the surprising findings of this review was that morbidity was not associated with age. In the context of study limitations, this should be interpreted cautiously, as skin fragility and therefore pressure sore risk increases with age.^33^ One potential explanation for this, would be the recognition of this risk, with planned interventions, such as selective use of hard collars and/or routine collar care for elderly patients, to mitigate it.

Another surprising finding was the absence of an association between length of stay and pressure ulceration. This is at odds with our existing understanding for the pathophysiology of pressure ulcers,^34^ whereby an increased time of immobilisation increases the risk of small ischaemic events of the skin. Again, this must therefore be interpreted cautiously, largely due to the limited reporting of duration and length of follow up (Figure 6).

### Limitations

The included studies had inconsistent methodologies, outcome measures and reporting style. An important limitation is the sparse and haphazard nature of the literature. This was offset, at least in part, by using a random effects model in the meta-analysis.

The identification of pressure ulcers is inconsistent, as evidenced by the lower inter-observer reliability using the European Pressure Ulcer Advisory Panel (EPUAP) classification system.^35^ This was minimised in the prospective cohort studies by experienced observers. More generally, distinguishing between EPUAP scores was not relevant to this meta-analysis and confusion with other lesions is unlikely in the cervical region.

Omissions in data limited the power of some analyses, such as the infrequent reporting of the duration of immobilisation and the absence of reporting the time at which complications occurred. Similarly, there was little data in middle-aged adults, illustrated in Figure 4. This was likely a consequence of the bimodal age distribution of cervical injuries in young adults and the elderly. Absent data was recognised both in the interpretation and graphically by confidence intervals.

Whilst Borders et al. (2018)^27^ established a positive dysphagia association with videofluoroscopic swallowing studies, Kalb et al. (2012)^26^ found no association in subjective clinician and patient reports. It can be argued that subjective dysphagia is more clinically relevant. Nonetheless, image-confirmed dysphagia suggests there might exist a greater risk of aspiration.

### Implications for Practice and Future Investigation

The rates of surgery for age-related (degenerative) indications are rising^36^ and an ageing population is at greater risk of low-velocity cervical spine fragility injuries.^37^ Hard collar immobilisation may be used increasingly in the future despite uncertainty about effectiveness and the incidence of adverse events*.*

Of the few examples we identified, both the collar design and co-existent care were implicated in modifying adverse events. For example, Spark et al. (2013)^38^ reported that Miami J and Aspen collars were more favourable for features such as reduced tissue interface pressure and reduced skin humidity, whilst Powers (1997)^39^ used a nursing protocol to reduce the occurrence of pressure sores.

Although mentioned in patient advice leaflets as a complication,^40^ this review did not capture any direct studies of cervical muscle atrophy consequent to hard collar use. Just one included study alludes to this,^5^ with subtle range of motion deficits measured following longer immobilisation. It is suggested that cervical muscle atrophy may be responsible. This represents a further knowledge gap in the literature.

A growing body of evidence suggests that hard collars are often used following cervical spine surgery. This comprises both the decision whether to use a hard collar and the duration of immobilisation. For some common procedures, neither clinical outcome nor fusion are different with or without the use of a hard collar.^5,8,9^

Evidence is needed to allow informed and cost-effective decision making between surgeons and patients. The shifting cost-benefit analysis lends itself to randomised controlled trial with a specific focus on hard collar complications. The aforementioned DENS trial is therefore a welcome start.

## Conclusion

Prolonged immobilisation with hard collars causes a range of morbidity, including pressure sores, dysphagia, increased ICP and peripheral nerve palsies. However, the current data reporting on incidence is of low quality, and high-quality prospective studies are needed to decide on the merits and risks of using hard collar in patients with cervical spine injuries and following cervical spine surgery.

## Supplementary Material

Supplementary material

Supplementary material

Supplementary material
