# Supplementary material for: Adverse Events Relating to Prolonged Hard Collar Immobilisation: A Systematic Review and Meta-Analysis
Source: Global Spine J. 2022 Mar 25;12(8):1968–78. doi: 10.1177/21925682221087194 (PMC9609519; doi:10.1177/21925682221087194)
Supplement: Supplementary material [file sj-pdf-2-gsj-10.1177_21925682221087194.pdf]

Embase <1974 to 2021 April 09>

1 (hard collar* or hard cervical collar* or hard neck collar*).mp. [mp=title, abstract, heading word, drug trade name, original title, device manufacturer, drug manufacturer, device trade name, keyword, floating subheading word, candidate term word] 190
2 (rigid collar* or rigid cervical collar* or rigid neck collar*).mp. [mp=title, abstract, heading word, drug trade name, original title, device manufacturer, drug manufacturer, device trade name, keyword, floating subheading word, candidate term word] 321
3 (rigid orthosis* or rigid cervical orthosis* or rigid neck orthosis*).mp. [mp=title, abstract, heading word, drug trade name, original title, device manufacturer, drug manufacturer, device trade name, keyword, floating subheading word, candidate term word] 68
4 orthotic device*.mp. 994
5 brace*.mp. 15759
6 exp rigid collar/ 87
7 exp orthotics/ 3890
8 exp spinal brace/ 728
9 1 or 2 or 3 or 4 or 5 or 6 or 7 or 8 20403
10 complicat*.mp. 3195759
11 adverse event*.mp. 338444
12 adverse effect*.mp. 260743
13 adverse outcome*.mp. 80827
14 exp complication/ 1244977
15 exp postoperative complication/ 703759
16 exp adverse event/ 660328
17 exp adverse outcome/ 53891
18 exp adverse device effect/ 52213
19 10 or 11 or 12 or 13 or 14 or 15 or 16 or 17 or 18 4391179
20 cervical spin*.mp. 70291
21 cervical vertebr*.mp. 10836
22 exp cervical spine/ 38567
23 exp cervical vertebra/ 4711
24 20 or 21 or 22 or 23 75193
25 9 and 19 and 24 449

https://ovidsp.ovid.com/athens/ovidweb.cgi?T=JS&NEWS=N&PAGE=main&SHAREDSEARCHID=3ZybPX4EhUNaESeiqahHMa2xFMbDyFhFrgVNaBRvIqkwNCvqZcGhI93P7pejij3w9

Ovid MEDLINE(R) and Epub Ahead of Print, In-Process, In-Data-Review & Other Non-Indexed Citations, Daily and Versions(R) <1946 to April 09, 2021>

1 (hard collar* or hard cervical collar* or hard neck collar*).mp. [mp=title, abstract, original title, name of substance word, subject heading word, floating sub-heading word, keyword heading word, organism supplementary concept word, protocol supplementary concept word, rare disease supplementary concept word, unique identifier, synonyms] 137
2 (rigid collar* or rigid cervical collar* or rigid neck collar*).mp. [mp=title, abstract, original title, name of substance word, subject heading word, floating sub-heading word, keyword heading word, organism supplementary concept word, protocol supplementary concept word, rare disease supplementary concept word, unique identifier, synonyms] 221
3 (rigid orthosis* or rigid cervical orthosis* or rigid neck orthosis*).mp. [mp=title, abstract, original title, name of substance word, subject heading word, floating sub-heading word, keyword heading word, organism supplementary concept word, protocol supplementary concept word, rare disease supplementary concept word, unique identifier, synonyms] 54
4 orthotic device*.mp. 6917
5 brace*.mp. 10567
6 exp orthotic devices/ 15356
7 exp braces/ 5631
8 1 or 2 or 3 or 4 or 5 or 6 or 7 20555
9 complicat*.mp. 3301678
10 adverse event*.mp. 175510
11 adverse effect*.mp. 1903140
12 adverse outcome*.mp. 31569
13 exp Postoperative Complications/ 562038
14 exp Long Term Adverse Effects/ 649
15 9 or 10 or 11 or 12 or 13 or 14 4875695
16 cervical spin*.mp. 31356
17 cervical vertebr*.mp. 40710
18 exp Cervical Vertebrae/ 40315
19 16 or 17 or 18 57454
20 8 and 15 and 19 512

https://ovidsp.ovid.com/athens/ovidweb.cgi?T=JS&NEWS=N&PAGE=main&SHAREDSEARCHID=3ZybPX4EhUNaESeiqahHMaPpRssFfOS0K0eaqxVG0TpAo6uTuZgWxNvSK61YkEI6M
