# Supplementary material for: Adverse Events Relating to Prolonged Hard Collar Immobilisation: A Systematic Review and Meta-Analysis
Source: Global Spine J. 2022 Mar 25;12(8):1968–78. doi: 10.1177/21925682221087194 (PMC9609519; doi:10.1177/21925682221087194)
Supplement: Supplementary material [file sj-pdf-3-gsj-10.1177_21925682221087194.pdf]

# JBI CRITICAL APPRAISAL CHECKLIST FOR STUDIES REPORTING PREVALENCE DATA

Reviewer _____Jamie Brannigan_____ Date _____03/05/2021_____

Author _____Ackland et al_____ Year __2007__ Record Number __9/26____

| | Yes | No | Unclear | Not applicable |
|---|---|---|---|---|
| 1. Was the sample frame appropriate to address the target population? | ☒ | ☐ | ☐ | ☐ |
| 2. Were study participants sampled in an appropriate way? | ☒ | ☐ | ☐ | ☐ |
| 3. Was the sample size adequate? | ☐ | ☐ | ☒ | ☐ |
| 4. Were the study subjects and the setting described in detail? | ☒ | ☐ | ☐ | ☐ |
| 5. Was the data analysis conducted with sufficient coverage of the identified sample? | ☒ | ☐ | ☐ | ☐ |
| 6. Were valid methods used for the identification of the condition? | ☐ | ☐ | ☒ | ☐ |
| 7. Was the condition measured in a standard, reliable way for all participants? | ☒ | ☐ | ☐ | ☐ |
| 8. Was there appropriate statistical analysis? | ☒ | ☐ | ☐ | ☐ |
| 9. Was the response rate adequate, and if not, was the low response rate managed appropriately? | ☐ | ☐ | ☐ | ☒ |

Overall appraisal: Include ☒ Exclude ☐ Seek further info ☐

Comments (Including reason for exclusion)

Pressure ulcer data is suitable for quantitative meta-analysis. Validity of identification unclear due to retrospective assessment of care records. Inclusion of trauma patients admitted over a 6month period is an appropriate sampling frame and sampling process.

# JBI CRITICAL APPRAISAL CHECKLIST FOR STUDIES REPORTING PREVALENCE DATA

*How to cite: Munn Z, Moola S, Lisy K, Riitano D, Tufanaru C. Methodological guidance for systematic reviews of observational epidemiological studies reporting prevalence and incidence data. Int J Evid Based Healthc. 2015;13(3):147–153.*

Answers: Yes, No, Unclear or Not/Applicable

1. **Was the sample frame appropriate to address the target population?**

   This question relies upon knowledge of the broader characteristics of the population of interest and the geographical area. If the study is of women with breast cancer, knowledge of at least the characteristics, demographics and medical history is needed. The term "target population" should not be taken to infer every individual from everywhere or with similar disease or exposure characteristics. Instead, give consideration to specific population characteristics in the study, including age range, gender, morbidities, medications, and other potentially influential factors. For example, a sample frame may not be appropriate to address the target population if a certain group has been used (such as those working for one organisation, or one profession) and the results then inferred to the target population (i.e. working adults). A sample frame may be appropriate when it includes almost all the members of the target population (i.e. a census, or a complete list of participants or complete registry data).

2. **Were study participants recruited in an appropriate way?**

   Studies may report random sampling from a population, and the methods section should report how sampling was performed. Random probabilistic sampling from a defined subset of the population (sample frame) should be employed in most cases, however, random probabilistic sampling is not needed when everyone in the sampling frame will be included/ analysed. For example, reporting on all the data from a good census is appropriate as a good census will identify everybody. When using cluster sampling, such as a random sample of villages within a region, the methods need to be clearly stated as the precision of the final prevalence estimate incorporates the clustering effect. Convenience samples, such as a street survey or interviewing lots of people at a public gatherings are not considered to provide a representative sample of the base population.

© JBI, 2020. All rights reserved. JBI grants use of these tools for research purposes only. All other enquiries should be sent to jbisynthesis@adelaide.edu.au.

3. **Was the sample size adequate?**

The larger the sample, the narrower will be the confidence interval around the prevalence estimate, making the results more precise. An adequate sample size is important to ensure good precision of the final estimate. Ideally we are looking for evidence that the authors conducted a sample size calculation to determine an adequate sample size. This will estimate how many subjects are needed to produce a reliable estimate of the measure(s) of interest. For conditions with a low prevalence, a larger sample size is needed. Also consider sample sizes for subgroup (or characteristics) analyses, and whether these are appropriate. Sometimes, the study will be large enough (as in large national surveys) whereby a sample size calculation is not required. In these cases, sample size can be considered adequate.

When there is no sample size calculation and it is not a large national survey, the reviewers may consider conducting their own sample size analysis using the following formula: (Naing et al. 2006, Daniel 1999)

n= Z2P(1-P)

d2

Where:

n= sample size

Z = Z statistic for a level of confidence

P = Expected prevalence or proportion (in proportion of one; if 20%, P = 0.2)

d = precision (in proportion of one; if 5%, d=0.05)

**Ref:**

Naing L, Winn T, Rusli BN. Practical issues in calculating the sample size for prevalence studies Archives of Orofacial Sciences. 2006;1:9-14.

Daniel WW. Biostatistics: A Foundation for Analysis in the Health Sciences.

Edition. 7th ed. New York: John Wiley & Sons. 1999.

© JBI, 2020. All rights reserved. JBI grants use of these tools for research purposes only. All other enquiries should be sent to jbisynthesis@adelaide.edu.au.

4. **Were the study subjects and setting described in detail?**

   Certain diseases or conditions vary in prevalence across different geographic regions and populations (e.g. Women vs. Men, sociodemographic variables between countries). The study sample should be described in sufficient detail so that other researchers can determine if it is comparable to the population of interest to them.

5. **Was data analysis conducted with sufficient coverage of the identified sample?**

   Coverage bias can occur when not all subgroups of the identified sample respond at the same rate. For instance, you may have a very high response rate overall for your study, but the response rate for a certain subgroup (i.e. older adults) may be quite low.

6. **Were valid methods used for the identification of the condition?**

   Here we are looking for measurement or classification bias. Many health problems are not easily diagnosed or defined and some measures may not be capable of including or excluding appropriate levels or stages of the health problem. If the outcomes were assessed based on existing definitions or diagnostic criteria, then the answer to this question is likely to be yes. If the outcomes were assessed using observer reported, or self-reported scales, the risk of over- or under-reporting is increased, and objectivity is compromised. Importantly, determine if the measurement tools used were validated instruments as this has a significant impact on outcome assessment validity.

7. **Was the condition measured in a standard, reliable way for all participants?**

   Considerable judgment is required to determine the presence of some health outcomes. Having established the validity of the outcome measurement instrument (see item 6 of this scale), it is important to establish how the measurement was conducted. Were those involved in collecting data trained or educated in the use of the instrument/s? If there was more than one data collector, were they similar in terms of level of education, clinical or research experience, or level of responsibility in the piece of research being appraised? When there was more than one observer or collector, was there comparison of results from across the observers? Was the condition measured in the same way for all participants?

© JBI, 2020. All rights reserved. JBI grants use of these tools for research purposes only. All other enquiries should be sent to jbisynthesis@adelaide.edu.au.

8. **Was there appropriate statistical analysis?**

Importantly, the numerator and denominator should be clearly reported, and percentages should be given with confidence intervals. The methods section should be detailed enough for reviewers to identify the analytical technique used and how specific variables were measured. Additionally, it is also important to assess the appropriateness of the analytical strategy in terms of the assumptions associated with the approach as differing methods of analysis are based on differing assumptions about the data and how it will respond.

9. **Was the response rate adequate, and if not, was the low response rate managed appropriately?**

A large number of dropouts, refusals or "not founds" amongst selected subjects may diminish a study's validity, as can a low response rates for survey studies. The authors should clearly discuss the response rate and any reasons for non-response and compare persons in the study to those not in the study, particularly with regards to their socio-demographic characteristics. If reasons for non-response appear to be unrelated to the outcome measured and the characteristics of non-responders are comparable to those who do respond in the study (addressed in question 5, coverage bias), the researchers may be able to justify a more modest response rate.

© JBI, 2020. All rights reserved. JBI grants use of these tools for research purposes only. All other enquiries should be sent to jbisynthesis@adelaide.edu.au.

# JBI CRITICAL APPRAISAL CHECKLIST FOR COHORT STUDIES

Reviewer_____Jamie Brannigan______________________Date_____03/05/2021____________

Author_________Borders et al________________________Year___2018___ Record Number__11/26___

| | Yes | No | Unclear | Not applicable |
|---|---|---|---|---|
| 1. Were the two groups similar and recruited from the same population? | ☒ | ☐ | ☐ | ☐ |
| 2. Were the exposures measured similarly to assign people<br>3. to both exposed and unexposed groups? | ☒ | ☐ | ☐ | ☐ |
| 4. Was the exposure measured in a valid and reliable way? | ☒ | ☐ | ☐ | ☐ |
| 5. Were confounding factors identified? | ☒ | ☐ | ☐ | ☐ |
| 6. Were strategies to deal with confounding factors stated? | ☒ | ☐ | ☐ | ☐ |
| 7. Were the groups/participants free of the outcome at the start of the study (or at the moment of exposure)? | ☒ | ☐ | ☐ | ☐ |
| 8. Were the outcomes measured in a valid and reliable way? | ☐ | ☐ | ☒ | ☐ |
| 9. Was the follow up time reported and sufficient to be long enough for outcomes to occur? | ☐ | ☐ | ☐ | ☒ |
| 10. Was follow up complete, and if not, were the reasons to loss to follow up described and explored? | ☐ | ☐ | ☐ | ☒ |
| 11. Were strategies to address incomplete follow up utilized? | ☐ | ☐ | ☐ | ☒ |
| 12. Was appropriate statistical analysis used? | ☒ | ☐ | ☐ | ☐ |

Overall appraisal: Include ☒ Exclude ☐ Seek further info ☐

Comments (Including reason for exclusion)

Retrospective nature means that follow up is non-applicable. Outcome measure validity is unclear as there is no universally accepted method for determining dysphagia.

© JBI, 2020. All rights reserved. JBI grants use of these

tools for research purposes only. All other enquiries should be sent to jbisynthesis@adelaide.edu.au.

# EXPLANATION OF COHORT STUDIES CRITICAL APPRAISAL

How to Cite: *Moola S, Munn Z, Tufanaru C, Aromataris E, Sears K, Sfetcu R, Currie M, Qureshi R, Mattis P, Lisy K, Mu P-F. Chapter 7: Systematic reviews of etiology and risk . In: Aromataris E, Munn Z (Editors). JBI Manual for Evidence Synthesis.* JBI, 2020. Available from https://synthesismanual.jbi.global

## Cohort Studies Critical Appraisal Tool

Answers: Yes, No, Unclear or Not/Applicable

### 1. Were the two groups similar and recruited from the same population?

Check the paper carefully for descriptions of participants to determine if patients within and across groups have similar characteristics in relation to exposure (e.g. risk factor under investigation). The two groups selected for comparison should be as similar as possible in all characteristics except for their exposure status, relevant to the study in question. The authors should provide clear inclusion and exclusion criteria that they developed prior to recruitment of the study participants.

### 2. Were the exposures measured similarly to assign people to both exposed and unexposed groups?

A high quality study at the level of cohort design should mention or describe how the exposures were measured. The exposure measures should be clearly defined and described in detail. This will enable reviewers to assess whether or not the participants received the exposure of interest.

### 3. Was the exposure measured in a valid and reliable way?

The study should clearly describe the method of measurement of exposure. Assessing validity requires that a 'gold standard' is available to which the measure can be compared. The validity of exposure measurement usually relates to whether a current measure is appropriate or whether a measure of past exposure is needed.

Reliability refers to the processes included in an epidemiological study to check repeatability of measurements of the exposures. These usually include intra-observer reliability and inter-observer reliability.

### 4. Were confounding factors identified?

Confounding has occurred where the estimated intervention exposure effect is biased by the presence of some difference between the comparison groups (apart from the exposure investigated/of interest). Typical confounders include baseline characteristics, prognostic factors, or concomitant exposures (e.g. smoking). A confounder is a difference between the comparison groups and it influences the direction of the study results. A high quality study at the level of cohort design will identify the potential confounders and measure them (where possible). This is difficult for studies where behavioral, attitudinal or lifestyle factors may impact on the results.

© JBI, 2020. All rights reserved. JBI grants use of these tools for research purposes only. All other enquiries should be sent to jbisynthesis@adelaide.edu.au.

**5. Were strategies to deal with confounding factors stated?**

Strategies to deal with effects of confounding factors may be dealt within the study design or in data analysis. By matching or stratifying sampling of participants, effects of confounding factors can be adjusted for. When dealing with adjustment in data analysis, assess the statistics used in the study. Most will be some form of multivariate regression analysis to account for the confounding factors measured. Look out for a description of statistical methods as regression methods such as logistic regression are usually employed to deal with confounding factors/variables of interest.

**6. Were the groups/participants free of the outcome at the start of the study (or at the moment of exposure)?**

The participants should be free of the outcomes of interest at the start of the study. Refer to the 'methods' section in the paper for this information, which is usually found in descriptions of participant/sample recruitment, definitions of variables, and/or inclusion/exclusion criteria.

**7. Were the outcomes measured in a valid and reliable way?**

Read the methods section of the paper. If for e.g. lung cancer is assessed based on existing definitions or diagnostic criteria, then the answer to this question is likely to be yes. If lung cancer is assessed using observer reported, or self-reported scales, the risk of over- or under-reporting is increased, and objectivity is compromised. Importantly, determine if the measurement tools used were validated instruments as this has a significant impact on outcome assessment validity.

Having established the objectivity of the outcome measurement (e.g. lung cancer) instrument, it's important to establish how the measurement was conducted. Were those involved in collecting data trained or educated in the use of the instrument/s? (e.g. radiographers). If there was more than one data collector, were they similar in terms of level of education, clinical or research experience, or level of responsibility in the piece of research being appraised?

**8. Was the follow up time reported and sufficient to be long enough for outcomes to occur?**

The appropriate length of time for follow up will vary with the nature and characteristics of the population of interest and/or the intervention, disease or exposure. To estimate an appropriate duration of follow up, read across multiple papers and take note of the range for duration of follow up. The opinions of experts in clinical practice or clinical research may also assist in determining an appropriate duration of follow up. For example, a longer timeframe may be needed to examine the association between occupational exposure to asbestos and the risk of lung cancer. It is important, particularly in cohort studies that follow up is long enough to enable the outcomes. However, it should be remembered that the research question and outcomes being examined would probably dictate the follow up time.

© JBI, 2020. All rights reserved. JBI grants use of these tools for research purposes only. All other enquiries should be sent to jbisynthesis@adelaide.edu.au.

## 9. Was follow up complete, and if not, were the reasons to loss to follow up described and explored?

It is important in a cohort study that a greater percentage of people are followed up. As a general guideline, at least 80% of patients should be followed up. Generally a dropout rate of 5% or less is considered insignificant. A rate of 20% or greater is considered to significantly impact on the validity of the study. However, in observational studies conducted over a lengthy period of time a higher dropout rate is to be expected. A decision on whether to include or exclude a study because of a high dropout rate is a matter of judgement based on the reasons why people dropped out, and whether dropout rates were comparable in the exposed and unexposed groups.

Reporting of efforts to follow up participants that dropped out may be regarded as an indicator of a well conducted study. Look for clear and justifiable description of why people were left out, excluded, dropped out etc. If there is no clear description or a statement in this regards, this will be a 'No'.

## 10. Were strategies to address incomplete follow up utilized?

Some people may withdraw due to change in employment or some may die; however, it is important that their outcomes are assessed. Selection bias may occur as a result of incomplete follow up. Therefore, participants with unequal follow up periods must be taken into account in the analysis, which should be adjusted to allow for differences in length of follow up periods. This is usually done by calculating rates which use person-years at risk, i.e. considering time in the denominator.

## 11. Was appropriate statistical analysis used?

As with any consideration of statistical analysis, consideration should be given to whether there was a more appropriate alternate statistical method that could have been used. The methods section of cohort studies should be detailed enough for reviewers to identify which analytical techniques were used (in particular, regression or stratification) and how specific confounders were measured.

For studies utilizing regression analysis, it is useful to identify if the study identified which variables were included and how they related to the outcome. If stratification was the analytical approach used, were the strata of analysis defined by the specified variables? Additionally, it is also important to assess the appropriateness of the analytical strategy in terms of the assumptions associated with the approach as differing methods of analysis are based on differing assumptions about the data and how it will respond.

© JBI, 2020. All rights reserved. JBI grants use of these tools for research purposes only. All other enquiries should be sent to jbisynthesis@adelaide.edu.au.

# JBI CRITICAL APPRAISAL CHECKLIST FOR SYSTEMATIC REVIEWS AND RESEARCH SYNTHESES

Reviewer _________Jamie Brannigan________________Date________03/05/2021_______________

Author_______Dunham et al______________________Year___2008___ Record Number_24/26____

| | Question | Yes | No | Unclear | Not applicable |
|---|---|---|---|---|---|
| 10. | Is the review question clearly and explicitly stated? | ☐ | ☒ | ☐ | ☐ |
| 11. | Were the inclusion criteria appropriate for the review question? | ☐ | ☐ | ☐ | ☒ |
| 12. | Was the search strategy appropriate? | ☒ | ☐ | ☐ | ☐ |
| 13. | Were the sources and resources used to search for studies adequate? | ☒ | ☐ | ☐ | ☐ |
| 14. | Were the criteria for appraising studies appropriate? | ☐ | ☐ | ☒ | ☐ |
| 15. | Was critical appraisal conducted by two or more reviewers independently? | ☐ | ☐ | ☒ | ☐ |
| 16. | Were there methods to minimize errors in data extraction? | ☐ | ☐ | ☒ | ☐ |
| 17. | Were the methods used to combine studies appropriate? | ☐ | ☐ | ☐ | ☒ |
| 18. | Was the likelihood of publication bias assessed? | ☐ | ☐ | ☒ | ☐ |
| 19. | Were recommendations for policy and/or practice supported by the reported data? | ☒ | ☐ | ☐ | ☐ |
| 20. | Were the specific directives for new research appropriate? | ☐ | ☐ | ☐ | ☒ |

Overall appraisal: Include ☒ Exclude ☐ Seek further info ☐

Comments (Including reason for exclusion)

This was a simple literature review, rather than a systematic review. There were several research questions addressed in subsections with loosely systematic searches. Studies appear not to have been appraised thoroughly and it is not understood if more than one reviewer was involved in screening and data extraction. Inclusion is only appropriate for the narrative component of the review.

# JBI CRITICAL APPRAISAL CHECKLIST FOR SYSTEMATIC REVIEWS AND RESEARCH SYNTHESIS

How to cite: *Aromataris E, Fernandez R, Godfrey C, Holly C, Kahlil H, Tungpunkom P. Summarizing systematic reviews: methodological development, conduct and reporting of an Umbrella review approach. Int J Evid Based Healthc. 2015;13(3):132-40.*

When conducting an umbrella review using the JBI method, the critical appraisal instrument for Systematic Reviews should be used.

The primary and secondary reviewer should discuss each item in the appraisal instrument for each study included in their review. In particular, discussions should focus on what is considered acceptable to the aims of the review in terms of the specific study characteristics. When appraising systematic reviews this discussion may include issues such as what represents an adequate search strategy or appropriate methods of synthesis. The reviewers should be clear on what constitutes acceptable levels of information to allocate a positive appraisal compared with a negative, or response of “unclear”. This discussion should ideally take place before the reviewers independently conduct the appraisal.

Within umbrella reviews, quantitative or qualitative systematic reviews may be incorporated, as well as meta-analyses of existing research. There are 11 questions to guide the appraisal of systematic reviews or meta-analyses. Each question should be answered as “yes”, “no”, or “unclear”. Not applicable “NA” is also provided as an option and may be appropriate in rare instances.

### 1. Is the review question clearly and explicitly stated?

The review question is an essential step in the systematic review process. A well-articulated question defines the scope of the review and aids in the development of the search strategy to locate the relevant evidence. An explicitly stated question, formulated around its PICO (Population, Intervention, Comparator, Outcome) elements aids both the review team in the conduct of the review and the reader in determining if the review has achieved its objectives. Ideally the review question should be articulated in a published protocol; however this will not always be the case with many reviews that are located.

### 2. Were the inclusion criteria appropriate for the review question?

The inclusion criteria should be identifiable from, and match the review question. The necessary elements of the PICO should be explicit and clearly defined. The inclusion criteria should be detailed and the included reviews should clearly be eligible when matched against the stated inclusion criteria. Appraisers of meta-analyses will find that inclusion criteria may include criteria around the ability to conduct statistical analyses which would not be the norm for a systematic review. The types of included studies should be relevant to the review question, for example, an umbrella review aiming to summarize a range of effective non-pharmacological interventions for aggressive behaviors amongst elderly patients with dementia will limit itself to including systematic reviews and meta-analyses that synthesize quantitative studies assessing the various interventions; qualitative or economic reviews would not be included.

### 3. Was the search strategy appropriate?

A systematic review should provide evidence of the search strategy that has been used to locate the evidence. This may be found in the methods section of the review report in some cases, or as an appendix that may be provided as supplementary information to the review publication. A systematic review should present a clear search strategy that addresses each of the identifiable PICO components of the review question. Some reviews may also provide a description of the approach to searching and how the terms that were ultimately used were derived, though due to limits on word counts in journals this may be more the norm in online only publications. There

© JBI, 2020. All rights reserved. JBI grants use of these tools for research purposes only. All other enquiries should be sent to jbisynthesis@adelaide.edu.au.

should be evidence of logical and relevant keywords and terms and also evidence that Subject

Headings and Indexing terms have been used in the conduct of the search. Limits on the search should also be considered and their potential impact; for example, if a date limit was used, was this appropriate and/or justified? If only English language studies were included, will such a language bias have an impact on the review? The response to these considerations will depend, in part, on the review question.

## 4. Were the sources and resources used to search for studies adequate?

A systematic review should attempt to identify "all" the available evidence and as such there should be evidence of a comprehensive search strategy. Multiple electronic databases should be searched including major bibliographic citation databases such as MEDLINE and CINAHL. Ideally, other databases that are relevant to the review question should also be searched, for example, a systematic review with a question about a physical therapy intervention should also look to search the PEDro database, whilst a review focusing on an educational intervention should also search the ERIC. Reviews of effectiveness should aim to search trial registries. A comprehensive search is the ideal way to minimize publication bias, as a result, a well conducted systematic review should also attempt to search for grey literature, or "unpublished" studies; this may involve searching websites relevant to the review question, or thesis repositories.

## 5. Were the criteria for appraising studies appropriate?

The systematic review should present a clear statement that critical appraisal was conducted and provide the details of the items that were used to assess the included studies. This may be presented in the methods of the review, as an appendix of supplementary information, or as a reference to a source that can be located. The tools or instruments used should be appropriate for the review question asked and the type of research conducted. For example, a systematic review of effectiveness should present a tool or instrument that addresses aspects of validity for experimental studies and randomized controlled trials such as randomization and blinding – if the review includes observational research to answer the same question a different tool would be more appropriate. Similarly, a review assessing diagnostic test accuracy may refer to the recognized QUADAS[1] tool.

## 6. Was critical appraisal conducted by two or more reviewers independently?

Critical appraisal or some similar assessment of the quality of the literature included in a systematic review is essential. A key characteristic to minimize bias or systematic error in the conduct of a systematic review is to have the critical appraisal of the included studies completed independently and in duplicate by members of the review team. The systematic review should present a clear statement that critical appraisal was conducted by at least two reviewers working independently from each other and conferring where necessary to reach decision regarding study quality and eligibility on the basis of quality.

## 7. Were there methods to minimize errors in data extraction?

Efforts made by review authors during data extraction can also minimize bias or systematic errors in the conduct of a systematic review. Strategies to minimize bias may include conducting all data extraction in duplicate and independently, using specific tools or instruments to guide data extraction and some evidence of piloting or training around their use.

## 8. Were the methods used to combine studies appropriate?

A synthesis of the evidence is a key feature of a systematic review. The synthesis that is presented should be appropriate for the review question and the stated type of systematic review and evidence it refers to. If a meta-analysis has been conducted this needs to be reviewed carefully.

© JBI, 2020. All rights reserved. JBI grants use of these tools for research purposes only. All other enquiries should be sent to jbisynthesis@adelaide.edu.au.

Was it appropriate to combine the studies? Have the reviewers assessed heterogeneity statistically and provided some explanation for heterogeneity that may be present? Often, where heterogeneous studies are included in the systematic review, narrative synthesis will be an appropriate method for presenting the results of multiple studies. If a qualitative review, are the methods that have been used to synthesize findings congruent with the stated methodology of the review? Is there adequate descriptive and explanatory information to support the final synthesized findings that have been constructed from the findings sourced from the original research?

### 9. Was the likelihood of publication bias assessed?

As mentioned, a comprehensive search strategy is the best means by which a review author may alleviate the impact of publication bias on the results of the review. Reviews may also present statistical tests such as Egger's test or funnel plots to also assess the potential presence of publication bias and its potential impact on the results of the review. This question will not be applicable to systematic reviews of qualitative evidence.

### 10. Were recommendations for policy and/or practice supported by the reported data?

Whilst the first nine (9) questions specifically look to identify potential bias in the conduct of a systematic review, the final questions are more indictors of review quality rather than validity. Ideally a review should present recommendations for policy and practice. Where these recommendations are made there should be a clear link to the results of the review. Is there evidence that the strength of the findings and the quality of the research been considered in the formulation of review recommendations?

### 11. Were the specific directives for new research appropriate?

The systematic review process is recognized for its ability to identify where gaps in the research, or knowledge base, around a particular topic exist. Most systematic review authors will provide some indication, often in the discussion section of the report, of where future research direction should lie. Where evidence is scarce or sample sizes that support overall estimates of effect are small and effect estimates are imprecise, repeating similar research to those identified by the review may be necessary and appropriate. In other instances, the case for new research questions to investigate the topic may be warranted.

## REFERENCES

1. Whiting P, Rutjes AWS, Reitsma JB, Bossuyt PMM, Kleijnen J. The development of QUADAS: a tool for the quality assessment of studies of diagnostic accuracy included in systematic reviews. BMC Medical Research Methodology. 2003;3:25 doi:10.1186/1471-2288-3-25.

# JBI CRITICAL APPRAISAL CHECKLIST FOR ECONOMIC EVALUATIONS

© JBI, 2020. All rights reserved. JBI grants use of these tools for research purposes only. All other enquiries should be sent to jbisynthesis@adelaide.edu.au.

Reviewer ___________Jamie__________________________________________Brannigan_______________
Date_________03/05/2021______________________

Author_______Ertel et al_____________Year___2016______ Record Number___25/26____

| | Yes | No | Unclear | Not applicable |
|---|---|---|---|---|
| 21. Is there a well-defined question? | ☒ | ☐ | ☐ | ☐ |
| 22. Is there comprehensive description of alternatives? | ☒ | ☐ | ☐ | ☐ |
| 23. Are all important and relevant costs and outcomes for each alternative identified? | ☒ | ☐ | ☐ | ☐ |
| 24. Has clinical effectiveness been established? | ☒ | ☐ | ☐ | ☐ |
| 25. Are costs and outcomes measured accurately? | ☒ | ☐ | ☐ | ☐ |
| 26. Are costs and outcomes valued credibly? | ☐ | ☐ | ☒ | ☐ |
| 27. Are costs and outcomes adjusted for differential timing? | ☒ | ☐ | ☐ | ☐ |
| 28. Is there an incremental analysis of costs and consequences? | ☒ | ☐ | ☐ | ☐ |
| 29. Were sensitivity analyses conducted to investigate uncertainty in estimates of cost or consequences? | ☒ | ☐ | ☐ | ☐ |
| 30. Do study results include all issues of concern to users? | ☐ | ☒ | ☐ | ☐ |
| 31. Are the results generalizable to the setting of interest in the review? | ☒ | ☐ | ☐ | ☐ |

Overall appraisal: Include ☒ Exclude ☐ Seek further info ☐

Comments (Including reason for exclusion)

Study results do not consider the full range of complications related to prolonged C-spine immobilisation. Costs and outcomes credibility rests on the work of cited primary research. Hence a definitive assessment cannot be made reading this paper.

© JBI, 2020. All rights reserved. JBI grants use of these tools for research purposes only. All other enquiries should be sent to jbisynthesis@adelaide.edu.au.

# JBI CRITICAL APPRAISAL CHECKLIST FOR ECONOMIC EVALUATIONS

*How to cite: Gomersall JS, Jadotte YT, Xue Y, Lockwood S, Riddle D, Preda A. Conducting systematic reviews of economic evaluations. Int J Evid Based Healthc. 2015;13(3):170–178.*

This tool is informed by the work of Drummond et al, Methods for the economic evaluation of health care programmes. 2nd Edition. Oxford: Oxford Medical Publications, 1997.

1. **Is there a well-defined question/objective?**

   Consider the following before marking the study as compliant with this quality criterion:

   - Is the objective/question of the study clearly stated?
   - Does the statement reflect the perspective (e.g. patient or community or societal or health provider) used in measurement of costs or/and cost effectiveness?
   - Was the study placed in a particular decision making context?

2. **Is there a comprehensive description of alternatives?**

   To be marked as compliant with this criterion the authors of the study should offer a clear description of the intervention or interventions considered in the economic evaluation and the comparator or comparators. Compliance does not require that a broad range of interventions and comparators was considered. What is important here is clear description of the nature of the intervention and comparator whose cost/effeteness was measured.

3. **Are all important and relevant costs and outcomes for each alternative identified?**

   This quality criterion assesses the comprehensiveness and relevant of the cost and cost effectiveness outcomes measured in the economic evaluation. When deciding whether all important costs and outcomes have been identified/measured in the study reflect on whether the outcomes are sufficient in light of the objectives of the study. It is appropriate for a study that has the objective of measuring a narrow range of costs and benefits to identify and measure a limited range. However, the limits of the narrow approach should be drawn out in the study. It is not appropriate for a study which implies in its objective statement that it measures a broad range of costs for a broad range out outcomes to include only a very limited range of relevant costs and outcomes.

4. **Has clinical effectiveness been established?**

   To assess compliance with this quality criterion requires considering whether the study has reported the evidence used to derive the effectiveness estimate and the level of this evidence. If it is not clear how the effectiveness estimate was derived, the study cannot be marked as compliant. To achieve compliance for this criterion the effectiveness estimate in the evaluation does not need to be derived from the same study as the resource use/cost estimate. What is important is the there is a solid evidence base under-pinning the assumptions about the direction and magnitude of the effectiveness measure(s) used in the evaluation.

5. **Are costs and outcomes measured accurately?**

   This quality criterion assesses whether the study has used appropriate/best practice measurement method to measure costs and effectiveness. To decide whether a study should be marked as compliant consider whether the methods section of the paper offers a detail description of the measures used for costs and outcomes and how it justifies them. In addition, consider whether the authors/study implementers discussed any limitations associated with the measures used and concerns about the accuracy of measurement. In economic evaluations it is often difficult to

© JBI, 2020. All rights reserved. JBI grants use of these tools for research purposes only. All other enquiries should be sent to jbisynthesis@adelaide.edu.au.

measure costs and outcomes accurately, and hence in many cases this quality criterion will be difficult to achieve.

## 6. Are costs and outcomes valued credibly?

This quality criterion assesses whether appropriate prices were used to value costs and the validity of the valuation of benefits. It requires considering the method description and judging where there is a sufficient explanation about how costs and outcomes were valued and whether the justification for it is persuasive.

## 7. Are costs and outcomes adjusted for differential timing?

To be marked compliant for this question the study should have identified and justified the discount rate used. The time frame over which the study was conducted should also have been identified and justified.

## 8. Is there any incremental analysis of costs and consequences?

To achieve compliance the paper should report a measure that shows the change in costs and benefits for the intervention and comparator for a marginal shift in resources from the comparator to the intervention.

## 9. Were sensitivity analysis conducted to investigate uncertainty in estimates of costs or outcomes?

Sensitivity analysis is critical for establishing the validity of any economic evaluations results. To be compliant a study must present sensitivity testing results that describe how the study findings vary with changes in key variables (for example relative prices, and intervention estimates? conducted to check the robustness of findings.

## 10. Do study results include all issues of concern to users?

This question reflects on the comprehensiveness of coverage in the reporting of results. In deciding whether to mark the study as compliance consider whether the range of measures presented provider answers to all the questions users/decision makers would want to know when taking a decision about whether to implement the program examined (or cutting it)?

## 11. Are the results generalizable to the setting of interest in the review?

To be marked as compliant for this last quality criterion the paper should: (i) have described the study setting adequately; (ii) discuss the issue of transferability of findings and how the results are generalizable to other settings with similar characteristics

© JBI, 2020. All rights reserved. JBI grants use of these tools for research purposes only. All other enquiries should be sent to jbisynthesis@adelaide.edu.au.

# JBI CRITICAL APPRAISAL CHECKLIST FOR STUDIES REPORTING PREVALENCE DATA

Reviewer _____Jamie Brannigan_____ Date _____03/05/2021_____

Author_____Ham et al_____ Year__2014___ Record Number__7/26____

| | Yes | No | Unclear | Not applicable |
|---|---|---|---|---|
| Was the sample frame appropriate to address the target population? 32. | ☐ | ☐ | X | ☐ |
| 33. Were study participants sampled in an appropriate way? | X | ☐ | ☐ | ☐ |
| 34. Was the sample size adequate? | ☐ | ☐ | X | ☐ |
| 35. Were the study subjects and the setting described in detail? | X | ☐ | ☐ | ☐ |
| 36. Was the data analysis conducted with sufficient coverage of the identified sample? | X | ☐ | ☐ | ☐ |
| 37. Were valid methods used for the identification of the condition? | ☐ | ☐ | X | ☐ |
| Was the condition measured in a standard, reliable way for all participants? 38. | X | ☐ | ☐ | ☐ |
| 39. Was there appropriate statistical analysis? | X | ☐ | ☐ | ☐ |
| 40. Was the response rate adequate, and if not, was the low response rate managed appropriately? | ☐ | ☐ | ☐ | X |

Overall appraisal: Include X Exclude ☐ Seek further info ☐

Comments (Including reason for exclusion)

Pressure ulcer data is suitable for quantitative meta-analysis. Appropriateness of sample frame is unclear as patients were exclusively recruited in surgical intensive care unit. Validity of identification unclear due to retrospective assessment of care records.

# JBI CRITICAL APPRAISAL CHECKLIST FOR STUDIES REPORTING PREVALENCE DATA

*How to cite: Munn Z, Moola S, Lisy K, Riitano D, Tufanaru C. Methodological guidance for systematic reviews of observational epidemiological studies reporting prevalence and incidence data. Int J Evid Based Healthc. 2015;13(3):147–153.*

Answers: Yes, No, Unclear or Not/Applicable

**10. Was the sample frame appropriate to address the target population?**

This question relies upon knowledge of the broader characteristics of the population of interest and the geographical area. If the study is of women with breast cancer, knowledge of at least the characteristics, demographics and medical history is needed. The term "target population" should not be taken to infer every individual from everywhere or with similar disease or exposure characteristics. Instead, give consideration to specific population characteristics in the study, including age range, gender, morbidities, medications, and other potentially influential factors. For example, a sample frame may not be appropriate to address the target population if a certain group has been used (such as those working for one organisation, or one profession) and the results then inferred to the target population (i.e. working adults). A sample frame may be appropriate when it includes almost all the members of the target population (i.e. a census, or a complete list of participants or complete registry data).

**11. Were study participants recruited in an appropriate way?**

Studies may report random sampling from a population, and the methods section should report how sampling was performed. Random probabilistic sampling from a defined subset of the population (sample frame) should be employed in most cases, however, random probabilistic sampling is not needed when everyone in the sampling frame will be included/ analysed. For example, reporting on all the data from a good census is appropriate as a good census will identify everybody. When using cluster sampling, such as a random sample of villages within a region, the methods need to be clearly stated as the precision of the final prevalence estimate incorporates the clustering effect. Convenience samples, such as a street survey or interviewing lots of people at a public gatherings are not considered to provide a representative sample of the base population.

© JBI, 2020. All rights reserved. JBI grants use of these tools for research purposes only. All other enquiries should be sent to jbisynthesis@adelaide.edu.au.

## 12. Was the sample size adequate?

The larger the sample, the narrower will be the confidence interval around the prevalence estimate, making the results more precise. An adequate sample size is important to ensure good precision of the final estimate. Ideally we are looking for evidence that the authors conducted a sample size calculation to determine an adequate sample size. This will estimate how many subjects are needed to produce a reliable estimate of the measure(s) of interest. For conditions with a low prevalence, a larger sample size is needed. Also consider sample sizes for subgroup (or characteristics) analyses, and whether these are appropriate. Sometimes, the study will be large enough (as in large national surveys) whereby a sample size calculation is not required. In these cases, sample size can be considered adequate.

When there is no sample size calculation and it is not a large national survey, the reviewers may consider conducting their own sample size analysis using the following formula: (Naing et al. 2006, Daniel 1999)

n= Z2P(1-P)

d2

Where:

n= sample size

Z = Z statistic for a level of confidence

P = Expected prevalence or proportion (in proportion of one; if 20%, P = 0.2)

d = precision (in proportion of one; if 5%, d=0.05)

**Ref:**

Naing L, Winn T, Rusli BN. Practical issues in calculating the sample size for prevalence studies Archives of Orofacial Sciences. 2006;1:9-14.

Daniel WW. Biostatistics: A Foundation for Analysis in the Health Sciences.

Edition. 7th ed. New York: John Wiley & Sons. 1999.

© JBI, 2020. All rights reserved. JBI grants use of these tools for research purposes only. All other enquiries should be sent to jbisynthesis@adelaide.edu.au.

13. **Were the study subjects and setting described in detail?**

Certain diseases or conditions vary in prevalence across different geographic regions and populations (e.g.  Women vs. Men, sociodemographic variables between countries).  The study sample should be described in sufficient detail so that other researchers can determine if it is comparable to the population of interest to them.

14. **Was data analysis conducted with sufficient coverage of the identified sample?**

Coverage bias can occur when not all subgroups of the identified sample respond at the same rate. For instance, you may have a very high response rate overall for your study, but the response rate for a certain subgroup (i.e. older adults) may be quite low.

15. **Were valid methods used for the identification of the condition?**

Here we are looking for measurement or classification bias.  Many health problems are not easily diagnosed or defined and some measures may not be capable of including or excluding appropriate levels or stages of the health problem. If the outcomes were assessed based on existing definitions or diagnostic criteria, then the answer to this question is likely to be yes. If the outcomes were assessed using observer reported, or self-reported scales, the risk of over- or under-reporting is increased, and objectivity is compromised. Importantly, determine if the measurement tools used were validated instruments as this has a significant impact on outcome assessment validity.

16. **Was the condition measured in a standard, reliable way for all participants?**

Considerable judgment is required to determine the presence of some health outcomes. Having established the validity of the outcome measurement instrument (see item 6 of this scale), it is important to establish how the measurement was conducted.  Were those involved in collecting data trained or educated in the use of the instrument/s? If there was more than one data collector, were they similar in terms of level of education, clinical or research experience, or level of responsibility in the piece of research being appraised? When there was more than one observer or collector, was there comparison of results from across the observers? Was the condition measured in the same way for all participants?

© JBI, 2020. All rights reserved. JBI grants use of these tools for research purposes only. All other enquiries should be sent to jbisynthesis@adelaide.edu.au.

**17. Was there appropriate statistical analysis?**

Importantly, the numerator and denominator should be clearly reported, and percentages should be given with confidence intervals. The methods section should be detailed enough for reviewers to identify the analytical technique used and how specific variables were measured. Additionally, it is also important to assess the appropriateness of the analytical strategy in terms of the assumptions associated with the approach as differing methods of analysis are based on differing assumptions about the data and how it will respond.

**18. Was the response rate adequate, and if not, was the low response rate managed appropriately?**

A large number of dropouts, refusals or "not founds" amongst selected subjects may diminish a study's validity, as can a low response rates for survey studies. The authors should clearly discuss the response rate and any reasons for non-response and compare persons in the study to those not in the study, particularly with regards to their socio-demographic characteristics. If reasons for non-response appear to be unrelated to the outcome measured and the characteristics of non-responders are comparable to those who do respond in the study (addressed in question 5, coverage bias), the researchers may be able to justify a more modest response rate.

© JBI, 2020. All rights reserved. JBI grants use of these tools for research purposes only. All other enquiries should be sent to jbisynthesis@adelaide.edu.au.

# JBI CRITICAL APPRAISAL CHECKLIST FOR CASE REPORTS

Reviewer _____Jamie Brannigan_____________________________ Date______03/05/2021_______________

Author________Hewitt______________Year__1994___ Record Number___14/26______

| | Yes | No | Unclear | Not applicable |
|---|---|---|---|---|
| 41. Were patient's demographic characteristics clearly described? | ☐ | ☐ | ☒ | ☐ |
| 42. Was the patient's history clearly described and presented as a timeline? | ☒ | ☐ | ☐ | ☐ |
| 43. Was the current clinical condition of the patient on presentation clearly described? | ☒ | ☐ | ☐ | ☐ |
| 44. Were diagnostic tests or assessment methods and the results clearly described? | ☒ | ☐ | ☐ | ☐ |
| 45. Was the intervention(s) or treatment procedure(s) clearly described? | ☐ | ☒ | ☐ | ☐ |
| 46. Was the post-intervention clinical condition clearly described? | ☐ | ☒ | ☐ | ☐ |
| 47. Were adverse events (harms) or unanticipated events identified and described? | ☒ | ☐ | ☐ | ☐ |
| 48. Does the case report provide takeaway lessons? | ☒ | ☐ | ☐ | ☐ |

Overall appraisal: Include ☒ Exclude ☐ Seek further info ☐

Comments (Including reason for exclusion)

Age, gender and the existing surgical problem was discussed. Any further elaboration is not necessarily expected in adolescent trauma, however some co-morbidities may predispose to the complication of skin necrosis. There were few details of the surgical intervention. Case report finishes with a description of the compilation and no discussion of resolution/outcome.

# EXPLANATION OF CASE REPORTS CRITICAL APPRAISAL

*How to cite: Moola S, Munn Z, Tufanaru C, Aromataris E, Sears K, Sfetcu R, Currie M, Qureshi R, Mattis P, Lisy K, Mu P-F. Chapter 7: Systematic reviews of etiology and risk. In: Aromataris E, Munn Z (Editors). JBI Manual for Evidence Synthesis.* JBI, 2020. Available from https://synthesismanual.jbi.global

**Case Reports Critical Appraisal Tool**

Answers: Yes, No, Unclear or Not/Applicable

1. **Were patient's demographic characteristics clearly described?**

   Does the case report clearly describe patient's age, sex, race, medical history, diagnosis, prognosis, previous treatments, past and current diagnostic test results, and medications? The setting and context may also be described.

2. **Was the patient's history clearly described and presented as a timeline?**

   A good case report will clearly describe the history of the patient, their medical, family and psychosocial history including relevant genetic information, as well as relevant past interventions and their outcomes. (CARE Checklist 2013)

3. **Was the current clinical condition of the patient on presentation clearly described?**

   The current clinical condition of the patient should be described in detail including the uniqueness of the condition/disease, symptoms, frequency and severity. The case report should also be able to present whether differential diagnoses was considered.

4. **Were diagnostic tests or methods and the results clearly described?**

   A reader of the case report should be provided sufficient information to understand how the patient was assessed. It is important that all appropriate tests are ordered to confirm a diagnosis and therefore the case report should provide a clear description of various diagnostic tests used (whether a gold standard or alternative diagnostic tests). Photographs or illustrations of diagnostic procedures, radiographs, or treatment procedures are usually presented when appropriate to convey a clear message to readers.

5. **Was the intervention(s) or treatment procedure(s) clearly described?**

   It is important to clearly describe treatment or intervention procedures as other clinicians will be reading the paper and therefore may enable clear understanding of the treatment protocol. The report should describe the treatment/intervention protocol in detail; for e.g. in pharmacological management of dental anxiety - the type of drug, route of administration, drug dosage and frequency, and any side effects.

6. **Was the post-intervention clinical condition clearly described?**

   A good case report should clearly describe the clinical condition post-intervention in terms of the presence or lack thereof symptoms. The outcomes of management/treatment when presented as images or figures would help in conveying the information to the reader/clinician.

7. **Were adverse events (harms) or unanticipated events identified and described?**

   With any treatment/intervention/drug, there are bound to be some adverse events and in some cases, they may be severe. It is important that adverse events are clearly documented and described, particularly when a new or unique condition is being treated or when a new drug or treatment is used. In addition, unanticipated events, if any that may yield new or useful information should be identified and clearly described.

© JBI, 2020. All rights reserved. JBI grants use of these tools for research purposes only. All other enquiries should be sent to jbisynthesis@adelaide.edu.au.

8. **Does the case report provide takeaway lessons?**

   Case reports should summarize key lessons learned from a case in terms of the background of the condition/disease and clinical practice guidance for clinicians when presented with similar cases.

# REFERENCES:

Gagnier JJ, Kienle G, Altman DG, Moher D, Sox H, Riley D, CARE Group. The CARE Guidelines: Consensus-Based Clinical Case Reporting Guideline Development. Headache: The Journal of Head and Face Pain, 2013;53(10):1541-1547.

© JBI, 2020. All rights reserved. JBI grants use of these tools for research purposes only. All other enquiries should be sent to jbisynthesis@adelaide.edu.au.

# JBI CRITICAL APPRAISAL CHECKLIST FOR STUDIES REPORTING PREVALENCE DATA

Reviewer _____________Jamie Brannigan______________Date______03/05/2021________________

Author___________Hytlon et al________________Year__2016___ Record Number__6/26____

| | Yes | No | Unclear | Not applicable |
|---|---|---|---|---|
| Was the sample frame appropriate to address the target population? 49. | ☒ | ☐ | ☐ | ☐ |
| 50. Were study participants sampled in an appropriate way? | ☒ | ☐ | ☐ | ☐ |
| 51. Was the sample size adequate? | ☐ | ☐ | ☒ | ☐ |
| 52. Were the study subjects and the setting described in detail? | ☒ | ☐ | ☐ | ☐ |
| 53. Was the data analysis conducted with sufficient coverage of the identified sample? | ☒ | ☐ | ☐ | ☐ |
| 54. Were valid methods used for the identification of the condition? | ☒ | ☐ | ☐ | ☐ |
| Was the condition measured in a standard, reliable way for all participants? 55. | ☒ | ☐ | ☐ | ☐ |
| 56. Was there appropriate statistical analysis? | ☐ | ☒ | ☐ | ☐ |
| 57. Was the response rate adequate, and if not, was the low response rate managed appropriately? | ☐ | ☐ | ☐ | ☒ |

Overall appraisal: Include ☒ Exclude ☐ Seek further info ☐

Comments (Including reason for exclusion)

Pressure ulcer data is suitable for quantitative meta-analysis. Regarding statistical analysis, no explicit statement of numerator/denominator when outlining results.

# JBI CRITICAL APPRAISAL CHECKLIST FOR STUDIES REPORTING PREVALENCE DATA

*How to cite: Munn Z, Moola S, Lisy K, Riitano D, Tufanaru C. Methodological guidance for systematic reviews of observational epidemiological studies reporting prevalence and incidence data. Int J Evid Based Healthc. 2015;13(3):147–153.*

Answers: Yes, No, Unclear or Not/Applicable

**19. Was the sample frame appropriate to address the target population?**

This question relies upon knowledge of the broader characteristics of the population of interest and the geographical area. If the study is of women with breast cancer, knowledge of at least the characteristics, demographics and medical history is needed. The term "target population" should not be taken to infer every individual from everywhere or with similar disease or exposure characteristics. Instead, give consideration to specific population characteristics in the study, including age range, gender, morbidities, medications, and other potentially influential factors. For example, a sample frame may not be appropriate to address the target population if a certain group has been used (such as those working for one organisation, or one profession) and the results then inferred to the target population (i.e. working adults).  A sample frame may be appropriate when it includes almost all the members of the target population (i.e. a census, or a complete list of participants or complete registry data).

**20. Were study participants recruited in an appropriate way?**

Studies may report random sampling from a population, and the methods section should report how sampling was performed. Random probabilistic sampling from a defined subset of the population (sample frame) should be employed in most cases, however, random probabilistic sampling is not needed when everyone in the sampling frame will be included/ analysed.  For example, reporting on all the data from a good census is appropriate as a good census will identify everybody.  When using cluster sampling, such as a random sample of villages within a region, the methods need to be clearly stated as the precision of the final prevalence estimate incorporates the clustering effect. Convenience samples, such as a street survey or interviewing lots of people at a public gatherings are not considered to provide a representative sample of the base population.

© JBI, 2020. All rights reserved. JBI grants use of these tools for research purposes only. All other enquiries should be sent to jbisynthesis@adelaide.edu.au.

## 21. Was the sample size adequate?

The larger the sample, the narrower will be the confidence interval around the prevalence estimate, making the results more precise. An adequate sample size is important to ensure good precision of the final estimate. Ideally we are looking for evidence that the authors conducted a sample size calculation to determine an adequate sample size. This will estimate how many subjects are needed to produce a reliable estimate of the measure(s) of interest. For conditions with a low prevalence, a larger sample size is needed. Also consider sample sizes for subgroup (or characteristics) analyses, and whether these are appropriate. Sometimes, the study will be large enough (as in large national surveys) whereby a sample size calculation is not required. In these cases, sample size can be considered adequate.

When there is no sample size calculation and it is not a large national survey, the reviewers may consider conducting their own sample size analysis using the following formula: (Naing et al. 2006, Daniel 1999)

n= Z2P(1-P)

d2

Where:

n= sample size

Z = Z statistic for a level of confidence

P = Expected prevalence or proportion (in proportion of one; if 20%, P = 0.2)

d = precision (in proportion of one; if 5%, d=0.05)

**Ref:**

Naing L, Winn T, Rusli BN. Practical issues in calculating the sample size for prevalence studies Archives of Orofacial Sciences. 2006;1:9-14.

Daniel WW. Biostatistics: A Foundation for Analysis in the Health Sciences.

Edition. 7th ed. New York: John Wiley & Sons. 1999.

© JBI, 2020. All rights reserved. JBI grants use of these tools for research purposes only. All other enquiries should be sent to jbisynthesis@adelaide.edu.au.

**22. Were the study subjects and setting described in detail?**

Certain diseases or conditions vary in prevalence across different geographic regions and populations (e.g. Women vs. Men, sociodemographic variables between countries). The study sample should be described in sufficient detail so that other researchers can determine if it is comparable to the population of interest to them.

**23. Was data analysis conducted with sufficient coverage of the identified sample?**

Coverage bias can occur when not all subgroups of the identified sample respond at the same rate. For instance, you may have a very high response rate overall for your study, but the response rate for a certain subgroup (i.e. older adults) may be quite low.

**24. Were valid methods used for the identification of the condition?**

Here we are looking for measurement or classification bias. Many health problems are not easily diagnosed or defined and some measures may not be capable of including or excluding appropriate levels or stages of the health problem. If the outcomes were assessed based on existing definitions or diagnostic criteria, then the answer to this question is likely to be yes. If the outcomes were assessed using observer reported, or self-reported scales, the risk of over- or under-reporting is increased, and objectivity is compromised. Importantly, determine if the measurement tools used were validated instruments as this has a significant impact on outcome assessment validity.

**25. Was the condition measured in a standard, reliable way for all participants?**

Considerable judgment is required to determine the presence of some health outcomes. Having established the validity of the outcome measurement instrument (see item 6 of this scale), it is important to establish how the measurement was conducted. Were those involved in collecting data trained or educated in the use of the instrument/s? If there was more than one data collector, were they similar in terms of level of education, clinical or research experience, or level of responsibility in the piece of research being appraised? When there was more than one observer or collector, was there comparison of results from across the observers? Was the condition measured in the same way for all participants?

© JBI, 2020. All rights reserved. JBI grants use of these tools for research purposes only. All other enquiries should be sent to jbisynthesis@adelaide.edu.au.

**26. Was there appropriate statistical analysis?**

Importantly, the numerator and denominator should be clearly reported, and percentages should be given with confidence intervals. The methods section should be detailed enough for reviewers to identify the analytical technique used and how specific variables were measured. Additionally, it is also important to assess the appropriateness of the analytical strategy in terms of the assumptions associated with the approach as differing methods of analysis are based on differing assumptions about the data and how it will respond.

**27. Was the response rate adequate, and if not, was the low response rate managed appropriately?**

A large number of dropouts, refusals or “not founds” amongst selected subjects may diminish a study’s validity, as can a low response rates for survey studies. The authors should clearly discuss the response rate and any reasons for non-response and compare persons in the study to those not in the study, particularly with regards to their socio-demographic characteristics. If reasons for non-response appear to be unrelated to the outcome measured and the characteristics of non-responders are comparable to those who do respond in the study (addressed in question 5, coverage bias), the researchers may be able to justify a more modest response rate.

© JBI, 2020. All rights reserved. JBI grants use of these tools for research purposes only. All other enquiries should be sent to jbisynthesis@adelaide.edu.au.

# JBI CRITICAL APPRAISAL CHECKLIST FOR COHORT STUDIES

Reviewer_____Jamie Brannigan_______________________Date_____08/11/2021____________

Author_________Iizuka et al_________________________Year___2005___ Record Number__26/26___

| | Yes | No | Unclear | Not applicable |
|---|---|---|---|---|
| 13. Were the two groups similar and recruited from the same population? | ☐ | ☒ | ☐ | ☐ |
| 14. Were the exposures measured similarly to assign people to both exposed and unexposed groups? | ☒ | ☐ | ☐ | ☐ |
| 15. Was the exposure measured in a valid and reliable way? | ☒ | ☐ | ☐ | ☐ |
| 16. Were confounding factors identified? | ☐ | ☒ | ☐ | ☐ |
| 17. Were strategies to deal with confounding factors stated? | ☐ | ☐ | ☐ | ☒ |
| 18. Were the groups/participants free of the outcome at the start of the study (or at the moment of exposure)? | ☒ | ☐ | ☐ | ☐ |
| 19. Were the outcomes measured in a valid and reliable way? | ☒ | ☐ | ☐ | ☐ |
| 20. Was the follow up time reported and sufficient to be long enough for outcomes to occur? | ☒ | ☐ | ☐ | ☐ |
| 21. Was follow up complete, and if not, were the reasons to loss to follow up described and explored? | ☒ | ☐ | ☐ | ☐ |
| 22. Were strategies to address incomplete follow up utilized? | ☐ | ☐ | ☒ | ☐ |
| 23. Was appropriate statistical analysis used? | ☒ | ☐ | ☐ | ☐ |

Overall appraisal: Include ☒ Exclude ☐ Seek further info ☐

Comments (Including reason for exclusion)

Retrospective cohort study. The nature of the value representing spread in the descriptive statistics was not specified.

# EXPLANATION OF COHORT STUDIES CRITICAL APPRAISAL

How to Cite: *Moola S, Munn Z, Tufanaru C, Aromataris E, Sears K, Sfetcu R, Currie M, Qureshi R, Mattis P, Lisy K, Mu P-F. Chapter 7: Systematic reviews of etiology and risk . In: Aromataris E, Munn Z (Editors). JBI Manual for Evidence Synthesis.* JBI, 2020. Available from https://synthesismanual.jbi.global

## Cohort Studies Critical Appraisal Tool

Answers: Yes, No, Unclear or Not/Applicable

### 1. Were the two groups similar and recruited from the same population?

Check the paper carefully for descriptions of participants to determine if patients within and across groups have similar characteristics in relation to exposure (e.g. risk factor under investigation). The two groups selected for comparison should be as similar as possible in all characteristics except for their exposure status, relevant to the study in question. The authors should provide clear inclusion and exclusion criteria that they developed prior to recruitment of the study participants.

### 2. Were the exposures measured similarly to assign people to both exposed and unexposed groups?

A high quality study at the level of cohort design should mention or describe how the exposures were measured. The exposure measures should be clearly defined and described in detail. This will enable reviewers to assess whether or not the participants received the exposure of interest.

### 3. Was the exposure measured in a valid and reliable way?

The study should clearly describe the method of measurement of exposure. Assessing validity requires that a 'gold standard' is available to which the measure can be compared. The validity of exposure measurement usually relates to whether a current measure is appropriate or whether a measure of past exposure is needed.

Reliability refers to the processes included in an epidemiological study to check repeatability of measurements of the exposures. These usually include intra-observer reliability and inter-observer reliability.

### 4. Were confounding factors identified?

Confounding has occurred where the estimated intervention exposure effect is biased by the presence of some difference between the comparison groups (apart from the exposure investigated/of interest). Typical confounders include baseline characteristics, prognostic factors, or concomitant exposures (e.g. smoking). A confounder is a difference between the comparison groups and it influences the direction of the study results. A high quality study at the level of cohort design will identify the potential confounders and measure them (where possible). This is difficult for studies where behavioral, attitudinal or lifestyle factors may impact on the results.

© JBI, 2020. All rights reserved. JBI grants use of these tools for research purposes only. All other enquiries should be sent to jbisynthesis@adelaide.edu.au.

## 5. Were strategies to deal with confounding factors stated?

Strategies to deal with effects of confounding factors may be dealt within the study design or in data analysis. By matching or stratifying sampling of participants, effects of confounding factors can be adjusted for. When dealing with adjustment in data analysis, assess the statistics used in the study. Most will be some form of multivariate regression analysis to account for the confounding factors measured. Look out for a description of statistical methods as regression methods such as logistic regression are usually employed to deal with confounding factors/variables of interest.

## 6. Were the groups/participants free of the outcome at the start of the study (or at the moment of exposure)?

The participants should be free of the outcomes of interest at the start of the study. Refer to the 'methods' section in the paper for this information, which is usually found in descriptions of participant/sample recruitment, definitions of variables, and/or inclusion/exclusion criteria.

## 7. Were the outcomes measured in a valid and reliable way?

Read the methods section of the paper. If for e.g. lung cancer is assessed based on existing definitions or diagnostic criteria, then the answer to this question is likely to be yes. If lung cancer is assessed using observer reported, or self-reported scales, the risk of over- or under-reporting is increased, and objectivity is compromised. Importantly, determine if the measurement tools used were validated instruments as this has a significant impact on outcome assessment validity.

Having established the objectivity of the outcome measurement (e.g. lung cancer) instrument, it's important to establish how the measurement was conducted. Were those involved in collecting data trained or educated in the use of the instrument/s? (e.g. radiographers). If there was more than one data collector, were they similar in terms of level of education, clinical or research experience, or level of responsibility in the piece of research being appraised?

## 8. Was the follow up time reported and sufficient to be long enough for outcomes to occur?

The appropriate length of time for follow up will vary with the nature and characteristics of the population of interest and/or the intervention, disease or exposure. To estimate an appropriate duration of follow up, read across multiple papers and take note of the range for duration of follow up. The opinions of experts in clinical practice or clinical research may also assist in determining an appropriate duration of follow up. For example, a longer timeframe may be needed to examine the association between occupational exposure to asbestos and the risk of lung cancer. It is important, particularly in cohort studies that follow up is long enough to enable the outcomes. However, it should be remembered that the research question and outcomes being examined would probably dictate the follow up time.

© JBI, 2020. All rights reserved. JBI grants use of these tools for research purposes only. All other enquiries should be sent to jbisynthesis@adelaide.edu.au.

## 9. Was follow up complete, and if not, were the reasons to loss to follow up described and explored?

It is important in a cohort study that a greater percentage of people are followed up. As a general guideline, at least 80% of patients should be followed up. Generally a dropout rate of 5% or less is considered insignificant. A rate of 20% or greater is considered to significantly impact on the validity of the study. However, in observational studies conducted over a lengthy period of time a higher dropout rate is to be expected. A decision on whether to include or exclude a study because of a high dropout rate is a matter of judgement based on the reasons why people dropped out, and whether dropout rates were comparable in the exposed and unexposed groups.

Reporting of efforts to follow up participants that dropped out may be regarded as an indicator of a well conducted study. Look for clear and justifiable description of why people were left out, excluded, dropped out etc. If there is no clear description or a statement in this regards, this will be a 'No'.

## 10. Were strategies to address incomplete follow up utilized?

Some people may withdraw due to change in employment or some may die; however, it is important that their outcomes are assessed. Selection bias may occur as a result of incomplete follow up. Therefore, participants with unequal follow up periods must be taken into account in the analysis, which should be adjusted to allow for differences in length of follow up periods. This is usually done by calculating rates which use person-years at risk, i.e. considering time in the denominator.

## 11. Was appropriate statistical analysis used?

As with any consideration of statistical analysis, consideration should be given to whether there was a more appropriate alternate statistical method that could have been used. The methods section of cohort studies should be detailed enough for reviewers to identify which analytical techniques were used (in particular, regression or stratification) and how specific confounders were measured.

For studies utilizing regression analysis, it is useful to identify if the study identified which variables were included and how they related to the outcome. If stratification was the analytical approach used, were the strata of analysis defined by the specified variables? Additionally, it is also important to assess the appropriateness of the analytical strategy in terms of the assumptions associated with the approach as differing methods of analysis are based on differing assumptions about the data and how it will respond.

© JBI, 2020. All rights reserved. JBI grants use of these tools for research purposes only. All other enquiries should be sent to jbisynthesis@adelaide.edu.au.

# JBI CRITICAL APPRAISAL CHECKLIST FOR COHORT STUDIES

Reviewer_____Jamie Brannigan_______________________Date_____03/05/2021____________

Author_________Kalb et al_________________________Year___2012___ Record Number__12/26___

| | Yes | No | Unclear | Not applicable |
|---|---|---|---|---|
| 24. Were the two groups similar and recruited from the same population? | ☒ | ☐ | ☐ | ☐ |
| 25. Were the exposures measured similarly to assign people<br>26. to both exposed and unexposed groups? | ☒ | ☐ | ☐ | ☐ |
| 27. Was the exposure measured in a valid and reliable way? | ☒ | ☐ | ☐ | ☐ |
| 28. Were confounding factors identified? | ☒ | ☐ | ☐ | ☐ |
| 29. Were strategies to deal with confounding factors stated? | ☐ | ☒ | ☐ | ☐ |
| 30. Were the groups/participants free of the outcome at the start of the study (or at the moment of exposure)? | ☒ | ☐ | ☐ | ☐ |
| 31. Were the outcomes measured in a valid and reliable way? | ☐ | ☐ | ☒ | ☐ |
| 32. Was the follow up time reported and sufficient to be long enough for outcomes to occur? | ☐ | ☐ | ☐ | ☒ |
| 33. Was follow up complete, and if not, were the reasons to loss to follow up described and explored? | ☐ | ☐ | ☐ | ☒ |
| 34. Were strategies to address incomplete follow up utilized? | ☐ | ☐ | ☐ | ☒ |
| 35. Was appropriate statistical analysis used? | ☒ | ☐ | ☐ | ☐ |

Overall appraisal: Include ☒ Exclude ☐ Seek further info ☐

Comments (Including reason for exclusion)

Retrospective nature means that follow up is non-applicable. Outcome measure validity is unclear as there is no universally accepted method for determining dysphagia. Various confounding factors were stated, however strategies (e.g. multivariate regression or subgrouping) were not mentioned.

© JBI, 2020. All rights reserved. JBI grants use of these tools for research purposes only. All other enquiries should be sent to jbisynthesis@adelaide.edu.au.

# EXPLANATION OF COHORT STUDIES CRITICAL APPRAISAL

How to Cite: *Moola S, Munn Z, Tufanaru C, Aromataris E, Sears K, Sfetcu R, Currie M, Qureshi R, Mattis P, Lisy K, Mu P-F. Chapter 7: Systematic reviews of etiology and risk . In: Aromataris E, Munn Z (Editors). JBI Manual for Evidence Synthesis.* JBI, 2020. Available from https://synthesismanual.jbi.global

## Cohort Studies Critical Appraisal Tool

Answers: Yes, No, Unclear or Not/Applicable

### 1. Were the two groups similar and recruited from the same population?

Check the paper carefully for descriptions of participants to determine if patients within and across groups have similar characteristics in relation to exposure (e.g. risk factor under investigation). The two groups selected for comparison should be as similar as possible in all characteristics except for their exposure status, relevant to the study in question. The authors should provide clear inclusion and exclusion criteria that they developed prior to recruitment of the study participants.

### 2. Were the exposures measured similarly to assign people to both exposed and unexposed groups?

A high quality study at the level of cohort design should mention or describe how the exposures were measured. The exposure measures should be clearly defined and described in detail. This will enable reviewers to assess whether or not the participants received the exposure of interest.

### 3. Was the exposure measured in a valid and reliable way?

The study should clearly describe the method of measurement of exposure. Assessing validity requires that a 'gold standard' is available to which the measure can be compared. The validity of exposure measurement usually relates to whether a current measure is appropriate or whether a measure of past exposure is needed.

Reliability refers to the processes included in an epidemiological study to check repeatability of measurements of the exposures. These usually include intra-observer reliability and inter-observer reliability.

### 4. Were confounding factors identified?

Confounding has occurred where the estimated intervention exposure effect is biased by the presence of some difference between the comparison groups (apart from the exposure investigated/of interest). Typical confounders include baseline characteristics, prognostic factors, or concomitant exposures (e.g. smoking). A confounder is a difference between the comparison groups and it influences the direction of the study results. A high quality study at the level of cohort design will identify the potential confounders and measure them (where possible). This is difficult for studies where behavioral, attitudinal or lifestyle factors may impact on the results.

© JBI, 2020. All rights reserved. JBI grants use of these tools for research purposes only. All other enquiries should be sent to jbisynthesis@adelaide.edu.au.

5. **Were strategies to deal with confounding factors stated?**

Strategies to deal with effects of confounding factors may be dealt within the study design or in data analysis. By matching or stratifying sampling of participants, effects of confounding factors can be adjusted for. When dealing with adjustment in data analysis, assess the statistics used in the study. Most will be some form of multivariate regression analysis to account for the confounding factors measured. Look out for a description of statistical methods as regression methods such as logistic regression are usually employed to deal with confounding factors/variables of interest.

6. **Were the groups/participants free of the outcome at the start of the study (or at the moment of exposure)?**

The participants should be free of the outcomes of interest at the start of the study. Refer to the 'methods' section in the paper for this information, which is usually found in descriptions of participant/sample recruitment, definitions of variables, and/or inclusion/exclusion criteria.

7. **Were the outcomes measured in a valid and reliable way?**

Read the methods section of the paper. If for e.g. lung cancer is assessed based on existing definitions or diagnostic criteria, then the answer to this question is likely to be yes. If lung cancer is assessed using observer reported, or self-reported scales, the risk of over- or under-reporting is increased, and objectivity is compromised. Importantly, determine if the measurement tools used were validated instruments as this has a significant impact on outcome assessment validity.

Having established the objectivity of the outcome measurement (e.g. lung cancer) instrument, it's important to establish how the measurement was conducted. Were those involved in collecting data trained or educated in the use of the instrument/s? (e.g. radiographers). If there was more than one data collector, were they similar in terms of level of education, clinical or research experience, or level of responsibility in the piece of research being appraised?

8. **Was the follow up time reported and sufficient to be long enough for outcomes to occur?**

The appropriate length of time for follow up will vary with the nature and characteristics of the population of interest and/or the intervention, disease or exposure. To estimate an appropriate duration of follow up, read across multiple papers and take note of the range for duration of follow up. The opinions of experts in clinical practice or clinical research may also assist in determining an appropriate duration of follow up. For example, a longer timeframe may be needed to examine the association between occupational exposure to asbestos and the risk of lung cancer. It is important, particularly in cohort studies that follow up is long enough to enable the outcomes. However, it should be remembered that the research question and outcomes being examined would probably dictate the follow up time.

© JBI, 2020. All rights reserved. JBI grants use of these tools for research purposes only. All other enquiries should be sent to jbisynthesis@adelaide.edu.au.

### 9. Was follow up complete, and if not, were the reasons to loss to follow up described and explored?

It is important in a cohort study that a greater percentage of people are followed up. As a general guideline, at least 80% of patients should be followed up. Generally a dropout rate of 5% or less is considered insignificant. A rate of 20% or greater is considered to significantly impact on the validity of the study. However, in observational studies conducted over a lengthy period of time a higher dropout rate is to be expected. A decision on whether to include or exclude a study because of a high dropout rate is a matter of judgement based on the reasons why people dropped out, and whether dropout rates were comparable in the exposed and unexposed groups.

Reporting of efforts to follow up participants that dropped out may be regarded as an indicator of a well conducted study. Look for clear and justifiable description of why people were left out, excluded, dropped out etc. If there is no clear description or a statement in this regards, this will be a 'No'.

### 10. Were strategies to address incomplete follow up utilized?

Some people may withdraw due to change in employment or some may die; however, it is important that their outcomes are assessed. Selection bias may occur as a result of incomplete follow up. Therefore, participants with unequal follow up periods must be taken into account in the analysis, which should be adjusted to allow for differences in length of follow up periods. This is usually done by calculating rates which use person-years at risk, i.e. considering time in the denominator.

### 11. Was appropriate statistical analysis used?

As with any consideration of statistical analysis, consideration should be given to whether there was a more appropriate alternate statistical method that could have been used. The methods section of cohort studies should be detailed enough for reviewers to identify which analytical techniques were used (in particular, regression or stratification) and how specific confounders were measured.

For studies utilizing regression analysis, it is useful to identify if the study identified which variables were included and how they related to the outcome. If stratification was the analytical approach used, were the strata of analysis defined by the specified variables? Additionally, it is also important to assess the appropriateness of the analytical strategy in terms of the assumptions associated with the approach as differing methods of analysis are based on differing assumptions about the data and how it will respond.

© JBI, 2020. All rights reserved. JBI grants use of these tools for research purposes only. All other enquiries should be sent to jbisynthesis@adelaide.edu.au.

# JBI CRITICAL APPRAISAL CHECKLIST FOR COHORT STUDIES

Reviewer_____Jamie Brannigan______________________Date_____03/05/2021___________

Author________Karlberg et al________________________Year___1991___ Record Number__18/26___

| | Yes | No | Unclear | Not applicable |
|---|---|---|---|---|
| 36. Were the two groups similar and recruited from the same population? | ☐ | ☐ | ☐ | ☒ |
| 37. Were the exposures measured similarly to assign people to both exposed and unexposed groups? | ☐ | ☐ | ☐ | ☒ |
| 38. Was the exposure measured in a valid and reliable way? | ☒ | ☐ | ☐ | ☐ |
| 39. Were confounding factors identified? | ☒ | ☐ | ☐ | ☐ |
| 40. Were strategies to deal with confounding factors stated? | ☒ | ☐ | ☐ | ☐ |
| 41. Were the groups/participants free of the outcome at the start of the study (or at the moment of exposure)? | ☒ | ☐ | ☐ | ☐ |
| 42. Were the outcomes measured in a valid and reliable way? | ☒ | ☐ | ☐ | ☐ |
| 43. Was the follow up time reported and sufficient to be long enough for outcomes to occur? | ☒ | ☐ | ☐ | ☐ |
| 44. Was follow up complete, and if not, were the reasons to loss to follow up described and explored? | ☒ | ☐ | ☐ | ☐ |
| 45. Were strategies to address incomplete follow up utilized? | ☐ | ☐ | ☐ | ☒ |
| 46. Was appropriate statistical analysis used? | ☒ | ☐ | ☐ | ☐ |

Overall appraisal: Include ☒ Exclude ☐ Seek further info ☐

Comments (Including reason for exclusion)

Prospective cohort study with no control group. Control study was performed due to the risk of a confounder in the form of adaptation to repeated postural perturbation.

# EXPLANATION OF COHORT STUDIES CRITICAL APPRAISAL

How to Cite: *Moola S, Munn Z, Tufanaru C, Aromataris E, Sears K, Sfetcu R, Currie M, Qureshi R, Mattis P, Lisy K, Mu P-F. Chapter 7: Systematic reviews of etiology and risk . In: Aromataris E, Munn Z (Editors). JBI Manual for Evidence Synthesis.* JBI, 2020. Available from https://synthesismanual.jbi.global

## Cohort Studies Critical Appraisal Tool

Answers: Yes, No, Unclear or Not/Applicable

### 1. Were the two groups similar and recruited from the same population?

Check the paper carefully for descriptions of participants to determine if patients within and across groups have similar characteristics in relation to exposure (e.g. risk factor under investigation). The two groups selected for comparison should be as similar as possible in all characteristics except for their exposure status, relevant to the study in question. The authors should provide clear inclusion and exclusion criteria that they developed prior to recruitment of the study participants.

### 2. Were the exposures measured similarly to assign people to both exposed and unexposed groups?

A high quality study at the level of cohort design should mention or describe how the exposures were measured. The exposure measures should be clearly defined and described in detail. This will enable reviewers to assess whether or not the participants received the exposure of interest.

### 3. Was the exposure measured in a valid and reliable way?

The study should clearly describe the method of measurement of exposure. Assessing validity requires that a 'gold standard' is available to which the measure can be compared. The validity of exposure measurement usually relates to whether a current measure is appropriate or whether a measure of past exposure is needed.

Reliability refers to the processes included in an epidemiological study to check repeatability of measurements of the exposures. These usually include intra-observer reliability and inter-observer reliability.

### 4. Were confounding factors identified?

Confounding has occurred where the estimated intervention exposure effect is biased by the presence of some difference between the comparison groups (apart from the exposure investigated/of interest). Typical confounders include baseline characteristics, prognostic factors, or concomitant exposures (e.g. smoking). A confounder is a difference between the comparison groups and it influences the direction of the study results. A high quality study at the level of cohort design will identify the potential confounders and measure them (where possible). This is difficult for studies where behavioral, attitudinal or lifestyle factors may impact on the results.

© JBI, 2020. All rights reserved. JBI grants use of these tools for research purposes only. All other enquiries should be sent to jbisynthesis@adelaide.edu.au.

**5. Were strategies to deal with confounding factors stated?**

Strategies to deal with effects of confounding factors may be dealt within the study design or in data analysis. By matching or stratifying sampling of participants, effects of confounding factors can be adjusted for. When dealing with adjustment in data analysis, assess the statistics used in the study. Most will be some form of multivariate regression analysis to account for the confounding factors measured. Look out for a description of statistical methods as regression methods such as logistic regression are usually employed to deal with confounding factors/variables of interest.

**6. Were the groups/participants free of the outcome at the start of the study (or at the moment of exposure)?**

The participants should be free of the outcomes of interest at the start of the study. Refer to the 'methods' section in the paper for this information, which is usually found in descriptions of participant/sample recruitment, definitions of variables, and/or inclusion/exclusion criteria.

**7. Were the outcomes measured in a valid and reliable way?**

Read the methods section of the paper. If for e.g. lung cancer is assessed based on existing definitions or diagnostic criteria, then the answer to this question is likely to be yes. If lung cancer is assessed using observer reported, or self-reported scales, the risk of over- or under-reporting is increased, and objectivity is compromised. Importantly, determine if the measurement tools used were validated instruments as this has a significant impact on outcome assessment validity.

Having established the objectivity of the outcome measurement (e.g. lung cancer) instrument, it's important to establish how the measurement was conducted. Were those involved in collecting data trained or educated in the use of the instrument/s? (e.g. radiographers). If there was more than one data collector, were they similar in terms of level of education, clinical or research experience, or level of responsibility in the piece of research being appraised?

**8. Was the follow up time reported and sufficient to be long enough for outcomes to occur?**

The appropriate length of time for follow up will vary with the nature and characteristics of the population of interest and/or the intervention, disease or exposure. To estimate an appropriate duration of follow up, read across multiple papers and take note of the range for duration of follow up.  The opinions of experts in clinical practice or clinical research may also assist in determining an appropriate duration of follow up. For example, a longer timeframe may be needed to examine the association between occupational exposure to asbestos and the risk of lung cancer. It is important, particularly in cohort studies that follow up is long enough to enable the outcomes.  However, it should be remembered that the research question and outcomes being examined would probably dictate the follow up time.

© JBI, 2020. All rights reserved. JBI grants use of these tools for research purposes only. All other enquiries should be sent to jbisynthesis@adelaide.edu.au.

## 9. Was follow up complete, and if not, were the reasons to loss to follow up described and explored?

It is important in a cohort study that a greater percentage of people are followed up. As a general guideline, at least 80% of patients should be followed up. Generally a dropout rate of 5% or less is considered insignificant. A rate of 20% or greater is considered to significantly impact on the validity of the study. However, in observational studies conducted over a lengthy period of time a higher dropout rate is to be expected. A decision on whether to include or exclude a study because of a high dropout rate is a matter of judgement based on the reasons why people dropped out, and whether dropout rates were comparable in the exposed and unexposed groups.

Reporting of efforts to follow up participants that dropped out may be regarded as an indicator of a well conducted study. Look for clear and justifiable description of why people were left out, excluded, dropped out etc. If there is no clear description or a statement in this regards, this will be a 'No'.

## 10. Were strategies to address incomplete follow up utilized?

Some people may withdraw due to change in employment or some may die; however, it is important that their outcomes are assessed. Selection bias may occur as a result of incomplete follow up. Therefore, participants with unequal follow up periods must be taken into account in the analysis, which should be adjusted to allow for differences in length of follow up periods. This is usually done by calculating rates which use person-years at risk, i.e. considering time in the denominator.

## 11. Was appropriate statistical analysis used?

As with any consideration of statistical analysis, consideration should be given to whether there was a more appropriate alternate statistical method that could have been used. The methods section of cohort studies should be detailed enough for reviewers to identify which analytical techniques were used (in particular, regression or stratification) and how specific confounders were measured.

For studies utilizing regression analysis, it is useful to identify if the study identified which variables were included and how they related to the outcome. If stratification was the analytical approach used, were the strata of analysis defined by the specified variables? Additionally, it is also important to assess the appropriateness of the analytical strategy in terms of the assumptions associated with the approach as differing methods of analysis are based on differing assumptions about the data and how it will respond.

© JBI, 2020. All rights reserved. JBI grants use of these tools for research purposes only. All other enquiries should be sent to jbisynthesis@adelaide.edu.au.

# JBI CRITICAL APPRAISAL CHECKLIST FOR SYSTEMATIC REVIEWS AND RESEARCH SYNTHESES

Reviewer _________Jamie Brannigan________________Date________03/05/2021_______________

Author_______Lacey et al______________________Year___2019___ Record Number_22/26____

| | Yes | No | Unclear | Not applicable |
|---|---|---|---|---|
| 58. Is the review question clearly and explicitly stated? | ☒ | ☐ | ☐ | ☐ |
| 59. Were the inclusion criteria appropriate for the review question? | ☒ | ☐ | ☐ | ☐ |
| 60. Was the search strategy appropriate? | ☒ | ☐ | ☐ | ☐ |
| 61. Were the sources and resources used to search for studies adequate? | ☒ | ☐ | ☐ | ☐ |
| 62. Were the criteria for appraising studies appropriate? | ☐ | ☐ | ☒ | ☐ |
| 63. Was critical appraisal conducted by two or more reviewers independently? | ☐ | ☐ | ☒ | ☐ |
| 64. Were there methods to minimize errors in data extraction? | ☒ | ☐ | ☐ | ☐ |
| 65. Were the methods used to combine studies appropriate? | ☐ | ☐ | ☐ | ☒ |
| 66. Was the likelihood of publication bias assessed? | ☐ | ☐ | ☒ | ☐ |
| 67. Were recommendations for policy and/or practice supported by the reported data? | ☒ | ☐ | ☐ | ☐ |
| 68. Were the specific directives for new research appropriate? | ☒ | ☐ | ☐ | ☐ |

Overall appraisal: Include ☒ Exclude ☐ Seek further info ☐

Comments (Including reason for exclusion)

This review was a scoping review, not a true systematic review. Hence quality assessment was not thoroughly performed and there was no quantitative meta-analysis. This is still appropriate for inclusion in the systematic review.

# JBI CRITICAL APPRAISAL CHECKLIST FOR SYSTEMATIC REVIEWS AND RESEARCH SYNTHESIS

How to cite: *Aromataris E, Fernandez R, Godfrey C, Holly C, Kahlil H, Tungpunkom P. Summarizing systematic reviews: methodological development, conduct and reporting of an Umbrella review approach. Int J Evid Based Healthc. 2015;13(3):132-40.*

When conducting an umbrella review using the JBI method, the critical appraisal instrument for Systematic Reviews should be used.

The primary and secondary reviewer should discuss each item in the appraisal instrument for each study included in their review. In particular, discussions should focus on what is considered acceptable to the aims of the review in terms of the specific study characteristics. When appraising systematic reviews this discussion may include issues such as what represents an adequate search strategy or appropriate methods of synthesis. The reviewers should be clear on what constitutes acceptable levels of information to allocate a positive appraisal compared with a negative, or response of "unclear". This discussion should ideally take place before the reviewers independently conduct the appraisal.

Within umbrella reviews, quantitative or qualitative systematic reviews may be incorporated, as well as meta-analyses of existing research. There are 11 questions to guide the appraisal of systematic reviews or meta-analyses. Each question should be answered as "yes", "no", or "unclear". Not applicable "NA" is also provided as an option and may be appropriate in rare instances.

### 12. Is the review question clearly and explicitly stated?

The review question is an essential step in the systematic review process. A well-articulated question defines the scope of the review and aids in the development of the search strategy to locate the relevant evidence. An explicitly stated question, formulated around its PICO (Population, Intervention, Comparator, Outcome) elements aids both the review team in the conduct of the review and the reader in determining if the review has achieved its objectives. Ideally the review question should be articulated in a published protocol; however this will not always be the case with many reviews that are located.

### 13. Were the inclusion criteria appropriate for the review question?

The inclusion criteria should be identifiable from, and match the review question. The necessary elements of the PICO should be explicit and clearly defined. The inclusion criteria should be detailed and the included reviews should clearly be eligible when matched against the stated inclusion criteria. Appraisers of meta-analyses will find that inclusion criteria may include criteria around the ability to conduct statistical analyses which would not be the norm for a systematic review. The types of included studies should be relevant to the review question, for example, an umbrella review aiming to summarize a range of effective non-pharmacological interventions for aggressive behaviors amongst elderly patients with dementia will limit itself to including systematic reviews and meta-analyses that synthesize quantitative studies assessing the various interventions; qualitative or economic reviews would not be included.

### 14. Was the search strategy appropriate?

A systematic review should provide evidence of the search strategy that has been used to locate the evidence. This may be found in the methods section of the review report in some cases, or as an appendix that may be provided as supplementary information to the review publication. A systematic review should present a clear search strategy that addresses each of the identifiable PICO components of the review question. Some reviews may also provide a description of the approach to searching and how the terms that were ultimately used were derived, though due to limits on word counts in journals this may be more the norm in online only publications. There

© JBI, 2020. All rights reserved. JBI grants use of these tools for research purposes only. All other enquiries should be sent to jbisynthesis@adelaide.edu.au.

should be evidence of logical and relevant keywords and terms and also evidence that Subject

Headings and Indexing terms have been used in the conduct of the search. Limits on the search should also be considered and their potential impact; for example, if a date limit was used, was this appropriate and/or justified? If only English language studies were included, will such a language bias have an impact on the review? The response to these considerations will depend, in part, on the review question.

### 15. Were the sources and resources used to search for studies adequate?

A systematic review should attempt to identify "all" the available evidence and as such there should be evidence of a comprehensive search strategy. Multiple electronic databases should be searched including major bibliographic citation databases such as MEDLINE and CINAHL. Ideally, other databases that are relevant to the review question should also be searched, for example, a systematic review with a question about a physical therapy intervention should also look to search the PEDro database, whilst a review focusing on an educational intervention should also search the ERIC. Reviews of effectiveness should aim to search trial registries. A comprehensive search is the ideal way to minimize publication bias, as a result, a well conducted systematic review should also attempt to search for grey literature, or "unpublished" studies; this may involve searching websites relevant to the review question, or thesis repositories.

### 16. Were the criteria for appraising studies appropriate?

The systematic review should present a clear statement that critical appraisal was conducted and provide the details of the items that were used to assess the included studies. This may be presented in the methods of the review, as an appendix of supplementary information, or as a reference to a source that can be located. The tools or instruments used should be appropriate for the review question asked and the type of research conducted. For example, a systematic review of effectiveness should present a tool or instrument that addresses aspects of validity for experimental studies and randomized controlled trials such as randomization and blinding – if the review includes observational research to answer the same question a different tool would be more appropriate. Similarly, a review assessing diagnostic test accuracy may refer to the recognized QUADAS[1] tool.

### 17. Was critical appraisal conducted by two or more reviewers independently?

Critical appraisal or some similar assessment of the quality of the literature included in a systematic review is essential. A key characteristic to minimize bias or systematic error in the conduct of a systematic review is to have the critical appraisal of the included studies completed independently and in duplicate by members of the review team. The systematic review should present a clear statement that critical appraisal was conducted by at least two reviewers working independently from each other and conferring where necessary to reach decision regarding study quality and eligibility on the basis of quality.

### 18. Were there methods to minimize errors in data extraction?

Efforts made by review authors during data extraction can also minimize bias or systematic errors in the conduct of a systematic review. Strategies to minimize bias may include conducting all data extraction in duplicate and independently, using specific tools or instruments to guide data extraction and some evidence of piloting or training around their use.

### 19. Were the methods used to combine studies appropriate?

A synthesis of the evidence is a key feature of a systematic review. The synthesis that is presented should be appropriate for the review question and the stated type of systematic review and evidence it refers to. If a meta-analysis has been conducted this needs to be reviewed carefully.

© JBI, 2020. All rights reserved. JBI grants use of these tools for research purposes only. All other enquiries should be sent to jbisynthesis@adelaide.edu.au.

Was it appropriate to combine the studies? Have the reviewers assessed heterogeneity statistically and provided some explanation for heterogeneity that may be present? Often, where heterogeneous studies are included in the systematic review, narrative synthesis will be an appropriate method for presenting the results of multiple studies. If a qualitative review, are the methods that have been used to synthesize findings congruent with the stated methodology of the review? Is there adequate descriptive and explanatory information to support the final synthesized findings that have been constructed from the findings sourced from the original research?

### 20. Was the likelihood of publication bias assessed?

As mentioned, a comprehensive search strategy is the best means by which a review author may alleviate the impact of publication bias on the results of the review. Reviews may also present statistical tests such as Egger's test or funnel plots to also assess the potential presence of publication bias and its potential impact on the results of the review. This question will not be applicable to systematic reviews of qualitative evidence.

### 21. Were recommendations for policy and/or practice supported by the reported data?

Whilst the first nine (9) questions specifically look to identify potential bias in the conduct of a systematic review, the final questions are more indictors of review quality rather than validity. Ideally a review should present recommendations for policy and practice. Where these recommendations are made there should be a clear link to the results of the review. Is there evidence that the strength of the findings and the quality of the research been considered in the formulation of review recommendations?

### 22. Were the specific directives for new research appropriate?

The systematic review process is recognized for its ability to identify where gaps in the research, or knowledge base, around a particular topic exist. Most systematic review authors will provide some indication, often in the discussion section of the report, of where future research direction should lie. Where evidence is scarce or sample sizes that support overall estimates of effect are small and effect estimates are imprecise, repeating similar research to those identified by the review may be necessary and appropriate. In other instances, the case for new research questions to investigate the topic may be warranted.

## REFERENCES

2. Whiting P, Rutjes AWS, Reitsma JB, Bossuyt PMM, Kleijnen J. The development of QUADAS: a tool for the quality assessment of studies of diagnostic accuracy included in systematic reviews. BMC Medical Research Methodology. 2003;3:25 doi:10.1186/1471-2288-3-25.

© JBI, 2020. All rights reserved. JBI grants use of these tools for research purposes only. All other enquiries should be sent to jbisynthesis@adelaide.edu.au.

# JBI CRITICAL APPRAISAL CHECKLIST FOR RANDOMIZED CONTROLLED TRIALS

Reviewer _____Jamie Brannigan___________________________ Date______03/05/2021_____________

Author__________Landny___et___al_________________________________Year__2020___ Record Number__17/26___

| | Yes | No | Unclear | NA |
|---|---|---|---|---|
| 69. Was true randomization used for assignment of participants to treatment groups? | ☒ | ☐ | ☐ | ☐ |
| 70. Was allocation to treatment groups concealed? | ☐ | ☐ | ☐ | ☒ |
| 71. Were treatment groups similar at the baseline? | ☒ | ☐ | ☐ | ☐ |
| 72. Were participants blind to treatment assignment? | ☐ | ☒ | ☐ | ☐ |
| 73. Were those delivering treatment blind to treatment assignment? | ☐ | ☒ | ☐ | ☐ |
| 74. Were outcomes assessors blind to treatment assignment? | ☒ | ☐ | ☐ | ☐ |
| 75. Were treatment groups treated identically other than the intervention of interest? | ☒ | ☐ | ☐ | ☐ |
| 76. Was follow up complete and if not, were differences between groups in terms of their follow up adequately described and analyzed? | ☒ | ☐ | ☐ | ☐ |
| 77. Were participants analyzed in the groups to which they were randomized? | ☒ | ☐ | ☐ | ☐ |
| 78. Were outcomes measured in the same way for treatment groups? | ☒ | ☐ | ☐ | ☐ |
| 79. Were outcomes measured in a reliable way? | ☒ | ☐ | ☐ | ☐ |
| 80. Was appropriate statistical analysis used? | ☒ | ☐ | ☐ | ☐ |
| 81. Was the trial design appropriate, and any deviations from the standard RCT design (individual randomization, parallel groups) accounted for in the conduct and analysis of the trial? | ☒ | ☐ | ☐ | ☐ |

Overall appraisal: Include ☒ Exclude ☐ Seek further info ☐

Comments (Including reason for exclusion)

Blinding to assessment is practically difficult when using branded medical devices. Allocation to treatment group concealment was irrelevant as each subject received a collar and there was subsequent crossover.

# EXPLANATION FOR THE CRITICAL APPRAISAL TOOL FOR RCTS WITH INDIVIDUAL PARTICIPANTS IN PARALLEL GROUPS

How to cite: *Tufanaru C, Munn Z, Aromataris E, Campbell J, Hopp L. Chapter 3: Systematic reviews of effectiveness. In: Aromataris E, Munn Z (Editors). JBI Manual for Evidence Synthesis.* JBI, 2020. Available from https://synthesismanual.jbi.global

Answers: Yes, No, Unclear or Not/Applicable

**Critical Appraisal Tool for RCTs (individual participants in parallel groups)**

**1. Was true randomization used for assignment of participants to treatment groups?**

The differences between participants included in compared groups constitutes a threat to the internal validity of a study exploring causal relationships. If participants are not allocated to treatment and control groups by random assignment there is a risk that the allocation is influenced by the known characteristics of the participants and these differences between the groups may distort the comparability of the groups. A true random assignment of participants to the groups means that a procedure is used that allocates the participants to groups purely based on chance, not influenced by the known characteristics of the participants. Check the details about the randomization procedure used for allocation of the participants to study groups. Was a true chance (random) procedure used? For example, was a list of random numbers used? Was a computer-generated list of random numbers used?

**2. Was allocation to groups concealed?**

If those allocating participants to the compared groups are aware of which group is next in the allocation process, that is, treatment or control, there is a risk that they may deliberately and purposefully intervene in the allocation of patients by preferentially allocating patients to the treatment group or to the control group and therefore this may distort the implementation of allocation process indicated by the randomization and therefore the results of the study may be distorted. Concealment of allocation (allocation concealment) refers to procedures that prevent those allocating patients from knowing before allocation which treatment or control is next in the allocation process. Check the details about the procedure used for allocation concealment. Was an appropriate allocation concealment procedure used? For example, was central randomization used? Were sequentially numbered, opaque and sealed envelopes used? Were coded drug packs used?

**3. Were treatment groups similar at the baseline?**

The differences between participants included in compared groups constitute a threat to the internal validity of a study exploring causal relationships. If there are differences between participants included in compared groups there is a risk of selection bias. If there are differences between participants included in the compared groups maybe the 'effect' cannot

© JBI, 2020. All rights reserved. JBI grants use of these tools for research purposes only. All other enquiries should be sent to jbisynthesis@adelaide.edu.au.

be attributed to the potential 'cause' (the examined intervention or treatment), as maybe it is plausible that the 'effect' may be explained by the differences between participants, that is, by selection bias. Check the characteristics reported for participants. Are the participants from the compared groups similar with regards to the characteristics that may explain the effect even in the absence of the 'cause', for example, age, severity of the disease, stage of the disease, co-existing conditions and so on? Check the proportions of participants with specific relevant characteristics in the compared groups. Check the means of relevant measurements in the compared groups (pain scores; anxiety scores; etc.). *[Note: Do NOT only consider the P-value for the statistical testing of the differences between groups with regards to the baseline characteristics.]*

4. **Were participants blind to treatment assignment?**

   If participants are aware of their allocation to the treatment group or to the control group there is the risk that they may behave differently and respond or react differently to the intervention of interest or to the control intervention respectively compared to the situations when they are not aware of treatment allocation and therefore the results of the study may be distorted. Blinding of participants is used in order to minimize this risk. Blinding of the participants refers to procedures that prevent participants from knowing which group they are allocated. If blinding of participants is used, participants are not aware if they are in the group receiving the treatment of interest or if they are in any other group receiving the control interventions. Check the details reported in the article about the blinding of participants with regards to treatment assignment. Was an appropriate blinding procedure used? For example, were identical capsules or syringes used? Were identical devices used? Be aware of different terms used, blinding is sometimes also called masking.

5. **Were those delivering treatment blind to treatment assignment?**

   If those delivering treatment are aware of participants' allocation to the treatment group or to the control group there is the risk that they may behave differently with the participants from the treatment group and the participants from the control group, or that they may treat them differently, compared to the situations when they are not aware of treatment allocation and this may influence the implementation of the compared treatments and the results of the study may be distorted. Blinding of those delivering treatment is used in order to minimize this risk. Blinding of those delivering treatment refers to procedures that prevent those delivering treatment from knowing which group they are treating, that is those delivering treatment are not aware if they are treating the group receiving the treatment of interest or if they are treating any other group receiving the control interventions. Check the details reported in the article about the blinding of those delivering treatment with regards to treatment assignment. Is there any information in the article about those delivering the treatment? Were those delivering the treatment unaware of the assignments of participants to the compared groups?

© JBI, 2020. All rights reserved. JBI grants use of these tools for research purposes only. All other enquiries should be sent to jbisynthesis@adelaide.edu.au.

6. **Were outcomes assessors blind to treatment assignment?**

If those assessing the outcomes are aware of participants' allocation to the treatment group or to the control group there is the risk that they may behave differently with the participants from the treatment group and the participants from the control group compared to the situations when they are not aware of treatment allocation and therefore there is the risk that the measurement of the outcomes may be distorted and the results of the study may be distorted. Blinding of outcomes assessors is used in order to minimize this risk. Check the details reported in the article about the blinding of outcomes assessors with regards to treatment assignment. Is there any information in the article about outcomes assessors? Were those assessing the treatment's effects on outcomes unaware of the assignments of participants to the compared groups?

7. **Were treatment groups treated identically other than the intervention of interest?**

In order to attribute the 'effect' to the 'cause' (the treatment or intervention of interest), assuming that there is no selection bias, there should be no other difference between the groups in terms of treatment or care received, other than the manipulated 'cause' (the treatment or intervention controlled by the researchers). If there are other exposures or treatments occurring at the same time with the 'cause' (the treatment or intervention of interest), other than the 'cause', then potentially the 'effect' cannot be attributed to the examined 'cause' (the investigated treatment), as it is plausible that the 'effect' may be explained by other exposures or treatments occurring at the same time with the 'cause' (the treatment of interest). Check the reported exposures or interventions received by the compared groups. Are there other exposures or treatments occurring at the same time with the 'cause'? Is it plausible that the 'effect' may be explained by other exposures or treatments occurring at the same time with the 'cause'? Is it clear that there is no other difference between the groups in terms of treatment or care received, other than the treatment or intervention of interest?

8. **Was follow up complete and if not, were differences between groups in terms of their follow up adequately described and analyzed?**

For this question, follow up refers to the time period from the moment of random allocation (random assignment or randomization) to compared groups to the end time of the trial. This critical appraisal question asks if there is complete knowledge (measurements, observations etc.) for the entire duration of the trial as previously defined (that is, from the moment of random allocation to the end time of the trial), for all randomly allocated participants. If there is incomplete follow up, that is incomplete knowledge about all randomly allocated participants, this is known in the methodological literature as the post-assignment attrition. As RCTs are not perfect, there is almost always post-assignment attrition, and the focus of this question is on the appropriate exploration of post-assignment attrition (description of loss to follow up, description of the reasons for loss to follow up, the estimation of the impact of loss

© JBI, 2020. All rights reserved. JBI grants use of these tools for research purposes only. All other enquiries should be sent to jbisynthesis@adelaide.edu.au.

to follow up on the effects etc.). If there are differences with regards to the loss to follow up between the compared groups in an RCT, these differences represent a threat to the internal validity of a randomized experimental study exploring causal effects, as these differences may provide a plausible alternative explanation for the observed 'effect' even in the absence of the 'cause' (the treatment or intervention of interest). When appraising an RCT, check if there were differences with regards to the loss to follow up between the compared groups. If follow up was incomplete (that is, there is incomplete information on all participants), examine the reported details about the strategies used in order to address incomplete follow up, such as descriptions of loss to follow up (absolute numbers; proportions; reasons for loss to follow up) and impact analyses (the analyses of the impact of loss to follow up on results). Was there a description of the incomplete follow up (number of participants and the specific reasons for loss to follow up)? It is important to note that with regards to loss to follow up, it is not enough to know the number of participants and the proportions of participants with incomplete data; the reasons for loss to follow up are essential in the analysis of risk of bias; even if the numbers and proportions of participants with incomplete data are similar or identical in compared groups, if the patterns of reasons for loss to follow up are different (for example, side effects caused by the intervention of interest, lost contact etc.), these may impose a risk of bias if not appropriately explored and considered in the analysis. If there are differences between groups with regards to the loss to follow up (numbers/proportions and reasons), was there an analysis of patterns of loss to follow up? If there are differences between the groups with regards to the loss to follow up, was there an analysis of the impact of the loss to follow up on the results? [Note: Question 8 is NOT about intention-to-treat (ITT) analysis; question 9 is about ITT analysis.]

**9. Were participants analyzed in the groups to which they were randomized?**

This question is about the intention-to-treat (ITT) analysis. There are different statistical analysis strategies available for the analysis of data from randomized controlled trials, such as intention-to-treat analysis (known also as intent to treat; abbreviated, ITT), per-protocol analysis, and as-treated analysis. In the ITT analysis the participants are analyzed in the groups to which they were randomized, regardless of whether they actually participated or not in those groups for the entire duration of the trial, received the experimental intervention or control intervention as planned or whether they were compliant or not with the planned experimental intervention or control intervention. The ITT analysis compares the outcomes for participants from the initial groups created by the initial random allocation of participants to those groups. Check if ITT was reported; check the details of the ITT. Were participants analyzed in the groups to which they were initially randomized, regardless of whether they actually participated in those groups, and regardless of whether they actually received the planned interventions? *[Note: The ITT analysis is a type of statistical analysis recommended in the Consolidated Standards of Reporting Trials (CONSORT) statement on best practices in trials reporting, and it is considered a marker of good methodological quality of the analysis of results of a randomized trial. The ITT is estimating the effect of offering the intervention, that*

© JBI, 2020. All rights reserved. JBI grants use of these tools for research purposes only. All other enquiries should be sent to jbisynthesis@adelaide.edu.au.

*is, the effect of instructing the participants to use or take the intervention; the ITT it is not estimating the effect of actually receiving the intervention of interest.]*

**10. Were outcomes measured in the same way for treatment groups?**

If the outcome (the 'effect') is not measured in the same way in the compared groups there is a threat to the internal validity of a study exploring a causal relationship as the differences in outcome measurements may be confused with an effect of the treatment (the 'cause'). Check if the outcomes were measured in the same way. Same instrument or scale used? Same measurement timing? Same measurement procedures and instructions?

**11. Were outcomes measured in a reliable way?**

Unreliability of outcome measurements is one threat that weakens the validity of inferences about the statistical relationship between the 'cause' and the 'effect' estimated in a study exploring causal effects. Unreliability of outcome measurements is one of the different plausible explanations for errors of statistical inference with regards to the existence and the magnitude of the effect determined by the treatment ('cause'). Check the details about the reliability of measurement such as the number of raters, training of raters, the intra-rater reliability, and the inter-raters reliability within the study (not as reported in external sources). This question is about the reliability of the measurement performed in the study, it is not about the validity of the measurement instruments/scales used in the study. *[Note: Two other important threats that weaken the validity of inferences about the statistical relationship between the 'cause' and the 'effect' are low statistical power and the violation of the assumptions of statistical tests. These other two threats are explored within Question 12).]*

**12. Was appropriate statistical analysis used?**

Inappropriate statistical analysis may cause errors of statistical inference with regards to the existence and the magnitude of the effect determined by the treatment ('cause'). Low statistical power and the violation of the assumptions of statistical tests are two important threats that weaken the validity of inferences about the statistical relationship between the 'cause' and the 'effect'. Check the following aspects: if the assumptions of statistical tests were respected; if appropriate statistical power analysis was performed; if appropriate effect sizes were used; if appropriate statistical procedures or methods were used given the number and type of dependent and independent variables, the number of study groups, the nature of the relationship between the groups (independent or dependent groups), and the objectives of statistical analysis (association between variables; prediction; survival analysis etc.).

**13. Was the trial design appropriate for the topic, and any deviations from the standard RCT design accounted for in the conduct and analysis?**

Certain RCT designs, such as the crossover RCT, should only be conducted when appropriate. Alternative designs may also present additional risks of bias if not accounted for in the design and analysis.

© JBI, 2020. All rights reserved. JBI grants use of these tools for research purposes only. All other enquiries should be sent to jbisynthesis@adelaide.edu.au.

Crossover trials should only be conducted in people with a chronic, stable condition, where the intervention produces a short term effect (i.e. relief in symptoms). Crossover trials should ensure there is an appropriate period of washout between treatments.

Cluster RCTs randomize groups of individuals, forming ‘clusters.’ When we are assessing outcomes on an individual level in cluster trials, there are unit-of-analysis issues, as individuals within a cluster are correlated. This should be taken into account by the study authors when conducting analysis, and ideally authors will report the intra-cluster correlation coefficient.

Stepped-wedge RCTs may be appropriate when it is expected the intervention will do more good than harm, or due to logistical, practical or financial considerations in the roll out of a new treatment/intervention. Data analysis in these trials should be conducted appropriately, taking into account the effects of time.

© JBI, 2020. All rights reserved. JBI grants use of these tools for research purposes only. All other enquiries should be sent to jbisynthesis@adelaide.edu.au.

# JBI CRITICAL APPRAISAL CHECKLIST FOR STUDIES REPORTING PREVALENCE DATA

Reviewer _____________Jamie Brannigan_____________Date______03/05/2021________________

Author___________Lewis et al_________________Year__2011___ Record Number__8/26____

| | Yes | No | Unclear | Not applicable |
|---|---|---|---|---|
| 82. Was the sample frame appropriate to address the target population? | ☒ | ☐ | ☐ | ☐ |
| 83. Were study participants sampled in an appropriate way? | ☒ | ☐ | ☐ | ☐ |
| 84. Was the sample size adequate? | ☐ | ☐ | ☒ | ☐ |
| 85. Were the study subjects and the setting described in detail? | ☒ | ☐ | ☐ | ☐ |
| 86. Was the data analysis conducted with sufficient coverage of the identified sample? | ☒ | ☐ | ☐ | ☐ |
| 87. Were valid methods used for the identification of the condition? | ☐ | ☐ | ☒ | ☐ |
| 88. Was the condition measured in a standard, reliable way for all participants? | ☒ | ☐ | ☐ | ☐ |
| 89. Was there appropriate statistical analysis? | ☒ | ☐ | ☐ | ☐ |
| 90. Was the response rate adequate, and if not, was the low response rate managed appropriately? | ☐ | ☐ | ☐ | ☒ |

Overall appraisal: Include ☒ Exclude ☐ Seek further info ☐

Comments (Including reason for exclusion)

Pressure ulcer data is suitable for quantitative meta-analysis. Validity of identification unclear due to retrospective assessment of care records and the exclusive findings of submandibular abrasion.

# JBI CRITICAL APPRAISAL CHECKLIST FOR STUDIES REPORTING PREVALENCE DATA

*How to cite: Munn Z, Moola S, Lisy K, Riitano D, Tufanaru C. Methodological guidance for systematic reviews of observational epidemiological studies reporting prevalence and incidence data. Int J Evid Based Healthc. 2015;13(3):147–153.*

Answers: Yes, No, Unclear or Not/Applicable

**28. Was the sample frame appropriate to address the target population?**

This question relies upon knowledge of the broader characteristics of the population of interest and the geographical area. If the study is of women with breast cancer, knowledge of at least the characteristics, demographics and medical history is needed. The term "target population" should not be taken to infer every individual from everywhere or with similar disease or exposure characteristics. Instead, give consideration to specific population characteristics in the study, including age range, gender, morbidities, medications, and other potentially influential factors. For example, a sample frame may not be appropriate to address the target population if a certain group has been used (such as those working for one organisation, or one profession) and the results then inferred to the target population (i.e. working adults). A sample frame may be appropriate when it includes almost all the members of the target population (i.e. a census, or a complete list of participants or complete registry data).

**29. Were study participants recruited in an appropriate way?**

Studies may report random sampling from a population, and the methods section should report how sampling was performed. Random probabilistic sampling from a defined subset of the population (sample frame) should be employed in most cases, however, random probabilistic sampling is not needed when everyone in the sampling frame will be included/ analysed. For example, reporting on all the data from a good census is appropriate as a good census will identify everybody. When using cluster sampling, such as a random sample of villages within a region, the methods need to be clearly stated as the precision of the final prevalence estimate incorporates the clustering effect. Convenience samples, such as a street survey or interviewing lots of people at a public gatherings are not considered to provide a representative sample of the base population.

© JBI, 2020. All rights reserved. JBI grants use of these tools for research purposes only. All other enquiries should be sent to jbisynthesis@adelaide.edu.au.

**30. Was the sample size adequate?**

The larger the sample, the narrower will be the confidence interval around the prevalence estimate, making the results more precise. An adequate sample size is important to ensure good precision of the final estimate. Ideally we are looking for evidence that the authors conducted a sample size calculation to determine an adequate sample size. This will estimate how many subjects are needed to produce a reliable estimate of the measure(s) of interest. For conditions with a low prevalence, a larger sample size is needed. Also consider sample sizes for subgroup (or characteristics) analyses, and whether these are appropriate. Sometimes, the study will be large enough (as in large national surveys) whereby a sample size calculation is not required. In these cases, sample size can be considered adequate.

When there is no sample size calculation and it is not a large national survey, the reviewers may consider conducting their own sample size analysis using the following formula: (Naing et al. 2006, Daniel 1999)

n= Z2P(1-P)

d2

Where:

n= sample size

Z = Z statistic for a level of confidence

P = Expected prevalence or proportion (in proportion of one; if 20%, P = 0.2)

d = precision (in proportion of one; if 5%, d=0.05)

**Ref:**

Naing L, Winn T, Rusli BN. Practical issues in calculating the sample size for prevalence studies Archives of Orofacial Sciences. 2006;1:9-14.

Daniel WW. Biostatistics: A Foundation for Analysis in the Health Sciences.

Edition. 7th ed. New York: John Wiley & Sons. 1999.

© JBI, 2020. All rights reserved. JBI grants use of these tools for research purposes only. All other enquiries should be sent to jbisynthesis@adelaide.edu.au.

31. **Were the study subjects and setting described in detail?**

Certain diseases or conditions vary in prevalence across different geographic regions and populations (e.g. Women vs. Men, sociodemographic variables between countries). The study sample should be described in sufficient detail so that other researchers can determine if it is comparable to the population of interest to them.

32. **Was data analysis conducted with sufficient coverage of the identified sample?**

Coverage bias can occur when not all subgroups of the identified sample respond at the same rate. For instance, you may have a very high response rate overall for your study, but the response rate for a certain subgroup (i.e. older adults) may be quite low.

33. **Were valid methods used for the identification of the condition?**

Here we are looking for measurement or classification bias. Many health problems are not easily diagnosed or defined and some measures may not be capable of including or excluding appropriate levels or stages of the health problem. If the outcomes were assessed based on existing definitions or diagnostic criteria, then the answer to this question is likely to be yes. If the outcomes were assessed using observer reported, or self-reported scales, the risk of over- or under-reporting is increased, and objectivity is compromised. Importantly, determine if the measurement tools used were validated instruments as this has a significant impact on outcome assessment validity.

34. **Was the condition measured in a standard, reliable way for all participants?**

Considerable judgment is required to determine the presence of some health outcomes. Having established the validity of the outcome measurement instrument (see item 6 of this scale), it is important to establish how the measurement was conducted. Were those involved in collecting data trained or educated in the use of the instrument/s? If there was more than one data collector, were they similar in terms of level of education, clinical or research experience, or level of responsibility in the piece of research being appraised? When there was more than one observer or collector, was there comparison of results from across the observers? Was the condition measured in the same way for all participants?

© JBI, 2020. All rights reserved. JBI grants use of these tools for research purposes only. All other enquiries should be sent to jbisynthesis@adelaide.edu.au.

35. **Was there appropriate statistical analysis?**

Importantly, the numerator and denominator should be clearly reported, and percentages should be given with confidence intervals. The methods section should be detailed enough for reviewers to identify the analytical technique used and how specific variables were measured. Additionally, it is also important to assess the appropriateness of the analytical strategy in terms of the assumptions associated with the approach as differing methods of analysis are based on differing assumptions about the data and how it will respond.

36. **Was the response rate adequate, and if not, was the low response rate managed appropriately?**

A large number of dropouts, refusals or “not founds” amongst selected subjects may diminish a study’s validity, as can a low response rates for survey studies. The authors should clearly discuss the response rate and any reasons for non-response and compare persons in the study to those not in the study, particularly with regards to their socio-demographic characteristics. If reasons for non-response appear to be unrelated to the outcome measured and the characteristics of non-responders are comparable to those who do respond in the study (addressed in question 5, coverage bias), the researchers may be able to justify a more modest response rate.

© JBI, 2020. All rights reserved. JBI grants use of these

tools for research purposes only. All other enquiries should be sent to jbisynthesis@adelaide.edu.au.

# JBI Critical Appraisal Checklist for Case Series

Reviewer _____Jamie Brannigan_____________________Date_____03/05/2021__________________

Author________Liew and Hill______________________________Year___1994__ Record Number__15/26___

| | Yes | No | Unclear | Not applicable |
|---|---|---|---|---|
| • Were there clear criteria for inclusion in the case series? | ☐ | ☒ | ☐ | ☐ |
| • Was the condition measured in a standard, reliable way for all participants included in the case series? | ☒ | ☐ | ☐ | ☐ |
| • Were valid methods used for identification of the condition for all participants included in the case series? | ☐ | ☒ | ☐ | ☐ |
| • Did the case series have consecutive inclusion of participants? | ☐ | ☒ | ☐ | ☐ |
| • Did the case series have complete inclusion of participants? | ☐ | ☒ | ☐ | ☐ |
| • Was there clear reporting of the demographics of the participants in the study? | ☐ | ☐ | ☒ | ☐ |
| • Was there clear reporting of clinical information of the participants? | ☒ | ☐ | ☐ | ☐ |
| • Were the outcomes or follow up results of cases clearly reported? | ☒ | ☐ | ☐ | ☐ |
| • Was there clear reporting of the presenting site(s)/clinic(s) demographic information? | ☒ | ☐ | ☐ | ☐ |
| • Was statistical analysis appropriate? | ☐ | ☐ | ☐ | ☒ |

Overall appraisal: Include ☒ Exclude ☐ Seek further info ☐

Comments (Including reason for exclusion)

The series is not appropriate for any inclusion in a quantitative analysis, but the induvial case reports give more thorough natural histories of pressure ulcer development that may be useful in the narrative component.

# INTRODUCTION TO THE CASE SERIES CRITICAL APPRAISAL TOOL

*How to cite:* Munn Z, Barker T, Moola S, Tufanaru C, Stern C, McArthur A, Stephenson M, Aromataris E. Methodological quality of case series studies, JBI Evidence Synthesis, doi: 10.11124/JBISRIR-D-19-00099

The definition of a case series varies across the medical literature, which has resulted in inconsistent use of this term (Appendix 1).[1-3] The gamut of case studies is wide, with some studies claiming to be a case series realistically being nothing more than a collection of case reports, with others more akin to cohort studies or even quasi-experimental before and after studies. This has created difficulty in assigning 'case series' a position in the hierarchy of evidence and identifying and appropriate critical appraisal tool.[1, 2]

Dekkers et al. define a case series as a study in which 'only patients with the outcome are sampled (either those who have an exposure or those who are selected without regard to exposure), which does not permit calculation of an absolute risk.'[1p.39] The outcome could be a disease or a disease related outcome. This is contrasted to cohort studies where sampling is based on exposure (or characteristic), and case- control studies where there is a comparison group without the disease.

The completeness of a case series contributes to its reliability.[1] Studies that indicate a consecutive and complete inclusion are more reliable than those that do not. For example, a case series that states 'we included all patients (24) with osteosarcoma who presented to our clinic between March 2005 and June 2006' is more reliable than a study that simply states 'we report a case series of 24 people with osteosarcoma.'

For the purposes of this checklist, we agree with the principles outlined in the Dekker et al. paper, and define case series as studies where only patients with a certain disease or disease-related outcome are sampled. Some of the items below relate to risk of bias, whilst others relate to ensuring adequate reporting and statistical analysis. A response of 'no' to any of the questions below negatively impacts the quality of a case series.

© JBI, 2020. All rights reserved. JBI grants use of these tools for research purposes only. All other enquiries should be sent to jbisynthesis@adelaide.edu.au.

# TOOL GUIDANCE

Answers: Yes, No, Unclear or Not/Applicable

1. **Were there clear criteria for inclusion in the case series?**

   The authors should provide clear inclusion (and exclusion criteria where appropriate) for the study participants. The inclusion/exclusion criteria should be specified (e.g., risk, stage of disease progression) with sufficient detail and all the necessary information critical to the study.

2. **Was the condition measured in a standard, reliable way for all participants included in the case series?**

   The study should clearly describe the method of measurement of the condition. This should be done in a standard (i.e. same way for all patients) and reliable (i.e. repeatable and reproducible results) way.

3. **Were valid methods used for identification of the condition for all participants included in the case series?**

   Many health problems are not easily diagnosed or defined and some measures may not be capable of including or excluding appropriate levels or stages of the health problem. If the outcomes were assessed based on existing definitions or diagnostic criteria, then the answer to this question is likely to be yes. If the outcomes were assessed using observer reported, or self-reported scales, the risk of over- or under-reporting is increased, and objectivity is compromised. Importantly, determine if the measurement tools used were validated instruments as this has a significant impact on outcome assessment validity.

4. **Did the case series have consecutive inclusion of participants?**

   Studies that indicate a consecutive inclusion are more reliable than those that do not. For example, a case series that states 'we included all patients (24) with osteosarcoma who presented to our clinic between March 2005 and June 2006' is more reliable than a study that simply states 'we report a case series of 24 people with osteosarcoma.'

5. **Did the case series have complete inclusion of participants?**

   The completeness of a case series contributes to its reliability (1). Studies that indicate a complete inclusion are more reliable than those that do not. A stated above, a case series that states 'we included all patients (24) with osteosarcoma who presented to our clinic between March 2005 and June 2006' is more reliable than a study that simply states 'we report a case series of 24 people with osteosarcoma.'

6. **Was there clear reporting of the demographics of the participants in the study?**

   The case series should clearly describe relevant participant's demographics such as the following information where relevant: participant's age, sex, education, geographic region, ethnicity, time period, education.

© JBI, 2020. All rights reserved. JBI grants use of these tools for research purposes only. All other enquiries should be sent to jbisynthesis@adelaide.edu.au.

## 7. Was there clear reporting of clinical information of the participants?

There should be clear reporting of clinical information of the participants such as the following information where relevant: disease status, comorbidities, stage of disease, previous interventions/treatment, results of diagnostic tests, etc.

## 8. Were the outcomes or follow-up results of cases clearly reported?

The results of any intervention or treatment should be clearly reported in the case series. A good case study should clearly describe the clinical condition post-intervention in terms of the presence or lack of symptoms. The outcomes of management/treatment when presented as images or figures can help in conveying the information to the reader/clinician. It is important that adverse events are clearly documented and described, particularly a new or unique condition is being treated or when a new drug or treatment is used. In addition, unanticipated events, if any that may yield new or useful information should be identified and clearly described.

## 9. Was there clear reporting of the presenting site(s)/clinic(s) demographic information?

Certain diseases or conditions vary in prevalence across different geographic regions and populations (e.g. women vs. men, sociodemographic variables between countries). The study sample should be described in sufficient detail so that other researchers can determine if it is comparable to the population of interest to them.

## 10. Was statistical analysis appropriate?

As with any consideration of statistical analysis, consideration should be given to whether there was a more appropriate alternate statistical method that could have been used. The methods section of studies should be detailed enough for reviewers to identify which analytical techniques were used and whether these were suitable.

© JBI, 2020. All rights reserved. JBI grants use of these tools for research purposes only. All other enquiries should be sent to jbisynthesis@adelaide.edu.au.

# REFERENCES

1. Dekkers OM, Egger M, Altman DG, Vandenbroucke JP. Distinguishing case series from cohort studies. Annals of Internal Medicine. 2012;156(1 Part 1):37-40.

2. Esene IN, Ngu J, El Zoghby M, Solaroglu I, Sikod AM, Kotb A et al. Case series and descriptive cohort studies in neurosurgery: the confusion and solution. Child's Nervous System. 2014;30(8):1321-32.

3. Abu-Zidan FM, Abbas AK, Hefny AF. Clinical “case series”: a concept analysis. African Health Sciences. 2012;12(4):557-62.

4 Straus SE, Richardson WS, Glasziou P, Haynes RB. Evidence-based medicine: How to practice and teach EBM. 3rd Edition ed: Elsevier 2005.

## Appendix 1: Case series definitions:

‘A report on a series of patients with an outcome of interest. No control group is involved.’(4) (p 279)

‘A case series is a descriptive study involving a group of patients who all have the same disease or condition: the aim is to describe common and differing characteristics of a particular group of individuals’ (Oxford Handbook of medical statistics)

‘A group or series of case reports involving patients who were given similar treatment. Reports of case series usually contain detailed information about the individual patients. This includes demographic information (for example, age, gender, ethnic origin) and information on diagnosis, treatment, response to treatment, and follow-up after treatment.’ Law K, Howick J. OCEBM Table of Evidence Glossary. 2013 [cited 2014 10th January]; Available from: http://www.cebm.net/index.aspx?o=1116

‘A **case series** (also known as a clinical **series**) is a type of medical research study that tracks subjects with a known exposure, such as patients who have received a similar treatment, or examines their medical records for exposure and outcome.’ Wikipedia

‘A study which makes observations on a series of individuals, usually all receiving the same intervention, with no control group. Comments: At this stage it is unclear whether case series should be included in Cochrane systematic reviews, but we have left them in the list so that working groups can consider whether there are circumstances in which it would be appropriate to include them, and to assess risk of bias. A particular reason for including case series might be where they provide evidence relating to adverse effects of an intervention. Potential examples of risk of bias might be that if a case series does not [attempt to] recruit consecutive participants, this might introduce a risk of selection bias, while some case series could be at risk of detection bias, if the circumstances in which adverse effects are reported (or elicited) are not standardised.’ http://bmg.cochrane.org/research-projectscochrane-risk-bias-tool

© JBI, 2020. All rights reserved. JBI grants use of these tools for research purposes only. All other enquiries should be sent to jbisynthesis@adelaide.edu.au.

# JBI CRITICAL APPRAISAL CHECKLIST FOR STUDIES REPORTING PREVALENCE DATA

Reviewer ______________Jamie Brannigan_______________Date______03/05/2021_________________

Author__________Molano et al________________Year__2004___ Record Number__4/26____

| | Yes | No | Unclear | Not applicable |
|---|---|---|---|---|
| Was the sample frame appropriate to address the target population? 91. | ☒ | ☐ | ☐ | ☐ |
| 92. Were study participants sampled in an appropriate way? | ☒ | ☐ | ☐ | ☐ |
| 93. Was the sample size adequate? | ☐ | ☐ | ☒ | ☐ |
| 94. Were the study subjects and the setting described in detail? | ☒ | ☐ | ☐ | ☐ |
| 95. Was the data analysis conducted with sufficient coverage of the identified sample? | ☒ | ☐ | ☐ | ☐ |
| 96. Were valid methods used for the identification of the condition? | ☒ | ☐ | ☐ | ☐ |
| Was the condition measured in a standard, reliable way for all participants? 97. | ☒ | ☐ | ☐ | ☐ |
| 98. Was there appropriate statistical analysis? | ☒ | ☐ | ☐ | ☐ |
| 99. Was the response rate adequate, and if not, was the low response rate managed appropriately? | ☐ | ☐ | ☐ | ☒ |

Overall appraisal: Include ☒ Exclude ☐ Seek further info ☐

Comments (Including reason for exclusion)

Suitable for quantitative meta-analysis.

# JBI CRITICAL APPRAISAL CHECKLIST FOR STUDIES REPORTING PREVALENCE DATA

*How to cite: Munn Z, Moola S, Lisy K, Riitano D, Tufanaru C. Methodological guidance for systematic reviews of observational epidemiological studies reporting prevalence and incidence data. Int J Evid Based Healthc. 2015;13(3):147–153.*

Answers: Yes, No, Unclear or Not/Applicable

**37. Was the sample frame appropriate to address the target population?**

This question relies upon knowledge of the broader characteristics of the population of interest and the geographical area. If the study is of women with breast cancer, knowledge of at least the characteristics, demographics and medical history is needed. The term “target population” should not be taken to infer every individual from everywhere or with similar disease or exposure characteristics. Instead, give consideration to specific population characteristics in the study, including age range, gender, morbidities, medications, and other potentially influential factors. For example, a sample frame may not be appropriate to address the target population if a certain group has been used (such as those working for one organisation, or one profession) and the results then inferred to the target population (i.e. working adults). A sample frame may be appropriate when it includes almost all the members of the target population (i.e. a census, or a complete list of participants or complete registry data).

**38. Were study participants recruited in an appropriate way?**

Studies may report random sampling from a population, and the methods section should report how sampling was performed. Random probabilistic sampling from a defined subset of the population (sample frame) should be employed in most cases, however, random probabilistic sampling is not needed when everyone in the sampling frame will be included/ analysed. For example, reporting on all the data from a good census is appropriate as a good census will identify everybody. When using cluster sampling, such as a random sample of villages within a region, the methods need to be clearly stated as the precision of the final prevalence estimate incorporates the clustering effect. Convenience samples, such as a street survey or interviewing lots of people at a public gatherings are not considered to provide a representative sample of the base population.

© JBI, 2020. All rights reserved. JBI grants use of these tools for research purposes only. All other enquiries should be sent to jbisynthesis@adelaide.edu.au.

**39. Was the sample size adequate?**

The larger the sample, the narrower will be the confidence interval around the prevalence estimate, making the results more precise. An adequate sample size is important to ensure good precision of the final estimate. Ideally we are looking for evidence that the authors conducted a sample size calculation to determine an adequate sample size. This will estimate how many subjects are needed to produce a reliable estimate of the measure(s) of interest. For conditions with a low prevalence, a larger sample size is needed. Also consider sample sizes for subgroup (or characteristics) analyses, and whether these are appropriate. Sometimes, the study will be large enough (as in large national surveys) whereby a sample size calculation is not required. In these cases, sample size can be considered adequate.

When there is no sample size calculation and it is not a large national survey, the reviewers may consider conducting their own sample size analysis using the following formula: (Naing et al. 2006, Daniel 1999)

n= Z2P(1-P)

d2

Where:

n= sample size

Z = Z statistic for a level of confidence

P = Expected prevalence or proportion (in proportion of one; if 20%, P = 0.2)

d = precision (in proportion of one; if 5%, d=0.05)

**Ref:**

Naing L, Winn T, Rusli BN. Practical issues in calculating the sample size for prevalence studies Archives of Orofacial Sciences. 2006;1:9-14.

Daniel WW. Biostatistics: A Foundation for Analysis in the Health Sciences.

Edition. 7th ed. New York: John Wiley & Sons. 1999.

© JBI, 2020. All rights reserved. JBI grants use of these tools for research purposes only. All other enquiries should be sent to jbisynthesis@adelaide.edu.au.

**40. Were the study subjects and setting described in detail?**

Certain diseases or conditions vary in prevalence across different geographic regions and populations (e.g. Women vs. Men, sociodemographic variables between countries). The study sample should be described in sufficient detail so that other researchers can determine if it is comparable to the population of interest to them.

**41. Was data analysis conducted with sufficient coverage of the identified sample?**

Coverage bias can occur when not all subgroups of the identified sample respond at the same rate. For instance, you may have a very high response rate overall for your study, but the response rate for a certain subgroup (i.e. older adults) may be quite low.

**42. Were valid methods used for the identification of the condition?**

Here we are looking for measurement or classification bias. Many health problems are not easily diagnosed or defined and some measures may not be capable of including or excluding appropriate levels or stages of the health problem. If the outcomes were assessed based on existing definitions or diagnostic criteria, then the answer to this question is likely to be yes. If the outcomes were assessed using observer reported, or self-reported scales, the risk of over- or under-reporting is increased, and objectivity is compromised. Importantly, determine if the measurement tools used were validated instruments as this has a significant impact on outcome assessment validity.

**43. Was the condition measured in a standard, reliable way for all participants?**

Considerable judgment is required to determine the presence of some health outcomes. Having established the validity of the outcome measurement instrument (see item 6 of this scale), it is important to establish how the measurement was conducted. Were those involved in collecting data trained or educated in the use of the instrument/s? If there was more than one data collector, were they similar in terms of level of education, clinical or research experience, or level of responsibility in the piece of research being appraised? When there was more than one observer or collector, was there comparison of results from across the observers? Was the condition measured in the same way for all participants?

© JBI, 2020. All rights reserved. JBI grants use of these tools for research purposes only. All other enquiries should be sent to jbisynthesis@adelaide.edu.au.

**44. Was there appropriate statistical analysis?**

Importantly, the numerator and denominator should be clearly reported, and percentages should be given with confidence intervals. The methods section should be detailed enough for reviewers to identify the analytical technique used and how specific variables were measured. Additionally, it is also important to assess the appropriateness of the analytical strategy in terms of the assumptions associated with the approach as differing methods of analysis are based on differing assumptions about the data and how it will respond.

**45. Was the response rate adequate, and if not, was the low response rate managed appropriately?**

A large number of dropouts, refusals or "not founds" amongst selected subjects may diminish a study's validity, as can a low response rates for survey studies. The authors should clearly discuss the response rate and any reasons for non-response and compare persons in the study to those not in the study, particularly with regards to their socio-demographic characteristics. If reasons for non-response appear to be unrelated to the outcome measured and the characteristics of non-responders are comparable to those who do respond in the study (addressed in question 5, coverage bias), the researchers may be able to justify a more modest response rate.

© JBI, 2020. All rights reserved. JBI grants use of these tools for research purposes only. All other enquiries should be sent to jbisynthesis@adelaide.edu.au.

# JBI CRITICAL APPRAISAL CHECKLIST FOR STUDIES REPORTING PREVALENCE DATA

Reviewer _____________Jamie Brannigan_______________Date______03/05/2021_________________

Author__________Molinari et al_________________Year__2012___ Record Number__2/26____

| | Yes | No | Unclear | Not applicable |
|---|---|---|---|---|
| 100. Was the sample frame appropriate to address the target population? | ☒ | ☐ | ☐ | ☐ |
| 101. Were study participants sampled in an appropriate way? | ☒ | ☐ | ☐ | ☐ |
| 102. Was the sample size adequate? | ☐ | ☐ | ☒ | ☐ |
| 103. Were the study subjects and the setting described in detail? | ☒ | ☐ | ☐ | ☐ |
| 104. Was the data analysis conducted with sufficient coverage of the identified sample? | ☒ | ☐ | ☐ | ☐ |
| 105. Were valid methods used for the identification of the condition? | ☐ | ☐ | ☒ | ☐ |
| 106. Was the condition measured in a standard, reliable way for all participants? | ☒ | ☐ | ☐ | ☐ |
| 107. Was there appropriate statistical analysis? | ☒ | ☐ | ☐ | ☐ |
| 108. Was the response rate adequate, and if not, was the low response rate managed appropriately? | ☐ | ☐ | ☐ | ☒ |

Overall appraisal: Include ☒ Exclude ☐ Seek further info ☐

Comments (Including reason for exclusion)

Suitable for quantitative meta-analysis. Unclear validity of complication identification as unsure which complications were screened in the chart review.

# JBI CRITICAL APPRAISAL CHECKLIST FOR STUDIES REPORTING PREVALENCE DATA

*How to cite: Munn Z, Moola S, Lisy K, Riitano D, Tufanaru C. Methodological guidance for systematic reviews of observational epidemiological studies reporting prevalence and incidence data. Int J Evid Based Healthc. 2015;13(3):147–153.*

Answers: Yes, No, Unclear or Not/Applicable

**46. Was the sample frame appropriate to address the target population?**

This question relies upon knowledge of the broader characteristics of the population of interest and the geographical area. If the study is of women with breast cancer, knowledge of at least the characteristics, demographics and medical history is needed. The term “target population” should not be taken to infer every individual from everywhere or with similar disease or exposure characteristics. Instead, give consideration to specific population characteristics in the study, including age range, gender, morbidities, medications, and other potentially influential factors. For example, a sample frame may not be appropriate to address the target population if a certain group has been used (such as those working for one organisation, or one profession) and the results then inferred to the target population (i.e. working adults). A sample frame may be appropriate when it includes almost all the members of the target population (i.e. a census, or a complete list of participants or complete registry data).

**47. Were study participants recruited in an appropriate way?**

Studies may report random sampling from a population, and the methods section should report how sampling was performed. Random probabilistic sampling from a defined subset of the population (sample frame) should be employed in most cases, however, random probabilistic sampling is not needed when everyone in the sampling frame will be included/ analysed. For example, reporting on all the data from a good census is appropriate as a good census will identify everybody. When using cluster sampling, such as a random sample of villages within a region, the methods need to be clearly stated as the precision of the final prevalence estimate incorporates the clustering effect. Convenience samples, such as a street survey or interviewing lots of people at a public gatherings are not considered to provide a representative sample of the base population.

© JBI, 2020. All rights reserved. JBI grants use of these tools for research purposes only. All other enquiries should be sent to jbisynthesis@adelaide.edu.au.

## 48. Was the sample size adequate?

The larger the sample, the narrower will be the confidence interval around the prevalence estimate, making the results more precise. An adequate sample size is important to ensure good precision of the final estimate. Ideally we are looking for evidence that the authors conducted a sample size calculation to determine an adequate sample size. This will estimate how many subjects are needed to produce a reliable estimate of the measure(s) of interest. For conditions with a low prevalence, a larger sample size is needed. Also consider sample sizes for subgroup (or characteristics) analyses, and whether these are appropriate. Sometimes, the study will be large enough (as in large national surveys) whereby a sample size calculation is not required. In these cases, sample size can be considered adequate.

When there is no sample size calculation and it is not a large national survey, the reviewers may consider conducting their own sample size analysis using the following formula: (Naing et al. 2006, Daniel 1999)

n= Z2P(1-P)

d2

Where:

n= sample size

Z = Z statistic for a level of confidence

P = Expected prevalence or proportion (in proportion of one; if 20%, P = 0.2)

d = precision (in proportion of one; if 5%, d=0.05)

**Ref:**

Naing L, Winn T, Rusli BN. Practical issues in calculating the sample size for prevalence studies Archives of Orofacial Sciences. 2006;1:9-14.

Daniel WW. Biostatistics: A Foundation for Analysis in the Health Sciences.

Edition. 7th ed. New York: John Wiley & Sons. 1999.

© JBI, 2020. All rights reserved. JBI grants use of these tools for research purposes only. All other enquiries should be sent to jbisynthesis@adelaide.edu.au.

**49. Were the study subjects and setting described in detail?**

Certain diseases or conditions vary in prevalence across different geographic regions and populations (e.g. Women vs. Men, sociodemographic variables between countries). The study sample should be described in sufficient detail so that other researchers can determine if it is comparable to the population of interest to them.

**50. Was data analysis conducted with sufficient coverage of the identified sample?**

Coverage bias can occur when not all subgroups of the identified sample respond at the same rate. For instance, you may have a very high response rate overall for your study, but the response rate for a certain subgroup (i.e. older adults) may be quite low.

**51. Were valid methods used for the identification of the condition?**

Here we are looking for measurement or classification bias. Many health problems are not easily diagnosed or defined and some measures may not be capable of including or excluding appropriate levels or stages of the health problem. If the outcomes were assessed based on existing definitions or diagnostic criteria, then the answer to this question is likely to be yes. If the outcomes were assessed using observer reported, or self-reported scales, the risk of over- or under-reporting is increased, and objectivity is compromised. Importantly, determine if the measurement tools used were validated instruments as this has a significant impact on outcome assessment validity.

**52. Was the condition measured in a standard, reliable way for all participants?**

Considerable judgment is required to determine the presence of some health outcomes. Having established the validity of the outcome measurement instrument (see item 6 of this scale), it is important to establish how the measurement was conducted. Were those involved in collecting data trained or educated in the use of the instrument/s? If there was more than one data collector, were they similar in terms of level of education, clinical or research experience, or level of responsibility in the piece of research being appraised? When there was more than one observer or collector, was there comparison of results from across the observers? Was the condition measured in the same way for all participants?

© JBI, 2020. All rights reserved. JBI grants use of these tools for research purposes only. All other enquiries should be sent to jbisynthesis@adelaide.edu.au.

53. **Was there appropriate statistical analysis?**

Importantly, the numerator and denominator should be clearly reported, and percentages should be given with confidence intervals.  The methods section should be detailed enough for reviewers to identify the analytical technique used and how specific variables were measured. Additionally, it is also important to assess the appropriateness of the analytical strategy in terms of the assumptions associated with the approach as differing methods of analysis are based on differing assumptions about the data and how it will respond.

54. **Was the response rate adequate, and if not, was the low response rate managed appropriately?**

A large number of dropouts, refusals or "not founds" amongst selected subjects may diminish a study's validity, as can a low response rates for survey studies. The authors should clearly discuss the response rate and any reasons for non-response and compare persons in the study to those not in the study, particularly with regards to their socio-demographic characteristics. If reasons for non-response appear to be unrelated to the outcome measured and the characteristics of non-responders are comparable to those who do respond in the study (addressed in question 5, coverage bias), the researchers may be able to justify a more modest response rate.

# JBI CRITICAL APPRAISAL CHECKLIST FOR STUDIES REPORTING PREVALENCE DATA

Reviewer _____________Jamie Brannigan______________Date______02/05/2021________________

Author___________Moran et al_________________Year__2013___ Record Number__1/26____

| | Yes | No | Unclear | Not applicable |
|---|---|---|---|---|
| Was the sample frame appropriate to address the target population?<br>109. | ☒ | ☐ | ☐ | ☐ |
| 110. Were study participants sampled in an appropriate way? | ☒ | ☐ | ☐ | ☐ |
| 111. Was the sample size adequate? | ☐ | ☐ | ☒ | ☐ |

© JBI, 2020. All rights reserved. JBI grants use of these

tools for research purposes only. All other enquiries should be sent to jbisynthesis@adelaide.edu.au.

112. Were the study subjects and the setting described in detail? ☒ ☐ ☐ ☐

113. Was the data analysis conducted with sufficient coverage of the identified sample? ☒ ☐ ☐ ☐

114. Were valid methods used for the identification of the condition? ☒ ☐ ☐ ☐

Was the condition measured in a standard, reliable way for all participants? .
115. ☐ ☒ ☐ ☐

116. Was there appropriate statistical analysis? ☐ ☒ ☐ ☐

117. Was the response rate adequate, and if not, was the low response rate managed appropriately? ☒ ☐ ☐ ☐

Overall appraisal: Include ☒ Exclude ☐ Seek further info ☐

Comments (Including reason for exclusion)

Suitable for narrative review discussion but unsuitable for any quantitative meta-analysis.

© JBI, 2020. All rights reserved. JBI grants use of these tools for research purposes only. All other enquiries should be sent to jbisynthesis@adelaide.edu.au.

# JBI CRITICAL APPRAISAL CHECKLIST FOR STUDIES REPORTING PREVALENCE DATA

*How to cite: Munn Z, Moola S, Lisy K, Riitano D, Tufanaru C. Methodological guidance for systematic reviews of observational epidemiological studies reporting prevalence and incidence data. Int J Evid Based Healthc. 2015;13(3):147–153.*

Answers: Yes, No, Unclear or Not/Applicable

**55. Was the sample frame appropriate to address the target population?**

This question relies upon knowledge of the broader characteristics of the population of interest and the geographical area. If the study is of women with breast cancer, knowledge of at least the characteristics, demographics and medical history is needed. The term "target population" should not be taken to infer every individual from everywhere or with similar disease or exposure characteristics. Instead, give consideration to specific population characteristics in the study, including age range, gender, morbidities, medications, and other potentially influential factors. For example, a sample frame may not be appropriate to address the target population if a certain group has been used (such as those working for one organisation, or one profession) and the results then inferred to the target population (i.e. working adults). A sample frame may be appropriate when it includes almost all the members of the target population (i.e. a census, or a complete list of participants or complete registry data).

**56. Were study participants recruited in an appropriate way?**

Studies may report random sampling from a population, and the methods section should report how sampling was performed. Random probabilistic sampling from a defined subset of the population (sample frame) should be employed in most cases, however, random probabilistic sampling is not needed when everyone in the sampling frame will be included/ analysed. For example, reporting on all the data from a good census is appropriate as a good census will identify everybody. When using cluster sampling, such as a random sample of villages within a region, the methods need to be clearly stated as the precision of the final prevalence estimate incorporates the clustering effect. Convenience samples, such as a street survey or interviewing lots of people at a public gatherings are not considered to provide a representative sample of the base population.

© JBI, 2020. All rights reserved. JBI grants use of these tools for research purposes only. All other enquiries should be sent to jbisynthesis@adelaide.edu.au.

**57. Was the sample size adequate?**

The larger the sample, the narrower will be the confidence interval around the prevalence estimate, making the results more precise. An adequate sample size is important to ensure good precision of the final estimate. Ideally we are looking for evidence that the authors conducted a sample size calculation to determine an adequate sample size. This will estimate how many subjects are needed to produce a reliable estimate of the measure(s) of interest. For conditions with a low prevalence, a larger sample size is needed. Also consider sample sizes for subgroup (or characteristics) analyses, and whether these are appropriate. Sometimes, the study will be large enough (as in large national surveys) whereby a sample size calculation is not required. In these cases, sample size can be considered adequate.

When there is no sample size calculation and it is not a large national survey, the reviewers may consider conducting their own sample size analysis using the following formula: (Naing et al. 2006, Daniel 1999)

n= Z2P(1-P)

d2

Where:

n= sample size

Z = Z statistic for a level of confidence

P = Expected prevalence or proportion (in proportion of one; if 20%, P = 0.2)

d = precision (in proportion of one; if 5%, d=0.05)

**Ref:**

Naing L, Winn T, Rusli BN. Practical issues in calculating the sample size for prevalence studies Archives of Orofacial Sciences. 2006;1:9-14.

Daniel WW. Biostatistics: A Foundation for Analysis in the Health Sciences.

Edition. 7th ed. New York: John Wiley & Sons. 1999.

© JBI, 2020. All rights reserved. JBI grants use of these tools for research purposes only. All other enquiries should be sent to jbisynthesis@adelaide.edu.au.

**58. Were the study subjects and setting described in detail?**

Certain diseases or conditions vary in prevalence across different geographic regions and populations (e.g. Women vs. Men, sociodemographic variables between countries). The study sample should be described in sufficient detail so that other researchers can determine if it is comparable to the population of interest to them.

**59. Was data analysis conducted with sufficient coverage of the identified sample?**

Coverage bias can occur when not all subgroups of the identified sample respond at the same rate. For instance, you may have a very high response rate overall for your study, but the response rate for a certain subgroup (i.e. older adults) may be quite low.

**60. Were valid methods used for the identification of the condition?**

Here we are looking for measurement or classification bias. Many health problems are not easily diagnosed or defined and some measures may not be capable of including or excluding appropriate levels or stages of the health problem. If the outcomes were assessed based on existing definitions or diagnostic criteria, then the answer to this question is likely to be yes. If the outcomes were assessed using observer reported, or self-reported scales, the risk of over- or under-reporting is increased, and objectivity is compromised. Importantly, determine if the measurement tools used were validated instruments as this has a significant impact on outcome assessment validity.

**61. Was the condition measured in a standard, reliable way for all participants?**

Considerable judgment is required to determine the presence of some health outcomes. Having established the validity of the outcome measurement instrument (see item 6 of this scale), it is important to establish how the measurement was conducted. Were those involved in collecting data trained or educated in the use of the instrument/s? If there was more than one data collector, were they similar in terms of level of education, clinical or research experience, or level of responsibility in the piece of research being appraised? When there was more than one observer or collector, was there comparison of results from across the observers? Was the condition measured in the same way for all participants?

© JBI, 2020. All rights reserved. JBI grants use of these tools for research purposes only. All other enquiries should be sent to jbisynthesis@adelaide.edu.au.

62. **Was there appropriate statistical analysis?**

Importantly, the numerator and denominator should be clearly reported, and percentages should be given with confidence intervals. The methods section should be detailed enough for reviewers to identify the analytical technique used and how specific variables were measured. Additionally, it is also important to assess the appropriateness of the analytical strategy in terms of the assumptions associated with the approach as differing methods of analysis are based on differing assumptions about the data and how it will respond.

63. **Was the response rate adequate, and if not, was the low response rate managed appropriately?**

A large number of dropouts, refusals or "not founds" amongst selected subjects may diminish a study's validity, as can a low response rates for survey studies. The authors should clearly discuss the response rate and any reasons for non-response and compare persons in the study to those not in the study, particularly with regards to their socio-demographic characteristics. If reasons for non-response appear to be unrelated to the outcome measured and the characteristics of non-responders are comparable to those who do respond in the study (addressed in question 5, coverage bias), the researchers may be able to justify a more modest response rate.

© JBI, 2020. All rights reserved. JBI grants use of these tools for research purposes only. All other enquiries should be sent to jbisynthesis@adelaide.edu.au.

# JBI CRITICAL APPRAISAL CHECKLIST FOR STUDIES REPORTING PREVALENCE DATA

Reviewer _____________Jamie Brannigan______________Date_______03/05/2021_________________

Author___________Nakanishi et al_________________Year__2019___ Record Number__5/26____

| | Yes | No | Unclear | Not applicable |
|---|---|---|---|---|
| Was the sample frame appropriate to address the target population? 118. | ☒ | ☐ | ☐ | ☐ |
| 119. Were study participants sampled in an appropriate way? | ☒ | ☐ | ☐ | ☐ |
| 120. Was the sample size adequate? | ☐ | ☐ | ☒ | ☐ |
| 121. Were the study subjects and the setting described in detail? | ☒ | ☐ | ☐ | ☐ |
| 122. Was the data analysis conducted with sufficient coverage of the identified sample? | ☒ | ☐ | ☐ | ☐ |
| 123. Were valid methods used for the identification of the condition? | ☐ | ☐ | ☒ | ☐ |
| Was the condition measured in a standard, reliable way for all participants? 124. | ☒ | ☐ | ☐ | ☐ |
| 125. Was there appropriate statistical analysis? | ☒ | ☐ | ☐ | ☐ |
| 126. Was the response rate adequate, and if not, was the low response rate managed appropriately? | ☐ | ☐ | ☐ | ☒ |

Overall appraisal: Include ☒ Exclude ☐ Seek further info ☐

Comments (Including reason for exclusion)

Retrospective assessment of pressure ulcer reporting rather than prospective assessment by investigators. Unsure of reporting guidelines in the hospital and hence if methods for identification were valid.

# JBI CRITICAL APPRAISAL CHECKLIST FOR STUDIES REPORTING PREVALENCE DATA

*How to cite: Munn Z, Moola S, Lisy K, Riitano D, Tufanaru C. Methodological guidance for systematic reviews of observational epidemiological studies reporting prevalence and incidence data. Int J Evid Based Healthc. 2015;13(3):147–153.*

Answers: Yes, No, Unclear or Not/Applicable

**64. Was the sample frame appropriate to address the target population?**

This question relies upon knowledge of the broader characteristics of the population of interest and the geographical area. If the study is of women with breast cancer, knowledge of at least the characteristics, demographics and medical history is needed. The term "target population" should not be taken to infer every individual from everywhere or with similar disease or exposure characteristics. Instead, give consideration to specific population characteristics in the study, including age range, gender, morbidities, medications, and other potentially influential factors. For example, a sample frame may not be appropriate to address the target population if a certain group has been used (such as those working for one organisation, or one profession) and the results then inferred to the target population (i.e. working adults). A sample frame may be appropriate when it includes almost all the members of the target population (i.e. a census, or a complete list of participants or complete registry data).

**65. Were study participants recruited in an appropriate way?**

Studies may report random sampling from a population, and the methods section should report how sampling was performed. Random probabilistic sampling from a defined subset of the population (sample frame) should be employed in most cases, however, random probabilistic sampling is not needed when everyone in the sampling frame will be included/ analysed. For example, reporting on all the data from a good census is appropriate as a good census will identify everybody. When using cluster sampling, such as a random sample of villages within a region, the methods need to be clearly stated as the precision of the final prevalence estimate incorporates the clustering effect. Convenience samples, such as a street survey or interviewing lots of people at a public gatherings are not considered to provide a representative sample of the base population.

© JBI, 2020. All rights reserved. JBI grants use of these tools for research purposes only. All other enquiries should be sent to jbisynthesis@adelaide.edu.au.

## 66. Was the sample size adequate?

The larger the sample, the narrower will be the confidence interval around the prevalence estimate, making the results more precise. An adequate sample size is important to ensure good precision of the final estimate. Ideally we are looking for evidence that the authors conducted a sample size calculation to determine an adequate sample size. This will estimate how many subjects are needed to produce a reliable estimate of the measure(s) of interest. For conditions with a low prevalence, a larger sample size is needed. Also consider sample sizes for subgroup (or characteristics) analyses, and whether these are appropriate. Sometimes, the study will be large enough (as in large national surveys) whereby a sample size calculation is not required. In these cases, sample size can be considered adequate.

When there is no sample size calculation and it is not a large national survey, the reviewers may consider conducting their own sample size analysis using the following formula: (Naing et al. 2006, Daniel 1999)

n= Z2P(1-P)

d2

Where:

n= sample size

Z = Z statistic for a level of confidence

P = Expected prevalence or proportion (in proportion of one; if 20%, P = 0.2)

d = precision (in proportion of one; if 5%, d=0.05)

**Ref:**

Naing L, Winn T, Rusli BN. Practical issues in calculating the sample size for prevalence studies Archives of Orofacial Sciences. 2006;1:9-14.

Daniel WW. Biostatistics: A Foundation for Analysis in the Health Sciences.

Edition. 7th ed. New York: John Wiley & Sons. 1999.

© JBI, 2020. All rights reserved. JBI grants use of these tools for research purposes only. All other enquiries should be sent to jbisynthesis@adelaide.edu.au.

**67. Were the study subjects and setting described in detail?**

Certain diseases or conditions vary in prevalence across different geographic regions and populations (e.g. Women vs. Men, sociodemographic variables between countries). The study sample should be described in sufficient detail so that other researchers can determine if it is comparable to the population of interest to them.

**68. Was data analysis conducted with sufficient coverage of the identified sample?**

Coverage bias can occur when not all subgroups of the identified sample respond at the same rate. For instance, you may have a very high response rate overall for your study, but the response rate for a certain subgroup (i.e. older adults) may be quite low.

**69. Were valid methods used for the identification of the condition?**

Here we are looking for measurement or classification bias. Many health problems are not easily diagnosed or defined and some measures may not be capable of including or excluding appropriate levels or stages of the health problem. If the outcomes were assessed based on existing definitions or diagnostic criteria, then the answer to this question is likely to be yes. If the outcomes were assessed using observer reported, or self-reported scales, the risk of over- or under-reporting is increased, and objectivity is compromised. Importantly, determine if the measurement tools used were validated instruments as this has a significant impact on outcome assessment validity.

**70. Was the condition measured in a standard, reliable way for all participants?**

Considerable judgment is required to determine the presence of some health outcomes. Having established the validity of the outcome measurement instrument (see item 6 of this scale), it is important to establish how the measurement was conducted. Were those involved in collecting data trained or educated in the use of the instrument/s? If there was more than one data collector, were they similar in terms of level of education, clinical or research experience, or level of responsibility in the piece of research being appraised? When there was more than one observer or collector, was there comparison of results from across the observers? Was the condition measured in the same way for all participants?

© JBI, 2020. All rights reserved. JBI grants use of these tools for research purposes only. All other enquiries should be sent to jbisynthesis@adelaide.edu.au.

**71. Was there appropriate statistical analysis?**

Importantly, the numerator and denominator should be clearly reported, and percentages should be given with confidence intervals. The methods section should be detailed enough for reviewers to identify the analytical technique used and how specific variables were measured. Additionally, it is also important to assess the appropriateness of the analytical strategy in terms of the assumptions associated with the approach as differing methods of analysis are based on differing assumptions about the data and how it will respond.

**72. Was the response rate adequate, and if not, was the low response rate managed appropriately?**

A large number of dropouts, refusals or "not founds" amongst selected subjects may diminish a study's validity, as can a low response rates for survey studies. The authors should clearly discuss the response rate and any reasons for non-response and compare persons in the study to those not in the study, particularly with regards to their socio-demographic characteristics. If reasons for non-response appear to be unrelated to the outcome measured and the characteristics of non-responders are comparable to those who do respond in the study (addressed in question 5, coverage bias), the researchers may be able to justify a more modest response rate.

© JBI, 2020. All rights reserved. JBI grants use of these tools for research purposes only. All other enquiries should be sent to jbisynthesis@adelaide.edu.au.

# JBI CRITICAL APPRAISAL CHECKLIST FOR SYSTEMATIC REVIEWS AND RESEARCH SYNTHESES

Reviewer _________Jamie Brannigan________________Date________03/05/2021_______________

Author_______Peck et al______________________Year___2018___ Record Number_21/26____

| | Yes | No | Unclear | Not applicable |
|---|---|---|---|---|
| 127. Is the review question clearly and explicitly stated? | ☒ | ☐ | ☐ | ☐ |
| 128. Were the inclusion criteria appropriate for the review question? | ☒ | ☐ | ☐ | ☐ |
| 129. Was the search strategy appropriate? | ☒ | ☐ | ☐ | ☐ |
| 130. Were the sources and resources used to search for studies adequate? | ☒ | ☐ | ☐ | ☐ |
| 131. Were the criteria for appraising studies appropriate? | ☐ | ☐ | ☒ | ☐ |
| 132. Was critical appraisal conducted by two or more reviewers independently? | ☐ | ☐ | ☒ | ☐ |
| 133. Were there methods to minimize errors in data extraction? | ☐ | ☐ | ☒ | ☐ |
| 134. Were the methods used to combine studies appropriate? | ☐ | ☐ | ☐ | ☒ |
| 135. Was the likelihood of publication bias assessed? | ☐ | ☐ | ☒ | ☐ |
| 136. Were recommendations for policy and/or practice supported by the reported data? | ☒ | ☐ | ☐ | ☐ |
| 137. Were the specific directives for new research appropriate? | ☒ | ☐ | ☐ | ☐ |

Overall appraisal: Include ☒ Exclude ☐ Seek further info ☐

Comments (Including reason for exclusion)

This review was not a true systematic. Limited methodology discussed. It seem that there was only a second reviewer for title/abstract screening.

# JBI CRITICAL APPRAISAL CHECKLIST FOR SYSTEMATIC REVIEWS AND RESEARCH SYNTHESIS

How to cite: *Aromataris E, Fernandez R, Godfrey C, Holly C, Kahlil H, Tungpunkom P. Summarizing systematic reviews: methodological development, conduct and reporting of an Umbrella review approach. Int J Evid Based Healthc. 2015;13(3):132-40.*

When conducting an umbrella review using the JBI method, the critical appraisal instrument for Systematic Reviews should be used.

The primary and secondary reviewer should discuss each item in the appraisal instrument for each study included in their review. In particular, discussions should focus on what is considered acceptable to the aims of the review in terms of the specific study characteristics. When appraising systematic reviews this discussion may include issues such as what represents an adequate search strategy or appropriate methods of synthesis. The reviewers should be clear on what constitutes acceptable levels of information to allocate a positive appraisal compared with a negative, or response of "unclear". This discussion should ideally take place before the reviewers independently conduct the appraisal.

Within umbrella reviews, quantitative or qualitative systematic reviews may be incorporated, as well as meta-analyses of existing research. There are 11 questions to guide the appraisal of systematic reviews or meta-analyses. Each question should be answered as "yes", "no", or "unclear". Not applicable "NA" is also provided as an option and may be appropriate in rare instances.

### 23. Is the review question clearly and explicitly stated?

The review question is an essential step in the systematic review process. A well-articulated question defines the scope of the review and aids in the development of the search strategy to locate the relevant evidence. An explicitly stated question, formulated around its PICO (Population, Intervention, Comparator, Outcome) elements aids both the review team in the conduct of the review and the reader in determining if the review has achieved its objectives. Ideally the review question should be articulated in a published protocol; however this will not always be the case with many reviews that are located.

### 24. Were the inclusion criteria appropriate for the review question?

The inclusion criteria should be identifiable from, and match the review question. The necessary elements of the PICO should be explicit and clearly defined. The inclusion criteria should be detailed and the included reviews should clearly be eligible when matched against the stated inclusion criteria. Appraisers of meta-analyses will find that inclusion criteria may include criteria around the ability to conduct statistical analyses which would not be the norm for a systematic review. The types of included studies should be relevant to the review question, for example, an umbrella review aiming to summarize a range of effective non-pharmacological interventions for aggressive behaviors amongst elderly patients with dementia will limit itself to including systematic reviews and meta-analyses that synthesize quantitative studies assessing the various interventions; qualitative or economic reviews would not be included.

### 25. Was the search strategy appropriate?

A systematic review should provide evidence of the search strategy that has been used to locate the evidence. This may be found in the methods section of the review report in some cases, or as an appendix that may be provided as supplementary information to the review publication. A systematic review should present a clear search strategy that addresses each of the identifiable PICO components of the review question. Some reviews may also provide a description of the approach to searching and how the terms that were ultimately used were derived, though due to limits on word counts in journals this may be more the norm in online only publications. There

© JBI, 2020. All rights reserved. JBI grants use of these tools for research purposes only. All other enquiries should be sent to jbisynthesis@adelaide.edu.au.

should be evidence of logical and relevant keywords and terms and also evidence that Subject

Headings and Indexing terms have been used in the conduct of the search. Limits on the search should also be considered and their potential impact; for example, if a date limit was used, was this appropriate and/or justified? If only English language studies were included, will such a language bias have an impact on the review? The response to these considerations will depend, in part, on the review question.

## 26. Were the sources and resources used to search for studies adequate?

A systematic review should attempt to identify "all" the available evidence and as such there should be evidence of a comprehensive search strategy. Multiple electronic databases should be searched including major bibliographic citation databases such as MEDLINE and CINAHL. Ideally, other databases that are relevant to the review question should also be searched, for example, a systematic review with a question about a physical therapy intervention should also look to search the PEDro database, whilst a review focusing on an educational intervention should also search the ERIC. Reviews of effectiveness should aim to search trial registries. A comprehensive search is the ideal way to minimize publication bias, as a result, a well conducted systematic review should also attempt to search for grey literature, or "unpublished" studies; this may involve searching websites relevant to the review question, or thesis repositories.

## 27. Were the criteria for appraising studies appropriate?

The systematic review should present a clear statement that critical appraisal was conducted and provide the details of the items that were used to assess the included studies. This may be presented in the methods of the review, as an appendix of supplementary information, or as a reference to a source that can be located. The tools or instruments used should be appropriate for the review question asked and the type of research conducted. For example, a systematic review of effectiveness should present a tool or instrument that addresses aspects of validity for experimental studies and randomized controlled trials such as randomization and blinding – if the review includes observational research to answer the same question a different tool would be more appropriate. Similarly, a review assessing diagnostic test accuracy may refer to the recognized QUADAS[1] tool.

## 28. Was critical appraisal conducted by two or more reviewers independently?

Critical appraisal or some similar assessment of the quality of the literature included in a systematic review is essential. A key characteristic to minimize bias or systematic error in the conduct of a systematic review is to have the critical appraisal of the included studies completed independently and in duplicate by members of the review team. The systematic review should present a clear statement that critical appraisal was conducted by at least two reviewers working independently from each other and conferring where necessary to reach decision regarding study quality and eligibility on the basis of quality.

## 29. Were there methods to minimize errors in data extraction?

Efforts made by review authors during data extraction can also minimize bias or systematic errors in the conduct of a systematic review. Strategies to minimize bias may include conducting all data extraction in duplicate and independently, using specific tools or instruments to guide data extraction and some evidence of piloting or training around their use.

## 30. Were the methods used to combine studies appropriate?

© JBI, 2020. All rights reserved. JBI grants use of these tools for research purposes only. All other enquiries should be sent to jbisynthesis@adelaide.edu.au.

A synthesis of the evidence is a key feature of a systematic review. The synthesis that is presented should be appropriate for the review question and the stated type of systematic review and evidence it refers to. If a meta-analysis has been conducted this needs to be reviewed carefully.

Was it appropriate to combine the studies? Have the reviewers assessed heterogeneity statistically and provided some explanation for heterogeneity that may be present? Often, where heterogeneous studies are included in the systematic review, narrative synthesis will be an appropriate method for presenting the results of multiple studies. If a qualitative review, are the methods that have been used to synthesize findings congruent with the stated methodology of the review? Is there adequate descriptive and explanatory information to support the final synthesized findings that have been constructed from the findings sourced from the original research?

### 31. Was the likelihood of publication bias assessed?

As mentioned, a comprehensive search strategy is the best means by which a review author may alleviate the impact of publication bias on the results of the review. Reviews may also present statistical tests such as Egger's test or funnel plots to also assess the potential presence of publication bias and its potential impact on the results of the review. This question will not be applicable to systematic reviews of qualitative evidence.

### 32. Were recommendations for policy and/or practice supported by the reported data?

Whilst the first nine (9) questions specifically look to identify potential bias in the conduct of a systematic review, the final questions are more indictors of review quality rather than validity. Ideally a review should present recommendations for policy and practice. Where these recommendations are made there should be a clear link to the results of the review. Is there evidence that the strength of the findings and the quality of the research been considered in the formulation of review recommendations?

### 33. Were the specific directives for new research appropriate?

The systematic review process is recognized for its ability to identify where gaps in the research, or knowledge base, around a particular topic exist. Most systematic review authors will provide some indication, often in the discussion section of the report, of where future research direction should lie. Where evidence is scarce or sample sizes that support overall estimates of effect are small and effect estimates are imprecise, repeating similar research to those identified by the review may be necessary and appropriate. In other instances, the case for new research questions to investigate the topic may be warranted.

## REFERENCES

3. Whiting P, Rutjes AWS, Reitsma JB, Bossuyt PMM, Kleijnen J. The development of QUADAS: a tool for the quality assessment of studies of diagnostic accuracy included in systematic reviews. BMC Medical Research Methodology. 2003;3:25 doi:10.1186/1471-2288-3-25.

© JBI, 2020. All rights reserved. JBI grants use of these tools for research purposes only. All other enquiries should be sent to jbisynthesis@adelaide.edu.au.

# JBI Critical Appraisal Checklist for Case Series

Reviewer _____Jamie Brannigan_______________________Date_____03/05/2021___________________

Author________Powers______________________________Year___1997__ Record Number__16/26___

| | Yes | No | Unclear | Not applicable |
|---|---|---|---|---|
| Were there clear criteria for inclusion in the case series? | ☐ | ☒ | ☐ | ☐ |
| Was the condition measured in a standard, reliable way for all participants included in the case series? | ☐ | ☐ | ☒ | ☐ |
| Were valid methods used for identification of the condition for all participants included in the case series? | ☐ | ☒ | ☐ | ☐ |
| Did the case series have consecutive inclusion of participants? | ☒ | ☐ | ☐ | ☐ |
| Did the case series have complete inclusion of participants? | ☒ | ☐ | ☐ | ☐ |
| Was there clear reporting of the demographics of the participants in the study? | ☐ | ☐ | ☒ | ☐ |
| Was there clear reporting of clinical information of the participants? | ☐ | ☒ | ☐ | ☐ |
| Were the outcomes or follow up results of cases clearly reported? | ☐ | ☒ | ☐ | ☐ |
| Was there clear reporting of the presenting site(s)/clinic(s) demographic information? | ☒ | ☐ | ☐ | ☐ |
| Was statistical analysis appropriate? | ☒ | ☐ | ☐ | ☐ |

Overall appraisal: Include ☒ Exclude ☐ Seek further info ☐

Comments (Including reason for exclusion)

A mixture of case series and QI work. Non-consecutive, non-complete inclusion in the first year, but on commencement of study this is amended. Very little clinical information regarding individual patients, with greater focus on the QI work.

# INTRODUCTION TO THE CASE SERIES CRITICAL APPRAISAL TOOL

*How to cite:* Munn Z, Barker T, Moola S, Tufanaru C, Stern C, McArthur A, Stephenson M, Aromataris E. Methodological quality of case series studies, JBI Evidence Synthesis, doi: 10.11124/JBISRIR-D-19-00099

The definition of a case series varies across the medical literature, which has resulted in inconsistent use of this term (Appendix 1).[1-3] The gamut of case studies is wide, with some studies claiming to be a case series realistically being nothing more than a collection of case reports, with others more akin to cohort studies or even quasi-experimental before and after studies. This has created difficulty in assigning 'case series' a position in the hierarchy of evidence and identifying and appropriate critical appraisal tool.[1, 2]

Dekkers et al. define a case series as a study in which 'only patients with the outcome are sampled (either those who have an exposure or those who are selected without regard to exposure), which does not permit calculation of an absolute risk.'[1p.39] The outcome could be a disease or a disease related outcome. This is contrasted to cohort studies where sampling is based on exposure (or characteristic), and case- control studies where there is a comparison group without the disease.

The completeness of a case series contributes to its reliability.[1] Studies that indicate a consecutive and complete inclusion are more reliable than those that do not. For example, a case series that states 'we included all patients (24) with osteosarcoma who presented to our clinic between March 2005 and June 2006' is more reliable than a study that simply states 'we report a case series of 24 people with osteosarcoma.'

For the purposes of this checklist, we agree with the principles outlined in the Dekker et al. paper, and define case series as studies where only patients with a certain disease or disease-related outcome are sampled. Some of the items below relate to risk of bias, whilst others relate to ensuring adequate reporting and statistical analysis. A response of 'no' to any of the questions below negatively impacts the quality of a case series.

© JBI, 2020. All rights reserved. JBI grants use of these tools for research purposes only. All other enquiries should be sent to jbisynthesis@adelaide.edu.au.

# TOOL GUIDANCE

Answers: Yes, No, Unclear or Not/Applicable

1. **Were there clear criteria for inclusion in the case series?**

   The authors should provide clear inclusion (and exclusion criteria where appropriate) for the study participants. The inclusion/exclusion criteria should be specified (e.g., risk, stage of disease progression) with sufficient detail and all the necessary information critical to the study.

2. **Was the condition measured in a standard, reliable way for all participants included in the case series?**

   The study should clearly describe the method of measurement of the condition. This should be done in a standard (i.e. same way for all patients) and reliable (i.e. repeatable and reproducible results) way.

3. **Were valid methods used for identification of the condition for all participants included in the case series?**

   Many health problems are not easily diagnosed or defined and some measures may not be capable of including or excluding appropriate levels or stages of the health problem. If the outcomes were assessed based on existing definitions or diagnostic criteria, then the answer to this question is likely to be yes. If the outcomes were assessed using observer reported, or self-reported scales, the risk of over- or under-reporting is increased, and objectivity is compromised. Importantly, determine if the measurement tools used were validated instruments as this has a significant impact on outcome assessment validity.

4. **Did the case series have consecutive inclusion of participants?**

   Studies that indicate a consecutive inclusion are more reliable than those that do not. For example, a case series that states ‘we included all patients (24) with osteosarcoma who presented to our clinic between March 2005 and June 2006’ is more reliable than a study that simply states ‘we report a case series of 24 people with osteosarcoma.’

5. **Did the case series have complete inclusion of participants?**

   The completeness of a case series contributes to its reliability (1). Studies that indicate a complete inclusion are more reliable than those that do not. A stated above, a case series that states ‘we included all patients (24) with osteosarcoma who presented to our clinic between March 2005 and June 2006’ is more reliable than a study that simply states ‘we report a case series of 24 people with osteosarcoma.’

6. **Was there clear reporting of the demographics of the participants in the study?**

© JBI, 2020. All rights reserved. JBI grants use of these tools for research purposes only. All other enquiries should be sent to jbisynthesis@adelaide.edu.au.

The case series should clearly describe relevant participant's demographics such as the following information where relevant: participant's age, sex, education, geographic region, ethnicity, time period, education.

## 7. Was there clear reporting of clinical information of the participants?

There should be clear reporting of clinical information of the participants such as the following information where relevant: disease status, comorbidities, stage of disease, previous interventions/treatment, results of diagnostic tests, etc.

## 8. Were the outcomes or follow-up results of cases clearly reported?

The results of any intervention or treatment should be clearly reported in the case series. A good case study should clearly describe the clinical condition post-intervention in terms of the presence or lack of symptoms. The outcomes of management/treatment when presented as images or figures can help in conveying the information to the reader/clinician. It is important that adverse events are clearly documented and described, particularly a new or unique condition is being treated or when a new drug or treatment is used. In addition, unanticipated events, if any that may yield new or useful information should be identified and clearly described.

## 9. Was there clear reporting of the presenting site(s)/clinic(s) demographic information?

Certain diseases or conditions vary in prevalence across different geographic regions and populations (e.g. women vs. men, sociodemographic variables between countries). The study sample should be described in sufficient detail so that other researchers can determine if it is comparable to the population of interest to them.

## 10. Was statistical analysis appropriate?

As with any consideration of statistical analysis, consideration should be given to whether there was a more appropriate alternate statistical method that could have been used. The methods section of studies should be detailed enough for reviewers to identify which analytical techniques were used and whether these were suitable.

© JBI, 2020. All rights reserved. JBI grants use of these tools for research purposes only. All other enquiries should be sent to jbisynthesis@adelaide.edu.au.

# REFERENCES

1. Dekkers OM, Egger M, Altman DG, Vandenbroucke JP. Distinguishing case series from cohort studies. Annals of Internal Medicine. 2012;156(1 Part 1):37-40.

2. Esene IN, Ngu J, El Zoghby M, Solaroglu I, Sikod AM, Kotb A et al. Case series and descriptive cohort studies in neurosurgery: the confusion and solution. Child's Nervous System. 2014;30(8):1321-32.

3. Abu-Zidan FM, Abbas AK, Hefny AF. Clinical “case series”: a concept analysis. African Health Sciences. 2012;12(4):557-62.

4 Straus SE, Richardson WS, Glasziou P, Haynes RB. Evidence-based medicine: How to practice and teach EBM. 3rd Edition ed: Elsevier 2005.

## Appendix 1: Case series definitions:

‘A report on a series of patients with an outcome of interest. No control group is involved.’(4) (p 279)

‘A case series is a descriptive study involving a group of patients who all have the same disease or condition: the aim is to describe common and differing characteristics of a particular group of individuals’ (Oxford Handbook of medical statistics)

‘A group or series of case reports involving patients who were given similar treatment. Reports of case series usually contain detailed information about the individual patients. This includes demographic information (for example, age, gender, ethnic origin) and information on diagnosis, treatment, response to treatment, and follow-up after treatment.’ Law K, Howick J. OCEBM Table of Evidence Glossary. 2013 [cited 2014 10th January]; Available from: http://www.cebm.net/index.aspx?o=1116

‘A **case series** (also known as a clinical **series**) is a type of medical research study that tracks subjects with a known exposure, such as patients who have received a similar treatment, or examines their medical records for exposure and outcome.’ Wikipedia

‘A study which makes observations on a series of individuals, usually all receiving the same intervention, with no control group. Comments: At this stage it is unclear whether case series should be included in Cochrane systematic reviews, but we have left them in the list so that working groups can consider whether there are circumstances in which it would be appropriate to include them, and to assess risk of bias. A particular reason for including case series might be where they provide evidence relating to adverse effects of an intervention. Potential examples of risk of bias might be that if a case series does not [attempt to] recruit consecutive participants, this might introduce a risk of selection bias, while some case series could be at risk of detection bias, if the circumstances in which adverse effects are reported (or elicited) are not standardised.’ http://bmg.cochrane.org/research-projectscochrane-risk-bias-tool

© JBI, 2020. All rights reserved. JBI grants use of these tools for research purposes only. All other enquiries should be sent to jbisynthesis@adelaide.edu.au.

# JBI CRITICAL APPRAISAL CHECKLIST FOR STUDIES REPORTING PREVALENCE DATA

Reviewer _____________Jamie Brannigan______________Date______03/05/2021_________________

Author__________Powers et al_________________Year__2006___ Record Number__3/26____

| | Yes | No | Unclear | Not applicable |
|---|---|---|---|---|
| 138. Was the sample frame appropriate to address the target population? | ☐ | ☐ | ☒ | ☐ |
| 139. Were study participants sampled in an appropriate way? | ☒ | ☐ | ☐ | ☐ |
| 140. Was the sample size adequate? | ☐ | ☐ | ☒ | ☐ |
| 141. Were the study subjects and the setting described in detail? | ☒ | ☐ | ☐ | ☐ |
| 142. Was the data analysis conducted with sufficient coverage of the identified sample? | ☒ | ☐ | ☐ | ☐ |
| 143. Were valid methods used for the identification of the condition? | ☒ | ☐ | ☐ | ☐ |
| 144. Was the condition measured in a standard, reliable way for all participants? | ☒ | ☐ | ☐ | ☐ |
| 145. Was there appropriate statistical analysis? | ☒ | ☐ | ☐ | ☐ |
| 146. Was the response rate adequate, and if not, was the low response rate managed appropriately? | ☐ | ☐ | ☐ | ☒ |

Overall appraisal: Include ☒ Exclude ☐ Seek further info ☐

Comments (Including reason for exclusion)

Suitable for quantitative meta-analysis. Appropriateness of sample frame unclear as exclusively a critical care population.

# JBI CRITICAL APPRAISAL CHECKLIST FOR STUDIES REPORTING PREVALENCE DATA

*How to cite: Munn Z, Moola S, Lisy K, Riitano D, Tufanaru C. Methodological guidance for systematic reviews of observational epidemiological studies reporting prevalence and incidence data. Int J Evid Based Healthc. 2015;13(3):147–153.*

Answers: Yes, No, Unclear or Not/Applicable

**73. Was the sample frame appropriate to address the target population?**

This question relies upon knowledge of the broader characteristics of the population of interest and the geographical area. If the study is of women with breast cancer, knowledge of at least the characteristics, demographics and medical history is needed. The term "target population" should not be taken to infer every individual from everywhere or with similar disease or exposure characteristics. Instead, give consideration to specific population characteristics in the study, including age range, gender, morbidities, medications, and other potentially influential factors. For example, a sample frame may not be appropriate to address the target population if a certain group has been used (such as those working for one organisation, or one profession) and the results then inferred to the target population (i.e. working adults). A sample frame may be appropriate when it includes almost all the members of the target population (i.e. a census, or a complete list of participants or complete registry data).

**74. Were study participants recruited in an appropriate way?**

Studies may report random sampling from a population, and the methods section should report how sampling was performed. Random probabilistic sampling from a defined subset of the population (sample frame) should be employed in most cases, however, random probabilistic sampling is not needed when everyone in the sampling frame will be included/ analysed. For example, reporting on all the data from a good census is appropriate as a good census will identify everybody. When using cluster sampling, such as a random sample of villages within a region, the methods need to be clearly stated as the precision of the final prevalence estimate incorporates the clustering effect. Convenience samples, such as a street survey or interviewing lots of people at a public gatherings are not considered to provide a representative sample of the base population.

© JBI, 2020. All rights reserved. JBI grants use of these tools for research purposes only. All other enquiries should be sent to jbisynthesis@adelaide.edu.au.

## 75. Was the sample size adequate?

The larger the sample, the narrower will be the confidence interval around the prevalence estimate, making the results more precise. An adequate sample size is important to ensure good precision of the final estimate. Ideally we are looking for evidence that the authors conducted a sample size calculation to determine an adequate sample size. This will estimate how many subjects are needed to produce a reliable estimate of the measure(s) of interest. For conditions with a low prevalence, a larger sample size is needed. Also consider sample sizes for subgroup (or characteristics) analyses, and whether these are appropriate. Sometimes, the study will be large enough (as in large national surveys) whereby a sample size calculation is not required. In these cases, sample size can be considered adequate.

When there is no sample size calculation and it is not a large national survey, the reviewers may consider conducting their own sample size analysis using the following formula: (Naing et al. 2006, Daniel 1999)

n= Z2P(1-P)

d2

Where:

n= sample size

Z = Z statistic for a level of confidence

P = Expected prevalence or proportion (in proportion of one; if 20%, P = 0.2)

d = precision (in proportion of one; if 5%, d=0.05)

**Ref:**

Naing L, Winn T, Rusli BN. Practical issues in calculating the sample size for prevalence studies Archives of Orofacial Sciences. 2006;1:9-14.

Daniel WW. Biostatistics: A Foundation for Analysis in the Health Sciences.

Edition. 7th ed. New York: John Wiley & Sons. 1999.

© JBI, 2020. All rights reserved. JBI grants use of these tools for research purposes only. All other enquiries should be sent to jbisynthesis@adelaide.edu.au.

76. **Were the study subjects and setting described in detail?**

Certain diseases or conditions vary in prevalence across different geographic regions and populations (e.g. Women vs. Men, sociodemographic variables between countries). The study sample should be described in sufficient detail so that other researchers can determine if it is comparable to the population of interest to them.

77. **Was data analysis conducted with sufficient coverage of the identified sample?**

Coverage bias can occur when not all subgroups of the identified sample respond at the same rate. For instance, you may have a very high response rate overall for your study, but the response rate for a certain subgroup (i.e. older adults) may be quite low.

78. **Were valid methods used for the identification of the condition?**

Here we are looking for measurement or classification bias. Many health problems are not easily diagnosed or defined and some measures may not be capable of including or excluding appropriate levels or stages of the health problem. If the outcomes were assessed based on existing definitions or diagnostic criteria, then the answer to this question is likely to be yes. If the outcomes were assessed using observer reported, or self-reported scales, the risk of over- or under-reporting is increased, and objectivity is compromised. Importantly, determine if the measurement tools used were validated instruments as this has a significant impact on outcome assessment validity.

79. **Was the condition measured in a standard, reliable way for all participants?**

Considerable judgment is required to determine the presence of some health outcomes. Having established the validity of the outcome measurement instrument (see item 6 of this scale), it is important to establish how the measurement was conducted. Were those involved in collecting data trained or educated in the use of the instrument/s? If there was more than one data collector, were they similar in terms of level of education, clinical or research experience, or level of responsibility in the piece of research being appraised? When there was more than one observer or collector, was there comparison of results from across the observers? Was the condition measured in the same way for all participants?

© JBI, 2020. All rights reserved. JBI grants use of these tools for research purposes only. All other enquiries should be sent to jbisynthesis@adelaide.edu.au.

**80. Was there appropriate statistical analysis?**

Importantly, the numerator and denominator should be clearly reported, and percentages should be given with confidence intervals.  The methods section should be detailed enough for reviewers to identify the analytical technique used and how specific variables were measured. Additionally, it is also important to assess the appropriateness of the analytical strategy in terms of the assumptions associated with the approach as differing methods of analysis are based on differing assumptions about the data and how it will respond.

**81. Was the response rate adequate, and if not, was the low response rate managed appropriately?**

A large number of dropouts, refusals or "not founds" amongst selected subjects may diminish a study's validity, as can a low response rates for survey studies. The authors should clearly discuss the response rate and any reasons for non-response and compare persons in the study to those not in the study, particularly with regards to their socio-demographic characteristics. If reasons for non-response appear to be unrelated to the outcome measured and the characteristics of non-responders are comparable to those who do respond in the study (addressed in question 5, coverage bias), the researchers may be able to justify a more modest response rate.

© JBI, 2020. All rights reserved. JBI grants use of these tools for research purposes only. All other enquiries should be sent to jbisynthesis@adelaide.edu.au.

# JBI CRITICAL APPRAISAL CHECKLIST FOR CASE REPORTS

Reviewer _____Jamie Brannigan_____________________________ Date______03/05/2021_______________

Author________Rodgers and Rodgers________________ Year__1995___ Record Number___13/26______

| | Yes | No | Unclear | Not applicable |
|---|---|---|---|---|
| 147. Were patient's demographic characteristics clearly described? | ☐ | ☐ | ☒ | ☐ |
| 148. Was the patient's history clearly described and presented as a timeline? | ☒ | ☐ | ☐ | ☐ |
| 149. Was the current clinical condition of the patient on presentation clearly described? | ☒ | ☐ | ☐ | ☐ |
| 150. Were diagnostic tests or assessment methods and the results clearly described? | ☒ | ☐ | ☐ | ☐ |
| 151. Was the intervention(s) or treatment procedure(s) clearly described? | ☒ | ☐ | ☐ | ☐ |
| 152. Was the post-intervention clinical condition clearly described? | ☒ | ☐ | ☐ | ☐ |
| 153. Were adverse events (harms) or unanticipated events identified and described? | ☒ | ☐ | ☐ | ☐ |
| 154. Does the case report provide takeaway lessons? | ☒ | ☐ | ☐ | ☐ |

Overall appraisal: Include ☒ Exclude ☐ Seek further info ☐

Comments (Including reason for exclusion)

Regarding patient's demographics, only age, gender and a single pre-existing condition were disclosed.

# EXPLANATION OF CASE REPORTS CRITICAL APPRAISAL

*How to cite: Moola S, Munn Z, Tufanaru C, Aromataris E, Sears K, Sfetcu R, Currie M, Qureshi R, Mattis P, Lisy K, Mu P-F. Chapter 7: Systematic reviews of etiology and risk. In: Aromataris E, Munn Z (Editors). JBI Manual for Evidence Synthesis.* JBI, 2020. Available from https://synthesismanual.jbi.global

**Case Reports Critical Appraisal Tool**

Answers: Yes, No, Unclear or Not/Applicable

9. **Were patient's demographic characteristics clearly described?**

   Does the case report clearly describe patient's age, sex, race, medical history, diagnosis, prognosis, previous treatments, past and current diagnostic test results, and medications? The setting and context may also be described.

10. **Was the patient's history clearly described and presented as a timeline?**

    A good case report will clearly describe the history of the patient, their medical, family and psychosocial history including relevant genetic information, as well as relevant past interventions and their outcomes. (CARE Checklist 2013)

11. **Was the current clinical condition of the patient on presentation clearly described?**

    The current clinical condition of the patient should be described in detail including the uniqueness of the condition/disease, symptoms, frequency and severity. The case report should also be able to present whether differential diagnoses was considered.

12. **Were diagnostic tests or methods and the results clearly described?**

    A reader of the case report should be provided sufficient information to understand how the patient was assessed. It is important that all appropriate tests are ordered to confirm a diagnosis and therefore the case report should provide a clear description of various diagnostic tests used (whether a gold standard or alternative diagnostic tests). Photographs or illustrations of diagnostic procedures, radiographs, or treatment procedures are usually presented when appropriate to convey a clear message to readers.

13. **Was the intervention(s) or treatment procedure(s) clearly described?**

    It is important to clearly describe treatment or intervention procedures as other clinicians will be reading the paper and therefore may enable clear understanding of the treatment protocol. The report should describe the treatment/intervention protocol in detail; for e.g. in pharmacological management of dental anxiety - the type of drug, route of administration, drug dosage and frequency, and any side effects.

14. **Was the post-intervention clinical condition clearly described?**

    A good case report should clearly describe the clinical condition post-intervention in terms of the presence or lack thereof symptoms. The outcomes of management/treatment when presented as images or figures would help in conveying the information to the reader/clinician.

15. **Were adverse events (harms) or unanticipated events identified and described?**

    With any treatment/intervention/drug, there are bound to be some adverse events and in some cases, they may be severe. It is important that adverse events are clearly documented and described, particularly when a new or unique condition is being treated or when a new drug or

© JBI, 2020. All rights reserved. JBI grants use of these tools for research purposes only. All other enquiries should be sent to jbisynthesis@adelaide.edu.au.

treatment is used. In addition, unanticipated events, if any that may yield new or useful information should be identified and clearly described.

### 16. Does the case report provide takeaway lessons?

Case reports should summarize key lessons learned from a case in terms of the background of the condition/disease and clinical practice guidance for clinicians when presented with similar cases.

## REFERENCES:

Gagnier JJ, Kienle G, Altman DG, Moher D, Sox H, Riley D, CARE Group. The CARE Guidelines: Consensus-Based Clinical Case Reporting Guideline Development. Headache: The Journal of Head and Face Pain, 2013;53(10):1541-1547.

© JBI, 2020. All rights reserved. JBI grants use of these tools for research purposes only. All other enquiries should be sent to jbisynthesis@adelaide.edu.au.

# JBI CRITICAL APPRAISAL CHECKLIST FOR STUDIES REPORTING PREVALENCE DATA

Reviewer _____________Jamie Brannigan______________Date_______03/05/2021_________________

Author___________Theofano et al_________________Year__2015___ Record Number__10/26____

| | Yes | No | Unclear | Not applicable |
|---|---|---|---|---|
| Was the sample frame appropriate to address the target population? 155. | ☒ | ☐ | ☐ | ☐ |
| 156. Were study participants sampled in an appropriate way? | ☒ | ☐ | ☐ | ☐ |
| 157. Was the sample size adequate? | ☐ | ☐ | ☒ | ☐ |
| 158. Were the study subjects and the setting described in detail? | ☒ | ☐ | ☐ | ☐ |
| 159. Was the data analysis conducted with sufficient coverage of the identified sample? | ☐ | ☐ | ☒ | ☐ |
| 160. Were valid methods used for the identification of the condition? | ☐ | ☐ | ☒ | ☐ |
| Was the condition measured in a standard, reliable way for all participants? 161. | ☒ | ☐ | ☐ | ☐ |
| 162. Was there appropriate statistical analysis? | ☐ | ☒ | ☐ | ☐ |
| 163. Was the response rate adequate, and if not, was the low response rate managed appropriately? | ☐ | ☐ | ☐ | ☒ |

Overall appraisal: Include ☐ Exclude ☒ Seek further info ☐

Comments (Including reason for exclusion)

Very brief text. High attrition rate, but characteristics of patients lost to follow up was not disclosed. Retrospective assessment of care records brings validity of identification into question. Very minimal statistical analysis performed.

# JBI CRITICAL APPRAISAL CHECKLIST FOR STUDIES REPORTING PREVALENCE DATA

*How to cite: Munn Z, Moola S, Lisy K, Riitano D, Tufanaru C. Methodological guidance for systematic reviews of observational epidemiological studies reporting prevalence and incidence data. Int J Evid Based Healthc. 2015;13(3):147–153.*

Answers: Yes, No, Unclear or Not/Applicable

**82. Was the sample frame appropriate to address the target population?**

This question relies upon knowledge of the broader characteristics of the population of interest and the geographical area. If the study is of women with breast cancer, knowledge of at least the characteristics, demographics and medical history is needed. The term “target population” should not be taken to infer every individual from everywhere or with similar disease or exposure characteristics. Instead, give consideration to specific population characteristics in the study, including age range, gender, morbidities, medications, and other potentially influential factors. For example, a sample frame may not be appropriate to address the target population if a certain group has been used (such as those working for one organisation, or one profession) and the results then inferred to the target population (i.e. working adults). A sample frame may be appropriate when it includes almost all the members of the target population (i.e. a census, or a complete list of participants or complete registry data).

**83. Were study participants recruited in an appropriate way?**

Studies may report random sampling from a population, and the methods section should report how sampling was performed. Random probabilistic sampling from a defined subset of the population (sample frame) should be employed in most cases, however, random probabilistic sampling is not needed when everyone in the sampling frame will be included/ analysed. For example, reporting on all the data from a good census is appropriate as a good census will identify everybody. When using cluster sampling, such as a random sample of villages within a region, the methods need to be clearly stated as the precision of the final prevalence estimate incorporates the clustering effect. Convenience samples, such as a street survey or interviewing lots of people at a public gatherings are not considered to provide a representative sample of the base population.

© JBI, 2020. All rights reserved. JBI grants use of these tools for research purposes only. All other enquiries should be sent to jbisynthesis@adelaide.edu.au.

**84. Was the sample size adequate?**

The larger the sample, the narrower will be the confidence interval around the prevalence estimate, making the results more precise. An adequate sample size is important to ensure good precision of the final estimate. Ideally we are looking for evidence that the authors conducted a sample size calculation to determine an adequate sample size. This will estimate how many subjects are needed to produce a reliable estimate of the measure(s) of interest. For conditions with a low prevalence, a larger sample size is needed. Also consider sample sizes for subgroup (or characteristics) analyses, and whether these are appropriate. Sometimes, the study will be large enough (as in large national surveys) whereby a sample size calculation is not required. In these cases, sample size can be considered adequate.

When there is no sample size calculation and it is not a large national survey, the reviewers may consider conducting their own sample size analysis using the following formula: (Naing et al. 2006, Daniel 1999)

n= Z2P(1-P)

d2

Where:

n= sample size

Z = Z statistic for a level of confidence

P = Expected prevalence or proportion (in proportion of one; if 20%, P = 0.2)

d = precision (in proportion of one; if 5%, d=0.05)

**Ref:**

Naing L, Winn T, Rusli BN. Practical issues in calculating the sample size for prevalence studies Archives of Orofacial Sciences. 2006;1:9-14.

Daniel WW. Biostatistics: A Foundation for Analysis in the Health Sciences.

Edition. 7th ed. New York: John Wiley & Sons. 1999.

© JBI, 2020. All rights reserved. JBI grants use of these tools for research purposes only. All other enquiries should be sent to jbisynthesis@adelaide.edu.au.

**85. Were the study subjects and setting described in detail?**

Certain diseases or conditions vary in prevalence across different geographic regions and populations (e.g.  Women vs. Men, sociodemographic variables between countries).  The study sample should be described in sufficient detail so that other researchers can determine if it is comparable to the population of interest to them.

**86. Was data analysis conducted with sufficient coverage of the identified sample?**

Coverage bias can occur when not all subgroups of the identified sample respond at the same rate. For instance, you may have a very high response rate overall for your study, but the response rate for a certain subgroup (i.e. older adults) may be quite low.

**87. Were valid methods used for the identification of the condition?**

Here we are looking for measurement or classification bias.  Many health problems are not easily diagnosed or defined and some measures may not be capable of including or excluding appropriate levels or stages of the health problem. If the outcomes were assessed based on existing definitions or diagnostic criteria, then the answer to this question is likely to be yes. If the outcomes were assessed using observer reported, or self-reported scales, the risk of over- or under-reporting is increased, and objectivity is compromised. Importantly, determine if the measurement tools used were validated instruments as this has a significant impact on outcome assessment validity.

**88. Was the condition measured in a standard, reliable way for all participants?**

Considerable judgment is required to determine the presence of some health outcomes. Having established the validity of the outcome measurement instrument (see item 6 of this scale), it is important to establish how the measurement was conducted.  Were those involved in collecting data trained or educated in the use of the instrument/s? If there was more than one data collector, were they similar in terms of level of education, clinical or research experience, or level of responsibility in the piece of research being appraised? When there was more than one observer or collector, was there comparison of results from across the observers? Was the condition measured in the same way for all participants?

© JBI, 2020. All rights reserved. JBI grants use of these tools for research purposes only. All other enquiries should be sent to jbisynthesis@adelaide.edu.au.

89. **Was there appropriate statistical analysis?**

Importantly, the numerator and denominator should be clearly reported, and percentages should be given with confidence intervals. The methods section should be detailed enough for reviewers to identify the analytical technique used and how specific variables were measured. Additionally, it is also important to assess the appropriateness of the analytical strategy in terms of the assumptions associated with the approach as differing methods of analysis are based on differing assumptions about the data and how it will respond.

90. **Was the response rate adequate, and if not, was the low response rate managed appropriately?**

A large number of dropouts, refusals or "not founds" amongst selected subjects may diminish a study's validity, as can a low response rates for survey studies. The authors should clearly discuss the response rate and any reasons for non-response and compare persons in the study to those not in the study, particularly with regards to their socio-demographic characteristics. If reasons for non-response appear to be unrelated to the outcome measured and the characteristics of non-responders are comparable to those who do respond in the study (addressed in question 5, coverage bias), the researchers may be able to justify a more modest response rate.

# JBI CRITICAL APPRAISAL CHECKLIST FOR SYSTEMATIC REVIEWS AND RESEARCH SYNTHESES

Reviewer _________Jamie_____________________________________Brannigan______________________
Date________03/05/2021_______________

Author_______Waqar et al____________________________Year___2017___ Record Number_19/26

____

© JBI, 2020. All rights reserved. JBI grants use of these tools for research purposes only. All other enquiries should be sent to jbisynthesis@adelaide.edu.au.

| | Yes | No | Unclear | Not applicable |
|---|---|---|---|---|
| 164. Is the review question clearly and explicitly stated? | ☒ | ☐ | ☐ | ☐ |
| 165. Were the inclusion criteria appropriate for the review question? | ☒ | ☐ | ☐ | ☐ |
| 166. Was the search strategy appropriate? | ☒ | ☐ | ☐ | ☐ |
| 167. Were the sources and resources used to search for studies adequate? | ☒ | ☐ | ☐ | ☐ |
| 168. Were the criteria for appraising studies appropriate? | ☒ | ☐ | ☐ | ☐ |
| 169. Was critical appraisal conducted by two or more reviewers independently? | ☐ | ☐ | ☒ | ☐ |
| 170. Were there methods to minimize errors in data extraction? | ☐ | ☒ | ☐ | ☐ |
| 171. Were the methods used to combine studies appropriate? | ☐ | ☐ | ☒ | ☐ |
| 172. Was the likelihood of publication bias assessed? | ☒ | ☐ | ☐ | ☐ |
| 173. Were recommendations for policy and/or practice supported by the reported data? | ☒ | ☐ | ☐ | ☐ |
| 174. Were the specific directives for new research appropriate? | ☒ | ☐ | ☐ | ☐ |

Overall appraisal: Include ☒ Exclude ☐ Seek further info ☐

Comments (Including reason for exclusion)

It appears as if a second reviewer was only involved for the title/abstract screen. Combination of studies appears to have been in the form of simple pooling. Appropriate for inclusion.

© JBI, 2020. All rights reserved. JBI grants use of these tools for research purposes only. All other enquiries should be sent to jbisynthesis@adelaide.edu.au.

# JBI CRITICAL APPRAISAL CHECKLIST FOR SYSTEMATIC REVIEWS AND RESEARCH SYNTHESIS

How to cite: *Aromataris E, Fernandez R, Godfrey C, Holly C, Kahlil H, Tungpunkom P. Summarizing systematic reviews: methodological development, conduct and reporting of an Umbrella review approach. Int J Evid Based Healthc. 2015;13(3):132-40.*

When conducting an umbrella review using the JBI method, the critical appraisal instrument for Systematic Reviews should be used.

The primary and secondary reviewer should discuss each item in the appraisal instrument for each study included in their review. In particular, discussions should focus on what is considered acceptable to the aims of the review in terms of the specific study characteristics. When appraising systematic reviews this discussion may include issues such as what represents an adequate search strategy or appropriate methods of synthesis. The reviewers should be clear on what constitutes acceptable levels of information to allocate a positive appraisal compared with a negative, or response of “unclear”. This discussion should ideally take place before the reviewers independently conduct the appraisal.

Within umbrella reviews, quantitative or qualitative systematic reviews may be incorporated, as well as meta-analyses of existing research. There are 11 questions to guide the appraisal of systematic reviews or meta-analyses. Each question should be answered as “yes”, “no”, or “unclear”. Not applicable “NA” is also provided as an option and may be appropriate in rare instances.

### 34. Is the review question clearly and explicitly stated?

The review question is an essential step in the systematic review process. A well-articulated question defines the scope of the review and aids in the development of the search strategy to locate the relevant evidence. An explicitly stated question, formulated around its PICO (Population, Intervention, Comparator, Outcome) elements aids both the review team in the conduct of the review and the reader in determining if the review has achieved its objectives. Ideally the review question should be articulated in a published protocol; however this will not always be the case with many reviews that are located.

### 35. Were the inclusion criteria appropriate for the review question?

The inclusion criteria should be identifiable from, and match the review question. The necessary elements of the PICO should be explicit and clearly defined. The inclusion criteria should be detailed and the included reviews should clearly be eligible when matched against the stated inclusion criteria. Appraisers of meta-analyses will find that inclusion criteria may include criteria around the ability to conduct statistical analyses which would not be the norm for a systematic review. The types of included studies should be relevant to the review question, for example, an umbrella review aiming to summarize a range of effective non-pharmacological interventions for aggressive behaviors amongst elderly patients with dementia will limit itself to including systematic reviews and meta-analyses that synthesize quantitative studies assessing the various interventions; qualitative or economic reviews would not be included.

### 36. Was the search strategy appropriate?

A systematic review should provide evidence of the search strategy that has been used to locate the evidence. This may be found in the methods section of the review report in some cases, or as an appendix that may be provided as supplementary information to the review publication. A systematic review should present a clear search strategy that addresses each of the identifiable PICO components of the review question. Some reviews may also provide a description of the approach to searching and how the terms that were ultimately used were derived, though due to limits on word counts in journals this may be more the norm in online only publications. There

© JBI, 2020. All rights reserved. JBI grants use of these tools for research purposes only. All other enquiries should be sent to jbisynthesis@adelaide.edu.au.

should be evidence of logical and relevant keywords and terms and also evidence that Subject

Headings and Indexing terms have been used in the conduct of the search. Limits on the search should also be considered and their potential impact; for example, if a date limit was used, was this appropriate and/or justified? If only English language studies were included, will such a language bias have an impact on the review? The response to these considerations will depend, in part, on the review question.

## 37. Were the sources and resources used to search for studies adequate?

A systematic review should attempt to identify "all" the available evidence and as such there should be evidence of a comprehensive search strategy. Multiple electronic databases should be searched including major bibliographic citation databases such as MEDLINE and CINAHL. Ideally, other databases that are relevant to the review question should also be searched, for example, a systematic review with a question about a physical therapy intervention should also look to search the PEDro database, whilst a review focusing on an educational intervention should also search the ERIC. Reviews of effectiveness should aim to search trial registries. A comprehensive search is the ideal way to minimize publication bias, as a result, a well conducted systematic review should also attempt to search for grey literature, or "unpublished" studies; this may involve searching websites relevant to the review question, or thesis repositories.

## 38. Were the criteria for appraising studies appropriate?

The systematic review should present a clear statement that critical appraisal was conducted and provide the details of the items that were used to assess the included studies. This may be presented in the methods of the review, as an appendix of supplementary information, or as a reference to a source that can be located. The tools or instruments used should be appropriate for the review question asked and the type of research conducted. For example, a systematic review of effectiveness should present a tool or instrument that addresses aspects of validity for experimental studies and randomized controlled trials such as randomization and blinding – if the review includes observational research to answer the same question a different tool would be more appropriate. Similarly, a review assessing diagnostic test accuracy may refer to the recognized QUADAS[1] tool.

## 39. Was critical appraisal conducted by two or more reviewers independently?

Critical appraisal or some similar assessment of the quality of the literature included in a systematic review is essential. A key characteristic to minimize bias or systematic error in the conduct of a systematic review is to have the critical appraisal of the included studies completed independently and in duplicate by members of the review team. The systematic review should present a clear statement that critical appraisal was conducted by at least two reviewers working independently from each other and conferring where necessary to reach decision regarding study quality and eligibility on the basis of quality.

## 40. Were there methods to minimize errors in data extraction?

Efforts made by review authors during data extraction can also minimize bias or systematic errors in the conduct of a systematic review. Strategies to minimize bias may include conducting all data extraction in duplicate and independently, using specific tools or instruments to guide data extraction and some evidence of piloting or training around their use.

## 41. Were the methods used to combine studies appropriate?

© JBI, 2020. All rights reserved. JBI grants use of these tools for research purposes only. All other enquiries should be sent to jbisynthesis@adelaide.edu.au.

A synthesis of the evidence is a key feature of a systematic review. The synthesis that is presented should be appropriate for the review question and the stated type of systematic review and evidence it refers to. If a meta-analysis has been conducted this needs to be reviewed carefully.

Was it appropriate to combine the studies? Have the reviewers assessed heterogeneity statistically and provided some explanation for heterogeneity that may be present? Often, where heterogeneous studies are included in the systematic review, narrative synthesis will be an appropriate method for presenting the results of multiple studies. If a qualitative review, are the methods that have been used to synthesize findings congruent with the stated methodology of the review? Is there adequate descriptive and explanatory information to support the final synthesized findings that have been constructed from the findings sourced from the original research?

### 42. Was the likelihood of publication bias assessed?

As mentioned, a comprehensive search strategy is the best means by which a review author may alleviate the impact of publication bias on the results of the review. Reviews may also present statistical tests such as Egger's test or funnel plots to also assess the potential presence of publication bias and its potential impact on the results of the review. This question will not be applicable to systematic reviews of qualitative evidence.

### 43. Were recommendations for policy and/or practice supported by the reported data?

Whilst the first nine (9) questions specifically look to identify potential bias in the conduct of a systematic review, the final questions are more indictors of review quality rather than validity. Ideally a review should present recommendations for policy and practice. Where these recommendations are made there should be a clear link to the results of the review. Is there evidence that the strength of the findings and the quality of the research been considered in the formulation of review recommendations?

### 44. Were the specific directives for new research appropriate?

The systematic review process is recognized for its ability to identify where gaps in the research, or knowledge base, around a particular topic exist. Most systematic review authors will provide some indication, often in the discussion section of the report, of where future research direction should lie. Where evidence is scarce or sample sizes that support overall estimates of effect are small and effect estimates are imprecise, repeating similar research to those identified by the review may be necessary and appropriate. In other instances, the case for new research questions to investigate the topic may be warranted.

# REFERENCES

4. Whiting P, Rutjes AWS, Reitsma JB, Bossuyt PMM, Kleijnen J. The development of QUADAS: a tool for the quality assessment of studies of diagnostic accuracy included in systematic reviews. BMC Medical Research Methodology. 2003;3:25 doi:10.1186/1471-2288-3-25.

© JBI, 2020. All rights reserved. JBI grants use of these tools for research purposes only. All other enquiries should be sent to jbisynthesis@adelaide.edu.au.

# JBI CRITICAL APPRAISAL CHECKLIST FOR SYSTEMATIC REVIEWS AND RESEARCH SYNTHESES

Reviewer __________Jamie Brannigan_________________Date_________03/05/2021________________

Author________Webber-Jones et al_______________________Year___2002___ Record Number_20/26____

| | Yes | No | Unclear | Not applicable |
|---|---|---|---|---|
| 175. Is the review question clearly and explicitly stated? | ☐ | ☒ | ☐ | ☐ |
| 176. Were the inclusion criteria appropriate for the review question? | ☐ | ☒ | ☐ | ☐ |
| 177. Was the search strategy appropriate? | ☐ | ☐ | ☐ | ☒ |
| 178. Were the sources and resources used to search for studies adequate? | ☐ | ☐ | ☐ | ☒ |
| 179. Were the criteria for appraising studies appropriate? | ☐ | ☐ | ☐ | ☒ |
| 180. Was critical appraisal conducted by two or more reviewers independently? | ☐ | ☐ | ☐ | ☒ |
| 181. Were there methods to minimize errors in data extraction? | ☐ | ☐ | ☐ | ☒ |
| 182. Were the methods used to combine studies appropriate? | ☐ | ☐ | ☐ | ☒ |
| 183. Was the likelihood of publication bias assessed? | ☐ | ☒ | ☐ | ☐ |
| 184. Were recommendations for policy and/or practice supported by the reported data? | ☒ | ☐ | ☐ | ☐ |
| 185. Were the specific directives for new research appropriate? | ☒ | ☐ | ☐ | ☐ |

Overall appraisal: Include ☒ Exclude ☐ Seek further info ☐

Comments (Including reason for exclusion)

This review was not systematic. There is no quantitative synthesis. The extensive narrative is appropriate for discussion, however a reproducible literature search would have been preferable.

# JBI CRITICAL APPRAISAL CHECKLIST FOR SYSTEMATIC REVIEWS AND RESEARCH SYNTHESIS

How to cite: *Aromataris E, Fernandez R, Godfrey C, Holly C, Kahlil H, Tungpunkom P. Summarizing systematic reviews: methodological development, conduct and reporting of an Umbrella review approach. Int J Evid Based Healthc. 2015;13(3):132-40.*

When conducting an umbrella review using the JBI method, the critical appraisal instrument for Systematic Reviews should be used.

The primary and secondary reviewer should discuss each item in the appraisal instrument for each study included in their review. In particular, discussions should focus on what is considered acceptable to the aims of the review in terms of the specific study characteristics. When appraising systematic reviews this discussion may include issues such as what represents an adequate search strategy or appropriate methods of synthesis. The reviewers should be clear on what constitutes acceptable levels of information to allocate a positive appraisal compared with a negative, or response of “unclear”. This discussion should ideally take place before the reviewers independently conduct the appraisal.

Within umbrella reviews, quantitative or qualitative systematic reviews may be incorporated, as well as meta-analyses of existing research. There are 11 questions to guide the appraisal of systematic reviews or meta-analyses. Each question should be answered as “yes”, “no”, or “unclear”. Not applicable “NA” is also provided as an option and may be appropriate in rare instances.

### 45. Is the review question clearly and explicitly stated?

The review question is an essential step in the systematic review process. A well-articulated question defines the scope of the review and aids in the development of the search strategy to locate the relevant evidence. An explicitly stated question, formulated around its PICO (Population, Intervention, Comparator, Outcome) elements aids both the review team in the conduct of the review and the reader in determining if the review has achieved its objectives. Ideally the review question should be articulated in a published protocol; however this will not always be the case with many reviews that are located.

### 46. Were the inclusion criteria appropriate for the review question?

The inclusion criteria should be identifiable from, and match the review question. The necessary elements of the PICO should be explicit and clearly defined. The inclusion criteria should be detailed and the included reviews should clearly be eligible when matched against the stated inclusion criteria. Appraisers of meta-analyses will find that inclusion criteria may include criteria around the ability to conduct statistical analyses which would not be the norm for a systematic review. The types of included studies should be relevant to the review question, for example, an umbrella review aiming to summarize a range of effective non-pharmacological interventions for aggressive behaviors amongst elderly patients with dementia will limit itself to including systematic reviews and meta-analyses that synthesize quantitative studies assessing the various interventions; qualitative or economic reviews would not be included.

### 47. Was the search strategy appropriate?

A systematic review should provide evidence of the search strategy that has been used to locate the evidence. This may be found in the methods section of the review report in some cases, or as an appendix that may be provided as supplementary information to the review publication. A systematic review should present a clear search strategy that addresses each of the identifiable PICO components of the review question. Some reviews may also provide a description of the approach to searching and how the terms that were ultimately used were derived, though due to limits on word counts in journals this may be more the norm in online only publications. There

© JBI, 2020. All rights reserved. JBI grants use of these tools for research purposes only. All other enquiries should be sent to jbisynthesis@adelaide.edu.au.

should be evidence of logical and relevant keywords and terms and also evidence that Subject

Headings and Indexing terms have been used in the conduct of the search. Limits on the search should also be considered and their potential impact; for example, if a date limit was used, was this appropriate and/or justified? If only English language studies were included, will such a language bias have an impact on the review? The response to these considerations will depend, in part, on the review question.

## 48. Were the sources and resources used to search for studies adequate?

A systematic review should attempt to identify "all" the available evidence and as such there should be evidence of a comprehensive search strategy. Multiple electronic databases should be searched including major bibliographic citation databases such as MEDLINE and CINAHL. Ideally, other databases that are relevant to the review question should also be searched, for example, a systematic review with a question about a physical therapy intervention should also look to search the PEDro database, whilst a review focusing on an educational intervention should also search the ERIC. Reviews of effectiveness should aim to search trial registries. A comprehensive search is the ideal way to minimize publication bias, as a result, a well conducted systematic review should also attempt to search for grey literature, or "unpublished" studies; this may involve searching websites relevant to the review question, or thesis repositories.

## 49. Were the criteria for appraising studies appropriate?

The systematic review should present a clear statement that critical appraisal was conducted and provide the details of the items that were used to assess the included studies. This may be presented in the methods of the review, as an appendix of supplementary information, or as a reference to a source that can be located. The tools or instruments used should be appropriate for the review question asked and the type of research conducted. For example, a systematic review of effectiveness should present a tool or instrument that addresses aspects of validity for experimental studies and randomized controlled trials such as randomization and blinding – if the review includes observational research to answer the same question a different tool would be more appropriate. Similarly, a review assessing diagnostic test accuracy may refer to the recognized QUADAS[1] tool.

## 50. Was critical appraisal conducted by two or more reviewers independently?

Critical appraisal or some similar assessment of the quality of the literature included in a systematic review is essential. A key characteristic to minimize bias or systematic error in the conduct of a systematic review is to have the critical appraisal of the included studies completed independently and in duplicate by members of the review team. The systematic review should present a clear statement that critical appraisal was conducted by at least two reviewers working independently from each other and conferring where necessary to reach decision regarding study quality and eligibility on the basis of quality.

## 51. Were there methods to minimize errors in data extraction?

Efforts made by review authors during data extraction can also minimize bias or systematic errors in the conduct of a systematic review. Strategies to minimize bias may include conducting all data extraction in duplicate and independently, using specific tools or instruments to guide data extraction and some evidence of piloting or training around their use.

## 52. Were the methods used to combine studies appropriate?

© JBI, 2020. All rights reserved. JBI grants use of these tools for research purposes only. All other enquiries should be sent to jbisynthesis@adelaide.edu.au.

A synthesis of the evidence is a key feature of a systematic review. The synthesis that is presented should be appropriate for the review question and the stated type of systematic review and evidence it refers to. If a meta-analysis has been conducted this needs to be reviewed carefully.

Was it appropriate to combine the studies? Have the reviewers assessed heterogeneity statistically and provided some explanation for heterogeneity that may be present? Often, where heterogeneous studies are included in the systematic review, narrative synthesis will be an appropriate method for presenting the results of multiple studies. If a qualitative review, are the methods that have been used to synthesize findings congruent with the stated methodology of the review? Is there adequate descriptive and explanatory information to support the final synthesized findings that have been constructed from the findings sourced from the original research?

### 53. Was the likelihood of publication bias assessed?

As mentioned, a comprehensive search strategy is the best means by which a review author may alleviate the impact of publication bias on the results of the review. Reviews may also present statistical tests such as Egger's test or funnel plots to also assess the potential presence of publication bias and its potential impact on the results of the review. This question will not be applicable to systematic reviews of qualitative evidence.

### 54. Were recommendations for policy and/or practice supported by the reported data?

Whilst the first nine (9) questions specifically look to identify potential bias in the conduct of a systematic review, the final questions are more indictors of review quality rather than validity. Ideally a review should present recommendations for policy and practice. Where these recommendations are made there should be a clear link to the results of the review. Is there evidence that the strength of the findings and the quality of the research been considered in the formulation of review recommendations?

### 55. Were the specific directives for new research appropriate?

The systematic review process is recognized for its ability to identify where gaps in the research, or knowledge base, around a particular topic exist. Most systematic review authors will provide some indication, often in the discussion section of the report, of where future research direction should lie. Where evidence is scarce or sample sizes that support overall estimates of effect are small and effect estimates are imprecise, repeating similar research to those identified by the review may be necessary and appropriate. In other instances, the case for new research questions to investigate the topic may be warranted.

# REFERENCES

5. Whiting P, Rutjes AWS, Reitsma JB, Bossuyt PMM, Kleijnen J. The development of QUADAS: a tool for the quality assessment of studies of diagnostic accuracy included in systematic reviews. BMC Medical Research Methodology. 2003;3:25 doi:10.1186/1471-2288-3-25.

© JBI, 2020. All rights reserved. JBI grants use of these tools for research purposes only. All other enquiries should be sent to jbisynthesis@adelaide.edu.au.

# JBI CRITICAL APPRAISAL CHECKLIST FOR SYSTEMATIC REVIEWS AND RESEARCH SYNTHESES

Reviewer ____Jamie Brannigan________________Date________03/05/2021________________

Author_______Zarghooni et al______________________Year___2013___ Record Number_23/26____

| | Yes | No | Unclear | Not applicable |
|---|---|---|---|---|
| 186. Is the review question clearly and explicitly stated? | X | | | |
| 187. Were the inclusion criteria appropriate for the review question? | | | | X |
| 188. Was the search strategy appropriate? | | | | X |
| 189. Were the sources and resources used to search for studies adequate? | X | | | |
| 190. Were the criteria for appraising studies appropriate? | | | X | |
| 191. Was critical appraisal conducted by two or more reviewers independently? | | | X | |
| 192. Were there methods to minimize errors in data extraction? | | | X | |
| 193. Were the methods used to combine studies appropriate? | | | | X |
| 194. Was the likelihood of publication bias assessed? | | | X | |
| 195. Were recommendations for policy and/or practice supported by the reported data? | X | | | |
| 196. Were the specific directives for new research appropriate? | | | | X |

Overall appraisal: Include [X] Exclude [ ] Seek further info [ ]

Comments (Including reason for exclusion)

This review consisted of a selective search strategy, rather than systematic. There is hence limited rigour in search criteria, data extraction, critical appraisal and synthesis. Nonetheless, the narrative provided remains useful for inclusion.

# JBI CRITICAL APPRAISAL CHECKLIST FOR SYSTEMATIC REVIEWS AND RESEARCH SYNTHESIS

How to cite: *Aromataris E, Fernandez R, Godfrey C, Holly C, Kahlil H, Tungpunkom P. Summarizing systematic reviews: methodological development, conduct and reporting of an Umbrella review approach. Int J Evid Based Healthc. 2015;13(3):132-40.*

When conducting an umbrella review using the JBI method, the critical appraisal instrument for Systematic Reviews should be used.

The primary and secondary reviewer should discuss each item in the appraisal instrument for each study included in their review. In particular, discussions should focus on what is considered acceptable to the aims of the review in terms of the specific study characteristics. When appraising systematic reviews this discussion may include issues such as what represents an adequate search strategy or appropriate methods of synthesis. The reviewers should be clear on what constitutes acceptable levels of information to allocate a positive appraisal compared with a negative, or response of "unclear". This discussion should ideally take place before the reviewers independently conduct the appraisal.

Within umbrella reviews, quantitative or qualitative systematic reviews may be incorporated, as well as meta-analyses of existing research. There are 11 questions to guide the appraisal of systematic reviews or meta-analyses. Each question should be answered as "yes", "no", or "unclear". Not applicable "NA" is also provided as an option and may be appropriate in rare instances.

### 56. Is the review question clearly and explicitly stated?

The review question is an essential step in the systematic review process. A well-articulated question defines the scope of the review and aids in the development of the search strategy to locate the relevant evidence. An explicitly stated question, formulated around its PICO (Population, Intervention, Comparator, Outcome) elements aids both the review team in the conduct of the review and the reader in determining if the review has achieved its objectives. Ideally the review question should be articulated in a published protocol; however this will not always be the case with many reviews that are located.

### 57. Were the inclusion criteria appropriate for the review question?

The inclusion criteria should be identifiable from, and match the review question. The necessary elements of the PICO should be explicit and clearly defined. The inclusion criteria should be detailed and the included reviews should clearly be eligible when matched against the stated inclusion criteria. Appraisers of meta-analyses will find that inclusion criteria may include criteria around the ability to conduct statistical analyses which would not be the norm for a systematic review. The types of included studies should be relevant to the review question, for example, an umbrella review aiming to summarize a range of effective non-pharmacological interventions for aggressive behaviors amongst elderly patients with dementia will limit itself to including systematic reviews and meta-analyses that synthesize quantitative studies assessing the various interventions; qualitative or economic reviews would not be included.

### 58. Was the search strategy appropriate?

A systematic review should provide evidence of the search strategy that has been used to locate the evidence. This may be found in the methods section of the review report in some cases, or as an appendix that may be provided as supplementary information to the review publication. A systematic review should present a clear search strategy that addresses each of the identifiable PICO components of the review question. Some reviews may also provide a description of the approach to searching and how the terms that were ultimately used were derived, though due to limits on word counts in journals this may be more the norm in online only publications. There

© JBI, 2020. All rights reserved. JBI grants use of these tools for research purposes only. All other enquiries should be sent to jbisynthesis@adelaide.edu.au.

should be evidence of logical and relevant keywords and terms and also evidence that Subject

Headings and Indexing terms have been used in the conduct of the search. Limits on the search should also be considered and their potential impact; for example, if a date limit was used, was this appropriate and/or justified? If only English language studies were included, will such a language bias have an impact on the review? The response to these considerations will depend, in part, on the review question.

## 59. Were the sources and resources used to search for studies adequate?

A systematic review should attempt to identify "all" the available evidence and as such there should be evidence of a comprehensive search strategy. Multiple electronic databases should be searched including major bibliographic citation databases such as MEDLINE and CINAHL. Ideally, other databases that are relevant to the review question should also be searched, for example, a systematic review with a question about a physical therapy intervention should also look to search the PEDro database, whilst a review focusing on an educational intervention should also search the ERIC. Reviews of effectiveness should aim to search trial registries. A comprehensive search is the ideal way to minimize publication bias, as a result, a well conducted systematic review should also attempt to search for grey literature, or "unpublished" studies; this may involve searching websites relevant to the review question, or thesis repositories.

## 60. Were the criteria for appraising studies appropriate?

The systematic review should present a clear statement that critical appraisal was conducted and provide the details of the items that were used to assess the included studies. This may be presented in the methods of the review, as an appendix of supplementary information, or as a reference to a source that can be located. The tools or instruments used should be appropriate for the review question asked and the type of research conducted. For example, a systematic review of effectiveness should present a tool or instrument that addresses aspects of validity for experimental studies and randomized controlled trials such as randomization and blinding – if the review includes observational research to answer the same question a different tool would be more appropriate. Similarly, a review assessing diagnostic test accuracy may refer to the recognized QUADAS[1] tool.

## 61. Was critical appraisal conducted by two or more reviewers independently?

Critical appraisal or some similar assessment of the quality of the literature included in a systematic review is essential. A key characteristic to minimize bias or systematic error in the conduct of a systematic review is to have the critical appraisal of the included studies completed independently and in duplicate by members of the review team. The systematic review should present a clear statement that critical appraisal was conducted by at least two reviewers working independently from each other and conferring where necessary to reach decision regarding study quality and eligibility on the basis of quality.

## 62. Were there methods to minimize errors in data extraction?

Efforts made by review authors during data extraction can also minimize bias or systematic errors in the conduct of a systematic review. Strategies to minimize bias may include conducting all data extraction in duplicate and independently, using specific tools or instruments to guide data extraction and some evidence of piloting or training around their use.

## 63. Were the methods used to combine studies appropriate?

© JBI, 2020. All rights reserved. JBI grants use of these tools for research purposes only. All other enquiries should be sent to jbisynthesis@adelaide.edu.au.

A synthesis of the evidence is a key feature of a systematic review. The synthesis that is presented should be appropriate for the review question and the stated type of systematic review and evidence it refers to. If a meta-analysis has been conducted this needs to be reviewed carefully.

Was it appropriate to combine the studies? Have the reviewers assessed heterogeneity statistically and provided some explanation for heterogeneity that may be present? Often, where heterogeneous studies are included in the systematic review, narrative synthesis will be an appropriate method for presenting the results of multiple studies. If a qualitative review, are the methods that have been used to synthesize findings congruent with the stated methodology of the review? Is there adequate descriptive and explanatory information to support the final synthesized findings that have been constructed from the findings sourced from the original research?

### 64. Was the likelihood of publication bias assessed?

As mentioned, a comprehensive search strategy is the best means by which a review author may alleviate the impact of publication bias on the results of the review. Reviews may also present statistical tests such as Egger's test or funnel plots to also assess the potential presence of publication bias and its potential impact on the results of the review. This question will not be applicable to systematic reviews of qualitative evidence.

### 65. Were recommendations for policy and/or practice supported by the reported data?

Whilst the first nine (9) questions specifically look to identify potential bias in the conduct of a systematic review, the final questions are more indictors of review quality rather than validity. Ideally a review should present recommendations for policy and practice. Where these recommendations are made there should be a clear link to the results of the review. Is there evidence that the strength of the findings and the quality of the research been considered in the formulation of review recommendations?

### 66. Were the specific directives for new research appropriate?

The systematic review process is recognized for its ability to identify where gaps in the research, or knowledge base, around a particular topic exist. Most systematic review authors will provide some indication, often in the discussion section of the report, of where future research direction should lie. Where evidence is scarce or sample sizes that support overall estimates of effect are small and effect estimates are imprecise, repeating similar research to those identified by the review may be necessary and appropriate. In other instances, the case for new research questions to investigate the topic may be warranted.

## REFERENCES

6. Whiting P, Rutjes AWS, Reitsma JB, Bossuyt PMM, Kleijnen J. The development of QUADAS: a tool for the quality assessment of studies of diagnostic accuracy included in systematic reviews. BMC Medical Research Methodology. 2003;3:25 doi:10.1186/1471-2288-3-25.

© JBI, 2020. All rights reserved. JBI grants use of these tools for research purposes only. All other enquiries should be sent to jbisynthesis@adelaide.edu.au.

© JBI, 2020. All rights reserved. JBI grants use of these tools for research purposes only. All other enquiries should be sent to jbisynthesis@adelaide.edu.au.
