# Supplementary material for: Adverse Events Relating to Prolonged Hard Collar Immobilisation: A Systematic Review and Meta-Analysis
Source: Global Spine J. 2022 Mar 25;12(8):1968–78. doi: 10.1177/21925682221087194 (PMC9609519; doi:10.1177/21925682221087194)
Supplement: Supplementary material [file sj-pdf-4-gsj-10.1177_21925682221087194.pdf]

Supplementary Material 4. Summary table of included studies which are not cohort studies.

**Authors**	**Year**	**Country**	**Type of Study**	**Mean Patient Age, yrs**	**Collar Indication**	**Collar Type**	**Reported Complications**	**Mean Duration of Wear, Days**
Rodgers and Rodgers	1995	USA	Case report	30	Trauma	Vertebrace	Unilateral marginal mandibular nerve palsy (1 patient)	5
Ladny *et al.*	2020	Poland	Randomised trial	34	Clinical trial	Various	Increased optic nerve sheath diameter	5
Hewitt	1994	UK	Case report	19	Trauma	NR	Skin necrosis over mandible (1 patient)	2
Liew and Hill	1994	Australia	Case series	55.5	Trauma	NR	Skin breakdown/ulceration	8
Powers	1997	USA	Case series[a]	NR	Trauma	Various	Skin breakdown/ulceration	10.85
Webber-Jones *et al.*	2002	USA	Narrative review	NA	NR	Various	Skin breakdown/ulceration<br>Dysphagia<br>Increased ICP	NA
Waqar *et al.*	2014	UK	Systematic review	NR	Trauma (Odontoid fracture)	Various	Skin breakdown/ulceration<br>Medical complications (e.g., pneumonia)	NR
Peck *et al.*	2018	UK	Narrative review	NA[b]	Acute and chronic spinal disease	Various	Skin breakdown/ulceration<br>Increased ICP<br>Dysphagia<br>Respiratory compromise	NA
Lacey *et al.*	2019	USA	Narrative review	NA	Trauma	Various	Skin breakdown/ulceration	NA
Zarghooni *et al.*	2013	Germany	Narrative review	NA	Acute and chronic spinal disease	NR	Skin breakdown/ulceration<br>Dysphagia	NA
Dunham *et al.*	2008	USA	Narrative review	NA	Trauma	NR	Increased ICP	NA
Ertel *et al.*	2016	USA	Economic evaluation	NA	Trauma	NR	Skin breakdown/ulceration	NA

NR = Not reported, NA = Not applicable, ICP = Intracranial pressure
NB The included reviews primarily discuss the included cohort studies

[a]This study also incorporated a quality improvement exercise
[b]A specific focus on elderly patients.
